# Evolution of the Vertebrate Resistin Gene Family

**DOI:** 10.1371/journal.pone.0130188

**Published:** 2015-06-15

**Authors:** Qingda Hu, Huanran Tan, David M. Irwin

**Affiliations:** 1 Department of Laboratory Medicine and Pathobiology, University of Toronto, Toronto, Ontario, Canada; 2 Department of Pharmacology, Health Sciences Center, Peking University, Beijing, China; 3 Banting and Best Diabetes Centre, University of Toronto, Toronto, Ontario, Canada; University of Lausanne, SWITZERLAND

## Abstract

Resistin (encoded by *Retn*) was previously identified in rodents as a hormone associated with diabetes; however human resistin is instead linked to inflammation. Resistin is a member of a small gene family that includes the resistin-like peptides (encoded by *Retnl* genes) in mammals. Genomic searches of available genome sequences of diverse vertebrates and phylogenetic analyses were conducted to determine the size and origin of the resistin-like gene family. Genes encoding peptides similar to resistin were found in Mammalia, Sauria, Amphibia, and Actinistia (coelacanth, a lobe-finned fish), but not in Aves or fish from Actinopterygii, Chondrichthyes, or Agnatha. *Retnl* originated by duplication and transposition from *Retn* on the early mammalian lineage after divergence of the platypus, but before the placental and marsupial mammal divergence. The resistin-like gene family illustrates an instance where the locus of origin of duplicated genes can be identified, with *Retn* continuing to reside at this location. Mammalian species typically have a single copy *Retn* gene, but are much more variable in their numbers of *Retnl* genes, ranging from 0 to 9. Since *Retn* is located at the locus of origin, thus likely retained the ancestral expression pattern, largely maintained its copy number, and did not display accelerated evolution, we suggest that it is more likely to have maintained an ancestral function, while *Retnl*, which transposed to a new location, displays accelerated evolution, and shows greater variability in gene number, including gene loss, likely evolved new, but potentially lineage-specific, functions.

## Introduction

Resistin (*Retn*) was first identified in mice about 15 years ago, where it was identified as “adipose-tissue-specific secretory factor” (ADSF) [1] and “found in the inflammatory zone 3” (FIZZ3) [2], but acquired the name Resistin as it was associated with “resistance to insulin” [3]. Evidence for a role of resistin in glucose metabolism and insulin resistance included the observation that circulating levels of resistin in the blood of mice was down-regulated by anti-diabetic drugs, and that experimental modification of circulating resistin levels affected blood glucose and insulin function [3]. However, when human resistin (*RETN*) was characterized, it was found to be expressed in macrophages rather than adipose tissue [4], and the levels of circulating resistin are associated with inflammation rather than the amount of adipose tissue [5]. Further studies over the past 10 years have confirmed that rodent and human resistin have distinct expression patterns, and while the circulating levels of resistin may be increased in diabetes, these increases are likely mediated by different mechanisms in each species [6–8]. Currently the identity of the resistin receptor is unknown [9], although evidence has been presented that resistin can bind to three distinct receptors, an isoform of decorin [10], the ROR1 receptor [11], and the toll-like receptor 4 (TLR-4) [12]. If resistin interacts with multiple receptors, this might lead to multiple physiological targets. In addition to diabetes, elevated levels of resistin are associated with other human diseases, including cardiovascular diseases such as arteriosclerosis and heart failure [8,13,14] and cancer [9].

During the initial characterization of resistin, additional resistin-like genes were identified, with two members found in mice (originally named *Fizz1* and *Fizz2*, but also called Relmα and Relmβ (gene symbols: *Retnla* and *Retnlb*, respectively)) and one additional gene in humans (originally named *FIZZ1*, but also *RELM*β (gene symbol: *RETNLB*)) [1,15]. Here we use *RETNL* to refer to the mammalian resistin-like peptide genes (i.e., *Retnla*, *Retnlb*, and *Retnlg*), while *Retn*-like refers to duplicated *Retn* genes found in non-mammalian vertebrates. A phylogenomic study showed that the human and mouse *Retn* genes were orthologous, with human *RETN* residing on chromosome 19, while the rodent *Retnl* genes (*Retnla* and *Retnlb*) were clustered and resided on a genomic region orthologous to the human *RETNLB* gene on chromosome 3 [16]. This study also identified a second genomic sequence in the human genome (originally named hRELM-homo seq) with limited similarity to the human *RETNLB* gene sequence; however, it appeared that this sequence was not a functional gene as splicing signals were missing and no evidence for expression was found [16]. A latter study identified a third *Retnl* gene in rodent genomes (Relmγ, gene symbol: *Retnlg*), with this gene residing in the genomic region containing the *Retnla* and *Retnlb* genes [17]. The biological roles of the Retnl peptides are not well characterized, but have been reported to be involved in pulmonary remodeling [18,19], hyperlipidemia and arteriosclerosis [20,21], and intestinal adaptive immunity [22,23].

Despite the likely role of *RETN* and *RETNL* in human health, little is known about these genes except in humans, mice, and rats [6–9]. The evolutionary relationships among these genes are unresolved; except the orthology of the *Retn* genes [16]. For example, the phylogenetic relationships among the multiple rodent *Retnl* genes and human *RETNLB* is unclear (e.g., are rodent and human *Retnlb* more closely related to each other than to the rodent *Retnla* and *Retnlb* genes). It is also unknown when the *Retnl* gene family originated. Intriguingly, *Retn* was lost from the chicken and zebra finch genomes, along with a number of other adipokine genes, which might explain some of the differences in energy metabolism, and its regulation, between birds and mammals [24]. Here we investigated the evolution of *Retn* and *Retnl* genes in vertebrates and conclude that *Retn* is located and the locus of origin for the duplications and that *Retnl* originated via duplication and transposition to a new genomic location. In contrast to the *Retn* gene, which is generally single copy in mammals, duplication and inactivation frequently occurred during the evolutionary history of *Retnl*, which may reflect changing functions for the Retnl peptides.

## Materials and Methods

### Database searches

Resistin (*RETN*) and resistin-like (*RETNL*, e.g., *RETNLB*) coding and genomic sequences were identified in genomic databases maintained by the National Center for Biotechnology Information (NCBI, www.ncbi.nlm.nih.gov/projects/mapview/) and Ensembl (www.ensembl.org and www.pre.ensembl.org) in December 2014, using approaches we have used in previous studies [25,26,27]. Initial sequences were identified through searches of the Ensembl database using the gene symbols for resistin (*RETN*, human resistin; *Retn*, mouse resistin) and the resistin-like peptides (*Retnla*, mouse resistin-like alpha; *RETNLB*, human resistin-like beta; *Retnlb*, mouse resistin-like beta; *Retnlg*, mouse resistin-like gamma). Additional sequences were then identified in the NCBI and Ensembl databases via similarity searches with the tblastn algorithm [28] using a variety of diverse resistin and resistin-like protein sequences as queries. All sequences that had E-scores below 0.01 were examined. Sequences identified by the BLAST searches were used in reciprocal blastx searches of the human and mouse proteomes to ensure that their best matches were resistin or resistin-like peptide sequences. To identify coding sequences for *Retn* and *Retnl* genes from species where the gene had not been, or was incompletely, annotated, long genomic sequences that included the *Retn* or *Retnl* genes were aligned to the human or mouse genomic sequences with MultiPipMaker (pipmaker.bx.psu.edu/pipmaker/) [29,30]. Human and mouse sequences were used as masters for these alignments, with the locations of the exons and coding regions for these genes obtained from the Ensembl database. The identity and locations of repetitive elements in the human and mouse genomic sequences were identified using RepeatMasker [31]. Genomic alignments were used to refine the predicted potential coding regions of the genes. Potential pseudogenes were identified as sequences that failed to predict an open reading frame due to the presence of base changes that introduced stop codons, created frame shifts that disrupted the coding sequence, or disrupted splicing consensus sequences.

Sequences similar to *Retn* and/or *Retnl* genes were not found in several species. For these species, genomic sequences for genes in the predicted conserved genomic neighborhood were included in this analysis, an approach that can be used to identify genes with limited sequence similarity [32,33]. Genomes were searched for orthologs of the genes that flank the *Retn* or *Retnl* genes in diverse species (see results for details) and the genomic sequences adjacent to these genes were searched for similarity to *Retn* or *Retnl* genes. Genes were named (see S1 Table) to reflect their orthology-paralogy relationships, based on the phylogenetic analysis (see below) and sequence similarity (for incomplete sequences and pseudogenes). Multiple genes within a species were numbered arbitrarily.

### Phylogenetic analysis

Phylogenies of *Retn* and *Retnl* coding sequences were generated using full-length resistin and resistin-like sequences from diverse vertebrate species (see S1 Table and S1 Fig). *Retn* and *Retnl* coding sequences were aligned using MAFFT [34] as implemented at the Guidance web server site (http://guidance.tau.ac.il/) [35], using default parameters. Similar results were obtained if ClustalW [36] was used as the alignment program. DNA sequence alignments were based on codons to retain protein alignments. The reliability of the alignments was examined using Guidance [35] and trimmed alignments using sites that had values above the default cut-off of 0.93 were generated.

Phylogenetic trees of the *Retn* and *Retnl* sequences were generated using Bayesian methods with MrBayes 3.2.2 [37], maximum likelihood with PhyML 3.0 [38], and neighbor-joining distance approaches with MEGA6.0 [39]. Bayesian trees were generated from the coding sequences using parameters selected by hierarchical likelihood ratio tests with ModelTest version 3.8 [40], as implemented on the FindModel server (www.hiv.lanl.gov/content/sequence/findmodel/findmodel.html). MrBayes was run for 2,000,000 generations with four simultaneous Metropolis-coupled Monte Carlo Markov chains sampled every 100 generations. The average standard deviation of the split frequencies dropped to less than 0.02 for all analyses. The first 25% of the trees were discarded as burn-in with the remaining samples used to generate the consensus trees. Trace files generated by MrBayes were examined by Tracer (tree.bio.ed.ac.uk/software/tracer/) to verify if they had converged. Bootstrapped maximum likelihood trees, 100 replications, were generated with PhyML [38] on the PhyML webserver (www.atgc-montpellier.fr/phyml/) using parameters for the substitution model suggested by ModelTest. Maximum likelihood searches was initiated from trees generated by BIONJ and the best tree was identified after heuristic searches using the nearest neighbor interchange (NNI) algorithm. MEGA6.0 [39] was used to construct bootstrapped (1000 replications) neighbor-joining distance trees, using either Maximum Composite Likelihood distances for the DNA sequences or JTT distances for the protein sequences. Similar results were obtained if alternative outgroups (e.g., sequences from Coelacanth or Sauria) were used (results not shown).

With respect to orthology-paralogy issues, choice of outgroup, alignment method (MAFFT [34] or Clustal W [36]), or the use of full-length or trimmed (based on Guidance scores [35]) alignments had little influence on the key findings of these analyses. Methods that relied on shorter sequences (i.e., trimmed alignments or protein sequences) or simpler models of sequence evolution (i.e., neighbor-joining) tended to yield weaker support for the earlier diverging lineages, but none of our analyses were in significant conflict with the key inferences of our inferred phylogenies.

## Results and Discussion

### Distribution of *Retn* and *Retnl* genes

**Fig 1:**
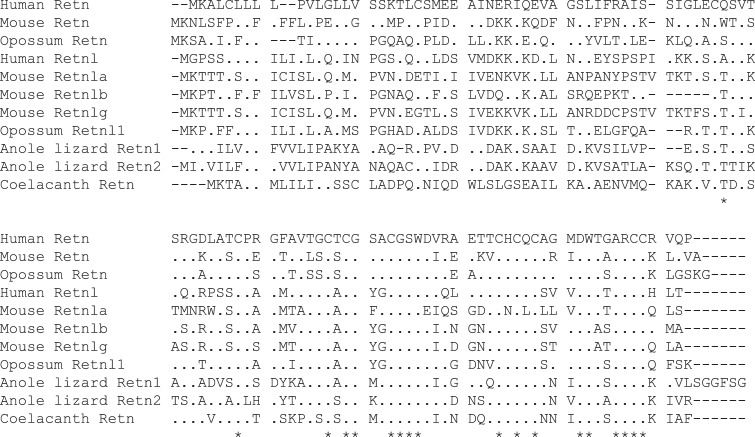
Alignment of Retn and Retnl protein sequences. Alignment of predicted resistin and resistin-like protein sequences from human, mouse, opossum, Anole lizard, and coelacanth (lobe-finned fish). The human Retn sequence is shown at the top in single letter code. Dots in the alignments represent identity to the human Retn sequence, with differences indicated in single letter code. Asterisks below the alignment identify residues that are perfectly conserved among all of the selected sequences.

Previously characterized *Retn* and *Retnl* genes contain 3 coding exons (see S2 Fig), with introns interrupting the coding region at homologous locations [41]. All of the *Retn* and *Retnl* genes identified here (S1 Table) have similar gene structures. Some differences in the exon organization of the untranslated regions of *Retn* genes have been reported [42], however, we did not attempt to identify untranslated exons in the *Retn* and *Retnl* genes, as these sequences typically evolve faster than coding sequence, thus are more difficult to detect using sequence conservation. Our searches of the genomes of 116 vertebrate species that were available in the Ensembl and NCBI genome databases in December 2014 resulted in the identification of 206 *Retn* or *Retnl* genes from a total of 84 species, of which 136 (from 78 species) possessed intact coding sequences that encoded potentially functional proteins (Table 1, S1 Table and S1 Fig). As illustrated in Fig 1, all of the coding regions predict proteins of similar length (approximately 110 amino acids, except the Chinese alligator *Retn1*, see below) with most of the cysteine residues in these cysteine-rich peptide sequences being at conserved locations.

**Table 1:** Numbers of *Retn* and *Retnl* genes found in groups of vertebrate species.

Vertebrate group	Species^1^	Intact	Incomplete^2^	Pseudogene^3^	Total
Mammalia	76/75/71	128	29	28	185
Aves	12/0/0	0	0	0	0
Sauria (except Aves)	7/7/6	7	11	1	19
Amphibia	1/1/0	0	1	0	1
Actinistia	1/1/1	1	0	0	1
Actinopterygii	17/0/0	0	0	0	0
Chondrichthyes	1/0/0	0	0	0	0
Agnatha	1/0/0	0	0	0	0
Total	116/84/78	136	41	29	206

^1^ Number of species examined / number of species with a *Retn* or *Retnl* gene / number of species with at least one intact *Retn* or *Retnl* coding sequence for each group.

^2^ Genomic sequences that fail to predict part of a *Retn* or *Retnl* gene sequence, possibly due to a gap in the genome assembly.

^3^ Sequence contains mutations that cause a frame shift or introduce premature stop codons in the *Retn* or *Retnl* coding sequence.

A total of 76 mammalian (Class Mammalia) genomes were available in the Ensembl or NCBI databases, and at least one sequence that encoded a *Retn* or *Retnl* sequence was found in 75 of these species (Table 1 and S1 Table). The only mammalian species where we failed to identify a sequence with similarity to *Retn* or *Retnl* was the sloth (S1 Table), a species with a low coverage (2.05X) genome assembly, thus it is possible that *Retn* and/or *Retnl* sequences exist in gaps in this genome sequence. A total of 185 putative *Retn* or *Retnl* genes were found in the 75 mammalian species that had at least one gene, with most species having two or more sequences. Of the 185 genomic sequences, 128 predict full-length coding sequences (representing 71 species), 28 are predicted to be pseudogenes as they contain mutations that prevent formation of an intact protein (see below for more details), and 29 are incomplete genes, where part of the gene sequence was missing (Table 1 and S1 Table). Most of the incomplete genes are from species with lower sequence coverage, thus the missing sequences potentially are in sequence gaps and therefore we expect that many of these genes are functional.

Distinct *Retn* and *Retnl* genes were found only in placental and marsupial mammals, and not in the platypus, the only monotreme mammal examined (S1 Table). Within placental and marsupial mammals, a total of 69 *Retn* genes were identified from a total of 67 species, with two closely related species (alpaca and camel) each having two *Retn* genes (S1 Table). We failed to find a *Retn* gene in 8 mammals (S1 Table). Among the 67 species that have *Retn* genes, 61 have intact coding sequences and 6 were incomplete. *Retn* pseudogenes were only found in the camel and alpaca, which are closely related (S1 Table). In both of these species an intact *Retn* gene was also found with the *Retn* pseudogenes being processed pseudogenes (see S3 Fig). In contrast to *Retn*, the number of *Retnl* genes in the genomes of placental and marsupial species varied from 0 genes (in 9 different species) to 9 genes in the prairie deer mouse (S1 Table). A total of 27 *Retnl* pseudogenes, from 26 species, were identified, with 13 of these coming from 12 species that did not otherwise have a potentially functional *Retnl* gene. Pseudogenes were generated by mutations that introduced in frame stop codons in 5 of these 12 species, including the marmoset that has two near identical *Retnl* pseudogenes that share the same inactivating in frame stop mutation (see S1 Table). *Retnl* genes from all 6 ruminant artiodactyls examined (cow, yak, river buffalo, chiru, sheep, and goat) shared the same disrupting mutation, the insertion of a Bov-tA2 repetitive DNA element into exon 1 (see S4 Fig). The last species that only had a *Retnl* pseudogene is the gibbon. While the gibbon was missing an intact *Retnl* gene, it did share the processed pseudogene (homologous to the hRELM-homo seq from humans [16]) that was inserted downstream of the *Retnl* gene in an early primate lineage (see S1 Table and S5 Fig). It is possible that an intact *Retnl* gene exits in a gap in the gibbon genome.

*Retn*-like genes were found in the genomes of the 7 non-avian species of clade Sauria (reptiles and birds) that have available genome sequences, with the number of genes ranging from 1 in the Chinese softshell turtle, to 4 in the Chinese and American alligators and the green sea turtle (Table 1 and S1 Table). Of the 7 species, intact *Retn*-like coding sequences were found in 6 of them. The two genes identified in the Burmese python, a species with a low coverage genome, were incomplete (S1 Table). Most of the *Retn*-like genes identified in these species were incomplete, and suggested that they were partial gene duplicates. Since most of the *Retn*-like sequences within each genome in these genomes were most similar to each other, this suggests that the majority of them were recent lineage-specific duplicates. A *Retn*-like gene, *Retn*2 from the American alligator, contained a base change that produces a pseudogene (S1 Table). Intriguingly, the only *Retn*-like gene with an altered gene structure was found in the Chinese alligator, where a duplicated exon 2 sequence was found within the *Retn2* gene, which results in a larger (but in frame) predicted protein product of 138 amino acid residues (see S2 Fig).

In contrast to the above, *Retn*-like sequences were not found in the 8 bird (Class Aves, within clade Sauria) genomes examined (Table 1 and S1 Table). This observation is consistent with a recent report of the loss of adipokine genes, including resistin, from the chicken and zebra finch genomes [24]. A single *Retn*-like gene was found in the one available amphibian (Class Amphibia) genome (*Xenopus tropicalis*), although it was incomplete as it was located at one end of a short genomic contig (Table 1 and S1 Table). The genome of the coelacanth (Subclass Actinistia), a lobe-finned fish that is more closely related to tetrapods (Superclass Tetrapoda) than to other fish [43,44], contained a single intact *Retn*-like gene (Table 1 and S1 Table). Searches of the genomes of 17 ray-finned fish (Class Actinopterygii), 1 cartilaginous fish (Class Chondrichthyes), and 1 jawless fish (Superclass Agnatha) (Table 1 and S1 Table) failed to identity any sequences similar to *Retn*, although this may simply reflect the sensitivity of the searches as the coelacanth sequence showed limited similarity.

### Phylogenetic relationships of *Retn* and *Retnl* genes

**Fig 2:**
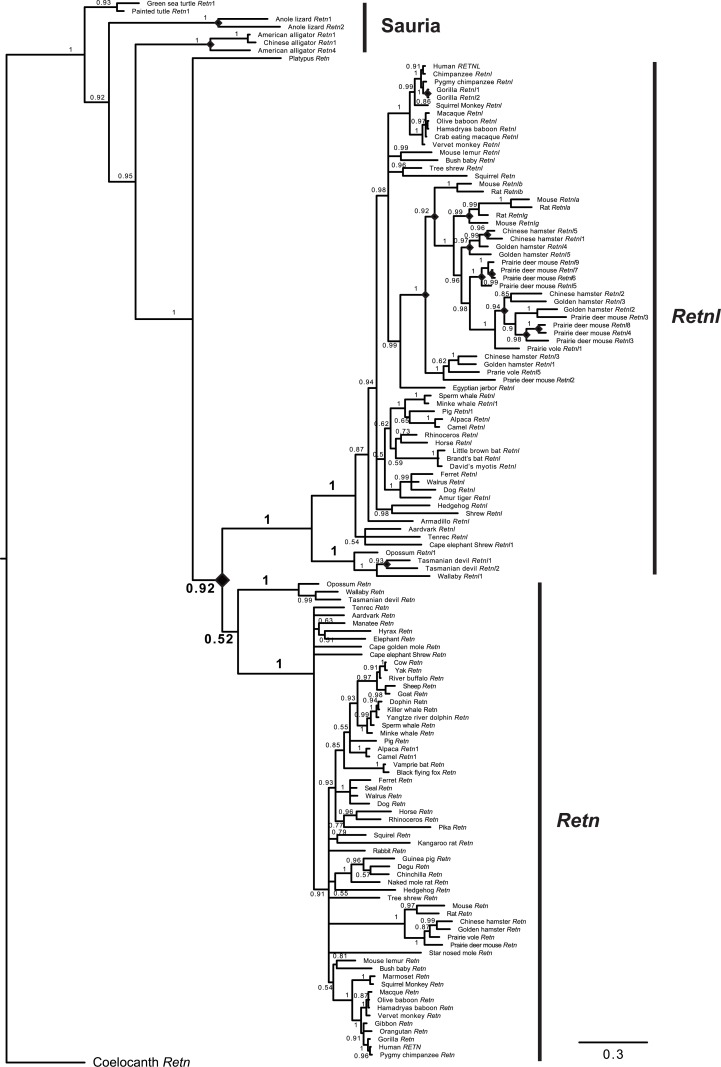
Phylogeny of *Retn* and *Retnl* genes. Phylogeny inferred by the Bayesian method for 136 *Retn* and *Retnl* sequences from diverse vertebrates. The phylogeny was rooted with the Coelacanth *Retn* sequence. Similar phylogenies were obtained if sequences from Sauria (coelacanth sequence not used) were used as the outgroup or if other methods were used (e.g., see S6 Fig). Numbers at the nodes indicate posterior probabilities, with those for the nodes in early mammalian evolution shown in bold. Branch lengths are proportional to the inferred amount of change, with the scale bar at the bottom. Diamonds indicate inferred gene duplication events. *Retnl* genes are shown in the upper part of the tree while *Retn* genes are below.

As described above, multiple *Retn* and/or *Retnl* gene sequences were identified in many vertebrate species (see S1 Table). To better understand the relationships among these genes, a phylogeny of these sequences was estimated (Fig 2). To obtain a phylogenetic tree with maximal support, only intact *Retn* and *Retnl* sequences (see S1 Fig), and codons that could be confidently aligned, were used. An alignment of 136 intact *Retn* and *Retnl* sequences was generated and trimmed using Guidance [35], and the phylogenetic relationships among the sequences was assessed using multiple approaches. The phylogeny inferred by the Bayesian approach using MrBayes 3.2 is shown in Fig 2. Similar phylogenies were obtained using other methods, including distance (results not shown) and maximum likelihood methods, with a maximum likelihood tree obtained using PhyML 3.0 shown in S6 Fig.

All of our phylogenetic analyses (Fig 2, S6 Fig, and results not shown) inferred a gene duplication that occurred early in mammalian evolution that resulted in two classes of *Retn*-like genes in mammals, the *Retn* and the *Retnl* genes. As shown in Fig 2, the phylogenetic analysis suggests that the duplication yielding *Retn* and *Retnl* occurred after the divergence of platypus from the other mammals, but before the divergence of placental and marsupial mammals. The phylogenies of mammalian species inferred by *Retn* and *Retnl* were roughly in accord with established species relationships [45,46], with differences likely due to the limited amount of phylogenetic information contained in these short sequences. Within mammals, no evidence for further duplication of the *Retn* gene was found (see Fig 2 and S6 Fig), although our genomic analysis (S1 Table) suggests that a *Retn* processed pseudogene was generated on the lineage leading the camel and alpaca. In contrast, multiple duplications of the *Retnl* gene are inferred from the phylogenetic analysis (Fig 2 and S6 Fig), with the genomic data (S1 Table) suggesting that additional duplications also occurred. The majority of the gene duplications occurred within rodents (see Fig 2, S7 and S8 Figs), but the phylogenetic analysis indicates that duplications also occurred on the lineages leading to the gorilla and within marsupials, while the genomic data suggests additional duplications within primates, cetaceans, Afrotheria, pig, dog, and cape elephant shrew. Additional duplications were detected within clade Sauria, with duplications in the lizard and alligator lineage illustrated in the phylogenetic trees (Fig 2 and S6 Fig), and additional duplication in the alligator and turtle lineages indicated by the genomic data (S1 Table).

### The mammalian *Retn* gene resides at the locus of origin for the gene family

The phylogenetic analysis of the *Retn* and *Retnl* sequences (Fig 2 and S6 Fig) showed that there were two types of *Retn*-like genes in mammals, *Retn* and *Retnl* (which include the previously named *Retnla*, *Retnlb*, and *Retnlg* genes). While the divergence of the *Retn* and *Retnl* genes occurred prior to the divergence of placental mammals from marsupials, the Bayesian and maximum likelihood analyses yielded different conclusion concerning the existence of a *Retn* ortholog in marsupial mammals. The Bayesian analysis suggests that both *Retn* and *Retnl* exist in marsupials; while the maximum likelihood analysis suggests that marsupials have ancient diverging *Retnl* genes and that the ortholog of the *Retn* gene was lost. To resolve this difference we examined the genomic neighborhoods of the *Retn* and *Retnl* genes.

**Fig 3:**
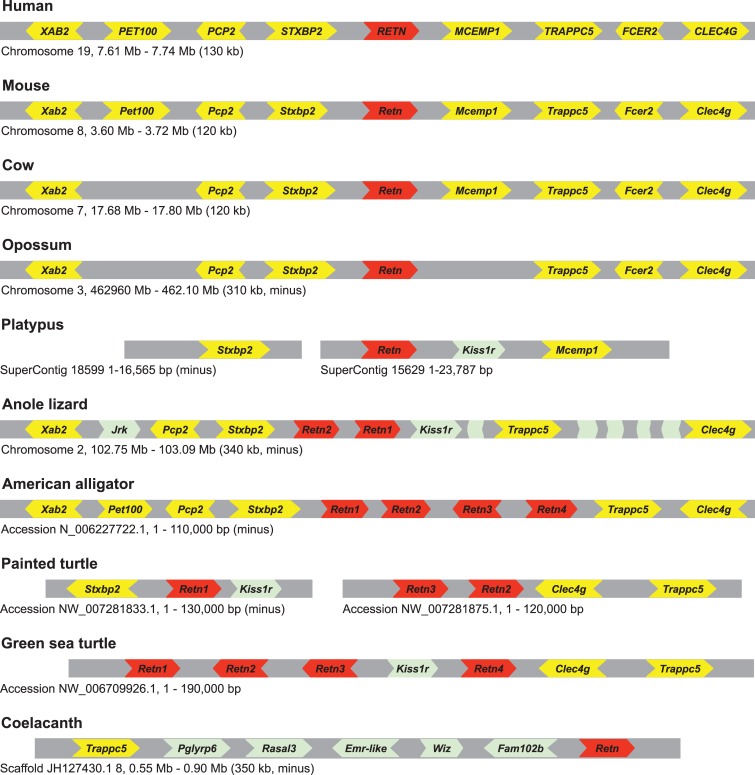
Genomic organization of genes near *Retn* genes of representative vertebrate species. *Retn* genes are labeled in red. Genes that share genomic location with human genes are labeled in yellow, while genes labeled in green are either lineage-specific genes or are found at a different genomic location in the human genome (genes without name do not have a human ortholog). The painted turtle genomic neighborhood is composed of two scaffolds that are likely adjacent. Chromosome, genomic scaffold, or sequence accession numbers, with approximate coordinates and size, of the displayed fragment are shown. See S1 Table for details on genomic locations of *Retn* genes. Gene sizes and distances between genes are not to scale. Arrowheads indicate direction of transcription. Gene symbols are: *Retn*, resistin; *Xab2*, XPA binding protein 2; *Pet100*, PET100 homolog; *Pcp2*, Purkinje cell protein 2; *Stxbp2*, Syntaxin binding protein 2; *Mcemp1*, Mast cell-expressed membrane protein 1; *Trappc5*, Trafficking protein particle complex 5; *Fcer2*, Fc fragment of IgE, low affinity II, receptor for (CD23); *Clec4g*, C-type lectin domain family 4, member G; *Kiss1r*, KISS1 receptor; *Jrk*, Jerky; *Pglyrp6*, Peptidoglycan recognition protein 6; *Rasal3*, RAS protein activator like 3; *Emr*-like, Egf-like module containing, mucin-like, hormone receptor-like; *Wiz*, Widely interspaced zinc finger motifs; and *Fam102b*, Family with sequence similarity 102, member B.

**Fig 4:**
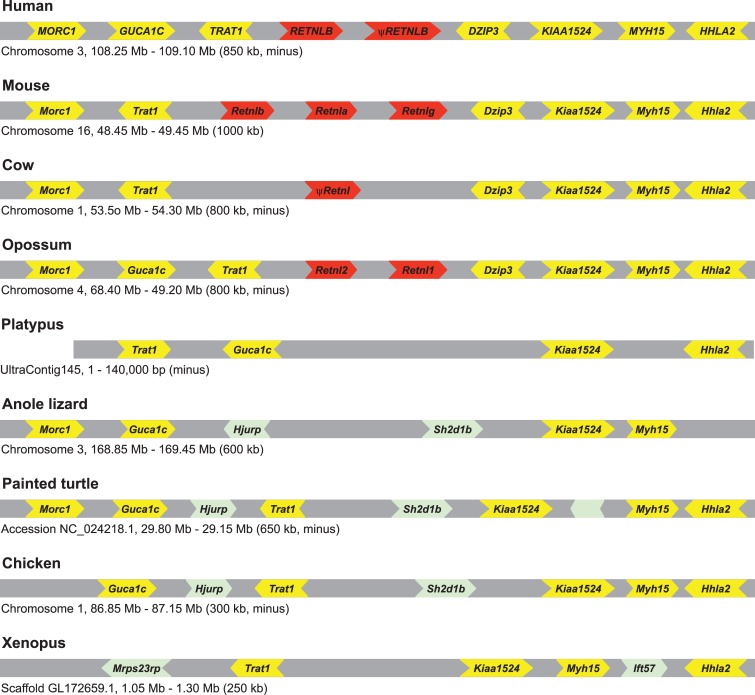
Genomic organization of genes near *Retnl* genes of representative vertebrate species. *Retnl* genes are labeled in red. Genes that share genomic location with human genes are labeled in black, while genes labeled in green are either lineage-specific genes or are found at a different genomic location in the human genome (genes without names do not have a human ortholog). Chromosome, genomic scaffold, or sequence accession numbers, with approximate coordinates and size, of the displayed fragment is shown. See S1 Table for details on genomic locations of *Retnl* genes. Gene sizes and distances between genes are not to scale. Arrowheads indicate direction of transcription. Gene symbols are: *Retnl*, resistin-like; *Morc1*, MORC family CW-type zinc finger 1; *Guca1c*, Guanylate cyclase activator 1C; *Trat1*, T cell receptor associated transmembrane adaptor 1; *Dzip3*, DAZ interacting zinc finger protein 3; *Kiaa1524*, KIAA1524; *Myh15*, Myosin, heavy chain 15; *Hhla2*, HERV-H LTR-associating 2; *Hjurp*, Holliday junction recognition protein; *Sh2d1b*, SH2 domain containing 1B; *Ift57*, Intraflagellar transport 57; and *Mrps23*, Mitochondrial ribosomal protein S23.

The human *RETN* and *RETNL* genes reside on different chromosomes with a third *RETN*-like sequence, a truncated processed pseudogene (see below for description), adjacent to *RETNL* (see S1 Table). The *STXBP2* and *MCEMP1* genes flank the human *RETN* gene on chromosome 19 (Fig 3). Putative *RETN* orthologs were inferred from the phylogenetic analysis shown in Fig 2 and their flanking genes were identified (Fig 3). As previously reported [24], and shown in Fig 3, many genes near the cow and mouse *Retn* genes are orthologous to the human genes flanking *RETN*, with similar results found for several other placental mammalian species that have well assembled genomes (results not shown). The putative opossum *Retn* ortholog was also found to be in a genomic neighborhood homologous to that of human *RETN* (Fig 3), demonstrating that the human and opossum *Retn* genes are indeed orthologous. A similar analysis of putative mammalian *RETNL* orthologs, including the opossum, yielded similar results, with orthologs inferred by the phylogenetic analysis shown in Fig 2 and S6 Fig being found in conserved genomic neighborhoods (Fig 4). These results indicate that the gene duplication that generated *Retn* and *Retnl* occurred prior to the divergence of placental and marsupial mammals.

**Fig 5:**
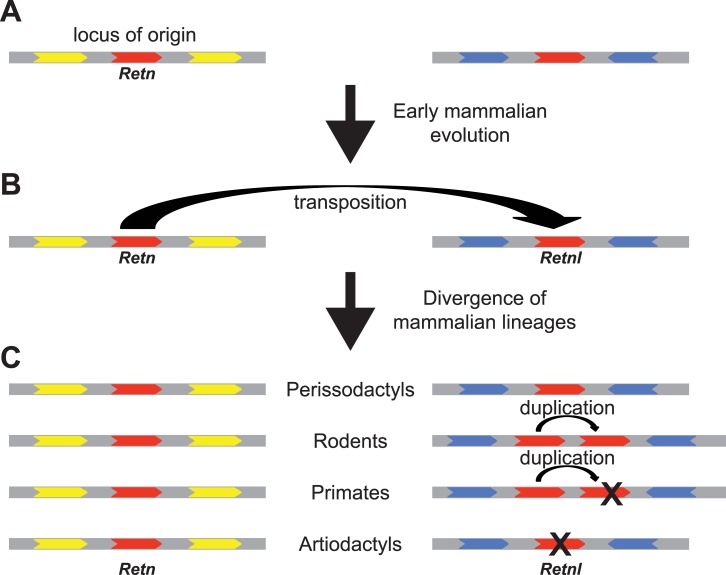
Model for the evolution of the resistin and resistin-like genes. *Retn* and *Retnl* genes are indicated by the red arrows, with the arrow pointing in the direction of transcription. Other genes are shown in yellow (locus of origin) or blue (location of inserted *Retnl* gene). Curved arrows indicated gene duplications that were either a transposition (to generate *Retnl*) or tandem, on the rodent and primate lineages. X’s indicate inactivating mutations in the primate and artiodactyl genes that generate pseudogenes. (A). In the ancestor to mammals, *Retn* was located in the locus of origin. (B). On an early mammalian lineage, prior to the placental mammal-marsupial divergence, a copy of *Retn* was transposed to a new genomic location to generate the *Retnl* gene. The transposition likely allowed *Retnl* to acquire a novel expression pattern. (C). *Retn* and *Retnl* genes have different fates on divergent mammalian lineages. While *Retn* remained as a single copy intact gene on different mammalian lineages, *Retnl* had different fates, raising the possibility that it acquired lineage-specific functions. *Retnl* remained as a single copy gene in perissodactyls, duplicated intact (potentially functional) copies in rodents, duplicated and generated a pseudogene in primates, or was inactivated in artiodactyls.

Since the mammalian *Retn* and *Retnl* reside on different chromosomes, it is possible that the genomic neighborhoods may also help determine whether one of these two genes is located at the locus of origin. *Retn*-like genes in species that do not have both a *Retn* and a *Retnl* gene should have the gene at the original genomic locus of origin. Our phylogenetic analysis indicated that the platypus *Retn* gene diverged prior to the *Retn*-*Retnl* duplication event (Fig 2 and S6 Fig), and thus could be in the locus of origin. The platypus *Retn* gene is located on a short genomic contig (24kb) that also contains the *Mcemp*1 gene, suggesting that the genomic neighborhood is similar to that of *Retn* in other mammals (Fig 3). A platypus genomic neighborhood homologous to the *Retnl* genomic neighborhood was identified that lacks a *Retnl* gene (Fig 4). These results suggest that the locus of origin was the *Retn* genomic neighborhood and that during duplication of this gene yielded a daughter *Retnl* gene that transposed to a new genomic neighborhood. However, since both posterior probabilities (Fig 2) and bootstrapping (S6 Fig) provide good, but not excellent, support for the outgroup placement of the platypus *Retn* gene sequence, we cannot exclude the possibility that the gene duplication occurred earlier in mammalian evolution, with the platypus subsequently losing the *Retnl* gene. To exclude this possibility, we examined additional outgroup species also have a *Retn* gene, and have a divergence prior to the *Retn*-*Retnl* duplication that is very strongly supported (Fig 2 and S6 Fig). Analysis of the genomic neighborhoods surrounding *Retn* genes in species from clade Sauria shows that many genes are shared with mammalian *Retn* gene neighborhoods (Fig 3), and that the genomic neighborhoods homologous to the mammalian *Retnl* gene neighborhood lack *Retn*-like genes (Fig 4). The *Xenopus Retn* gene was on a short genomic contig (<11 kb) that did not contain any additional genes thus provided no information on the ancestral gene order. Gene order near the *Retn* gene in coelacanth differed greatly from that of other species with *Retn* genes, with only the *Trappc5* being in the same genomic neighborhood (Fig 3). Similarly, a conserved genomic neighborhood could not be found in other fish, as the closely linked genes tended to be dispersed among different chromosomes (e.g., in zebrafish: *Stxbp2*, Chromosome 3; *Trappc1*, Chromosome 1; *Kiss1r*, Chromosome 5). Our analyses indicates that a conserved genomic neighborhood is shared between mammals and species of clade Sauria, with the neighborhood surrounding the non-mammalian *Retn*-like genes supporting the conclusion that the mammalian *Retn* gene is located at locus of origin and that *Retnl* was transposed to a new genomic location (see Fig 5 for evolutionary model).

### Evolution of *Retn* and *Retnl* gene function

Since *Retnl* originated from *Retn* via duplication and transposition to a new genomic location, while *Retn* remained in the locus of origin, raises the possibility that *Retn* retained an ancestral function while *Retnl* gained new function (Fig 5). Since *Retnl* transposed to a new genomic location, its expression pattern may have changed due to the loss of regulatory elements that were not duplicated with the gene or due to the gain of new regulatory elements from its new genomic neighborhood (see Fig 5). A change in the site, time, or level of expression of the gene provides opportunities to change gene function. If *Retnl* evolved a new function, then one might expect an increase in the rate of *Retnl* sequence evolution after the initial gene duplication event, as selection should have been relaxed on this redundant copy [47,48]. Intriguingly, the phylogenetic trees of the *Retn* and *Retnl* sequences suggest that the lineage leading from the common ancestor of the *Retn* and *Retnl* genes (i.e., duplication event) to the common ancestor of marsupial and placental *Retnl* genes is longer than the lineage form the common ancestor of *Retn* and *Retnl* genes and that common ancestor of marsupial and placental *Retn* genes (see Fig 2 and S6 Fig). Since both lineages represent the same amount of time (gene duplication to species divergence), the difference in branch lengths suggests more rapid evolution on the *Retnl* lineage, which could be due to relaxed selection [47,48].

Additional evidence for *Retnl* evolving new roles is based on the variability of the copy number of the *Retnl* gene in diverse mammalian genomes (see Fig 5). As mentioned above, searches of mammalian genomes for *Retn* and *Retnl* genes found that *Retn* is typically found as a single copy gene, suggesting that maintaining a single copy is essential. In contrast, the number of *Retnl* genes is much more was variable (S1 Table). While we failed to find *Retnl* genes in 9 mammalian species (S1 Table), some of these may have genes that exist in gaps in their genome assemblies. In addition to these 9 species, 12 other mammalian species (including species within primates, rodents, artiodactyls, and bats, see S1 Table) only have *Retnl* pseudogenes, which suggests that this gene has been lost in multiple species, and thus is not essential. In rodents, however, multiple intact *Retnl* genes were found in many species (S1 Table). The multiple rodent *Retnl* genes originated on the rodent lineage (see Fig 2, S6 and S7 Figs) and were found as a cluster of duplicated genes (see Fig 4, S8 Fig and S1 Table), suggesting they were generated by tandem gene duplication. Initial duplication of the *Retnl* gene in rodents must have been early in rodent evolution, with multiple subsequent duplications, and their phylogenies (S6 and S7 Figs) suggest the possibility of concerted evolution [49,50]. Maintenance of divergent *Retnl* genes (e.g., *Retnla* and *Retnlg* in mice and rats) may suggest that duplicate *Retnl* genes have differing functions, consistent with these genes adapting to new, and potentially lineage-specific, functions. Rodent *Retnl* gene sequences also have longer branch lengths in the phylogenies (Fig 2 and S6 Fig), implying that they have accumulated a larger number of changes. This would also suggest that the rodent *Retnl* genes have experienced relaxed selection or adaptive evolution, both of which would be consistent with lineage-specific changes in gene function.

Intriguingly, among the *Retn* sequences identified in this study, the rodent *Retn* sequences show the greatest amount of change (see Fig 2 and S6 Fig). Previous studies have shown that human and rodent *Retn* are expressed in different places and appear to have differing roles in diabetes [6,7,8]. The more rapid evolution of rodent *Retn* sequences imply that rodent resistin, rather than human resistin, changed its function, thus the role of resistin in maintaining blood glucose levels and diabetes [3] may be a rodent-specific trait. The ancestral role for resistin therefore is likely among its other characterized functions in the cardiovascular system [8,13,14] or in cancer [9].

## Conclusions

Our characterization of *Retn* and *Retnl* genes in diverse vertebrates allowed us to develop a model for the evolution of this gene family (Fig 5). *Retn* is located at the locus of origin for the duplicated genes, while the *Retnl* gene was transposed to a new genomic location during, or soon after, duplication. Since *Retn* is located at the locus or origin, it likely retained the ancestral expression pattern. This gene has also been maintained as a single copy gene in almost all mammals examined, suggesting that the function of this gene has not changed. In contrast, *Retnl* transposed to a new genomic location, displays evidence of accelerated sequence evolution after duplication, and has been retained in variable copy number on different mammalian lineages. The changes in genomic location, rate of sequence evolution, and copy number suggest that *Retnl* has evolved new, and possibly lineage-specific, functions. Our analyses also suggest that rodent *Retn* evolved more rapidly than *Retn* genes in other mammals, thus the difference in function of *Retn* in diabetes between mouse and humans likely represents the gain of a new function in rodents, and that rodents will not be a good model for understanding the function of the human *RETN* and *RETNL* genes.

## Supporting Information

S1 Fig Coding sequences of intact *Retn* and *Retnl* genes. (DOCX)

S2 Fig Duplication of exon 2 in *Retn2* from the Chinese alligator. (PDF)

S3 Fig Processed *Retn* pseudogenes in the camel and alpaca. (PDF)

S4 Fig Inactivation of ruminant artiodactyl *Retnl* by insertion of a repetitive DNA element. (PDF)

S5 Fig Alignment of processed *Retnl* pseudogenes from primates. (PDF)

S6 Fig Phylogeny of *Retn* and *Retnl* coding sequences generated by PhyML. (PDF)

S7 Fig Phylogenetic relationship of rodent *Retnl* genes. (PDF)

S8 Fig Genomic organization of genes near rodent *Retnl* genes. (PDF)

S1 Table Genomic locations of *Retn* and *Retnl* genes in vertebrate genomes. (PDF)
