# Supplementary material for: Evolution of the Vertebrate Resistin Gene Family
Source: PLoS One. 2015 Jun 15;10(6):e0130188. doi: 10.1371/journal.pone.0130188 (PMC4467842; doi:10.1371/journal.pone.0130188)
Supplement: S1 Fig — (DOCX) [file pone.0130188.s001.docx]

>Human_RETN_ENSG00000104918

ATGAAAGCTCTCTGTCTCCTCCTCCTCCCTGTCCTGGGGCTGTTGGTGTCTAGCAAGACCCTGTGCTCCATGGAAGAAGCCATCAATGAGAGGATCCAGGAGGTCGCCGGCTCCCTAATATTTAGGGCAATAAGCAGCATTGGCCTGGAGTGCCAGAGCGTCACCTCCAGGGGGGACCTGGCTACTTGCCCCCGAGGCTTCGCCGTCACCGGCTGCACTTGTGGCTCCGCCTGTGGCTCGTGGGATGTGCGCGCCGAGACCACATGTCACTGCCAGTGCGCGGGCATGGACTGGACCGGAGCGCGCTGCTGTCGTGTGCAGCCCTGA

>Human_RETNL_NSG00000163515

ATGGGGCCGTCCTCTTGCCTCCTTCTCATCCTAATCCCCCTTCTCCAGCTGATCAACCCGGGGAGTACTCAGTGTTCCTTAGACTCCGTTATGGATAAGAAGATCAAGGATGTTCTCAACAGTCTAGAGTACAGTCCCTCTCCTATAAGCAAGAAGCTCTCGTGTGCTAGTGTCAAAAGCCAAGGCAGACCGTCCTCCTGCCCTGCTGGGATGGCTGTCACTGGCTGTGCTTGTGGCTATGGCTGTGGTTCGTGGGATGTTCAGCTGGAAACCACCTGCCACTGCCAGTGCAGTGTGGTGGACTGGACCACTGCCCGCTGCTGCCACCTGACCTGA

>Pygmy_chimpanzee_Retn_XM_003810015.2

ATGAAAGCTCTCTGTCTCCTCCTCCTCCCTGTCCTGGGGCTGTTGGTGTCTAGCAAGACCCTGTGCTCCATGGAAGAAGCCATCAATGAGAGGATCCTGGAGGTCGCCGGCTCCCTAATATTTAGGGCAATAAGGAGCATTGGCCTGGAGTGCCAGAGCGTCACCTCCAGGGGGGACCTGGCTACTTGCCCCCGAGGCTTCGCCGTCACCGGCTGCACTTGTGGCTCCGCCTGTGGCTCGTGGGATGTGCGCGCCGAGACCACATGTCACTGCCAGTGCGCGGGCATGGACTGGACCGGAGCGCGCTGCTGTCGTGTGCAGCCCTGA

>Pygmy_chimpanzee_Retnl_XM_003825063.2

ATGGGGCCGTCCTCTTGCCTTCTTCTCATCCTGATCCCCCTTCTCCAGCTGATCAACCCAGGGAGTACTCAGTGTTCCTTAGACTCTGTCATGGATAAGAAGATCAAGGATGTTCTCAACAGTCTAGAGTACAGTCCCTCTCCTATAAGCAAGAAGCTCTCGTGTGCTAGTGTCAAAAGCCAAGGCAGACTGTCCTCCTGCCCTGCTGGGATGGCTGTCACTGGCTGTGCTTGTGGCTATGGCTGTGGTTCGTGGGATGTTCAGCTGGAAACCACCTGCCACTGCCAGTGCAGTGTGGTGGACTGGACCACTGCCCACTGCTGCCACCTGACCTGA

>Chimpanzee_Retnl_ENSPTRG00000015195

ATGGGGCCGTCCTCTTGCCTCCTTCTCATCCTAATCCCCCTTCTCCAGCTGATCAACCCGGGGAGTACTCAGTGTTCCTTAGACTCCGTTATGGATAAGAAGATCAAGGATGTTCTCAACAGTCTAGAGTACAGTCCCTCTCCTATAAGCAAGAAGCTCTCGTGTGCTAGTGTCAAAAGCCAAGGCAGACTGTCCTCCTGCCCTGCTGGGATGGCTGTCACTGGCTGTGCTTGTGGCTATGGCTGTGGTTTGTGGGATGTTCAGCTGGAAACCACCTGCCACTGCCAGTGCAGTGTGGTGGACTGGACCACTGCCCGCTGCTGCCACCTGACCTGA

>Gorilla_Retn_ENSGGOG00000009545

ATGAAAGCTCTCTGTCTCCTCCTCCTCCCTGTGCTGGGGCTGTTGGTGTCTAGCAAGACCCTGTGCTCCATGGAAGAAGCCATCAATGAGAGGATCCAGGAGATCGCCAGCTCCCTAATATTTAGGGCAATAAGGAGCATTGGCCTGGAGTGCCAGAGCGTCACCTCCAGGGGGGACCTGGCTACTTGCCCCCGAGGCTTCGCCGTCACCGGCTGCACTTGTGGCTCCGCCTGTGGCTCGTGGGATGTGCGCGCCGAGACCACATGTCACTGCCAGTGCGCGGGCATGGACTGGACCGGAGCGCGCTGCTGTCGTGTGCAGCCCTGA

>Gorilla_Retnl1_ENSGGOG00000009854

ATGGGGCCGTCCTCTTGCCTCCTTCTCATCCTAATCCCCCTTCTCCAGCTGATCAACCCAGGGAGTACTCAGTGTTCCTTAGACTCCGTTATGGATAGGAAGATCAAGGATGTTCTCAACAGTCTAGAATACAGTCCCTCTCCTATAAGCAAGAAGCTCTCATGTGCTAGTGTCAAAAGCCAAGGCAGACTGTCCTCCTGCCCTGCTGGGATGGCTGTCACTGGCTGTGCTTGTGGCTATGGCTGTGGTTCGTGGGATGTTCAGCTGGAAACCACCTGCCACTGCCAGTGCAGTGTGGTGGACTGGACCACTACCCACTGCTGCCACCTGACCTGA

>Gorilla_Retnl2_ENSGGOG00000022847

ATGGGGCCGTCCTCTTGCCTCCTTCTCATCCTAATCCCCCTTCTCCAGCTGATCAACCCAGGGAGTACTCAGTGTTCCTTAGACTCCGTTATGGATAGGAAGATCAAGGATGTTCTCAACAGTCTAGAATACAGTCCCTCTCCTATAAGCAAGAAGCTCTCATGTGCTAGTGTCAAAAGCCAAGGCAGACTGTCCTCCTGCCCTGCTGGGATGGCTGTCACTGGCTGTGCTTGTGGCTATGGCTGTGGTTCGTGGGATGTTCAGCTGGAAACCACCTGCCACTGCCAGTGCAGTGTGGTGGACTGGACCACTACCCACTGCTGCCACCTGACCTGA

>Orangutan_Retn_ENSPPYG00000009481

ATGAAAGCTGTCTGTCTCCTCCTTCTCCCTGTCCTGGGGCTGCTGGTGTCTAGCGAGAGCCTGTGCTCCTTGGAAGAAGCCATCAACGAGAAGATCCAGGAGGGCGCCAGCTCTCTAATATTTAGGGCAATAAGGAGCATTGACCTGGAGTGCCAGAGCGTCACCTCCAGGGGGGACCTGGCTACTTGCCCCCGAGGCTTCGCCGTCACCGGCTGCACTTGTGGCTCCGCCTGTGGCTCGTGGGATGTGCGCGCCGAGACCACATGTCACTGCCAGTGCGCGGGCATGGACTGGACCGGAGCGCGCTGCTGTCGTGTGCAGCCCTGA

>Gibbon_Retn_ENSNLEG00000001150

ATGAAAGCTCTCTGTCTCCTCCTCCTCCCTGTCCTGGGGCTGCTGGTGTCTAGCAAGACCCTGTGCTCCATGGAAGAAGCCATCAATGAGAAGATCCAGGAGGGCGCCAGCTCCCTAATATTTAGGGCAATAGGGAGCATTGGCCTGGAGTGCCAGAGCGTCACCTCCAGGGGGGACCTGGCTACTTGCCCCCGAGGCTTCGCCGTCACCGGCTGCACTTGTGGCTCTGCCTGTGGCTCGTGGGATGTGCGCGCCGAGACCACATGTCACTGCCAGTGCGCGGGCATGGATTGGACCGGAGCGCGCTGCTGTCGTGTGCAGCCCTGA

>Macaque_Retn_NM_001243087.1

ATGAAAGCCCTCTGTCTCCTCCTCCTGCCTGTCCTGGGGCTGCTGGTGTCTAGCCAGACCCTGTGCTCCATGGAAGAAGCCGTCAATGAGAAGATTCAGGAGGGCGCCAGCTCCTTAGTATTTAGGGCAATAAGGAGCATTGGCCTGGAGTGCCAGAGCGTCACCTCCAGGGGGGACCTGGCTACTTGCCCCCGAGGCTTCGCCGTCACCAGTTGCACTTGTGGCTCCGCCTGTGGCTCGTGGGATGTGCGCGCCGAGACCACATGTCACTGCCAGTGCGCGGGCATGGACTGGACCGGAGCGCGCTGCTGTCGTCTGCAGCCCTGA

>Macaque_Retnl_ENSMMUG00000016534

ATGGGGCCTTCCTCTTGCCACCTTCTCATCCTCATCCCCCTTCTCCAGCTGGTCATCCCAGGGAGTACTCAGTGTTCCTTAGACTCCGTTATGGATAAGAAGATCAAGGATATTCTCAACCGTCTAGAGTACAGTCCCTCTCCCGTAAGCAAGAAGCTCTTGTGTACTAGTGTCAAGAGCCAAGGCAGGCTGGCCTCTTGCCCTGCTGGGATGAGTGTCACTGGCTGTGCTTGTGGCTATGGCTGTGGTTCGTGGGATGTTCAGGGGGGAACCACCTGCCACTGCCAGTGCAGTGTGATGGACTGGACCACCGCCCGCTGCTGCTACCTGGCCTGA

>Olive_baboon_Retn_ENSPANG00000003097

ATGAAAGCTCTCTGTCTCCTCCTCCTGCCTGTCCTGGGGCTGCTGGTGTCTAGCCAGACCCTGTGCTCCATGGAAGAAGCCGTCAATGAGAAGATTCAGGAGGGCGCCAGCTCCCTAGTATTTAGGGCAATAAGGAGCATTGGCCTGGAGTGCCAGAGCGTCACCTCCAGGGGGGACCTGGCTACTTGCCCCCGAGGCTTCGCCGTCACCAGTTGCACTTGTGGCTCCGCCTGTGGCTCGTGGGATGTGCGCGCCGAGACCACATGTCACTGCCAGTGCGCGGGCATGGACTGGACCGGAGCGCGCTGCTGTCGTCTGCAGCCCTGA

>Olive_baboon_Retnl_ENSPANG00000020527

ATGGGGCCTTCCTCTTGCCACCTTCTCATCCTCATCCCCCTTCTCCAGCTGATCATTCCAGGGAGTACTCAGTGTTCCTTAGACTCCGTTATGGATAAGAAGATCAAGGATATTCTCAACCGTCTAGAGTACAGTCCCTCTCCCGTAAGCAAGAAGCTCTTGTGTACTAGTGTCAAGAGCCAAGGCAGGCTGGCCTCTTGCCCTGCTGGGATGAGTGTCACTGGCTGTGCTTGTGGCTATGGCTGTGGTTCGTGGGATGTTCAGGGGGGAACCACCTGCCACTGCCAGTGCAGTGTGATGGACTGGACCACTGCCCGCTGCTGCTACCTGGCCTGA

>Crab_eating_macaque_Retnl_XM_005548219.1

ATGGGGCCTTCCTCTTGCCACCTTCTCATCCTCATCCCCCTTCTCCAGCTGATCATCCCAGGGAGTACTCAGTGTTCCTTAGACTCCGTTATGGATAAGAAGATCAAGGATATTCTCAACCGTCTAGAGTACAGTCCCTCTCCCGTAAGCAAGAAGCTCTTGTGTACTAGTGTCAAGAGCCAAGGCAGGCTGGCCTCTTGCCCTGCTGGGATGAGTGTCACTGGCTGTGCTTGTGGCTATGGCTGTGGTTCGTGGGATGTTCAGGGGGGAACCACCTGCCACTGCCAGTGCAGTGTGATGGACTGGACCACTGCCCGCTGCTGCTACCTGGCCTGA

>Hamadryas_baboon_Retn

ATGAAAGCTCTCTGTCTCCTCCTCCTGCCTGTCCTGGGGCTGCTGGTGTCTAGCCAGACCCTGTGCTCCATGGAAGAAGCCGTCAATGAGAAGATTCAGGAGGGCGCCAGCTCCCTAGTATTTAGGGCAATAAGGAGCATTGGCCTGGAGTGCCAGAGCGTCACCTCCAGGGGGGACCTGGCTACTTGCCCCCGAGGCTTCGCCGTCACCAGTTGCACTTGTGGCTCCGCCTGTGGCTCGTGGGATGTGCGCGCCGAGACCACATGTCACTGCCAGTGCGCGGGCATGGACTGGACCGGAGCGCGCTGCTGTCGTCTGCAGCCCTGA

>Hamadryas_baboon_Retnln

ATGGGGCCTTCCTCTTGCCACCTTCTCATCCTCATCCCCCTTCTCCAGCTGATCATTCCAGGGAGTACTCAGTGTTCCTTAGACTCCGTTATGGATAAGAAGATCAAGGATATTCTCAACCGTCTAGAGTACAGTCCCTCTCCCGTAAGCAAGAAGCTCTTGTGTACTAGTGTCAAGAGCCAAGGCAGGCTGGCCTCTTGCCCTGCTGGGATGAGTGTCACTGGCTGTGCTTGTGGCTATGGCTGTGGTTCGTGGGATGTTCAGGGGGGAACCACCTGCCACTGCCAGTGCAGTGTGATGGACTGGACCACTGCCCGCTGCTGCTACCTGGCCTGA

>Vervet_monkey_Retn_ENSCSAG00000008325

ATGAAAGCTCTCTGTCTCCTCCTCCTCCCTGTCCTCGGACTGCTGGTGTCTAGCCAGACCCTGTGCTCCGTGGAAGAAGCCATCAATGAGAAGATTCAGGAGGGCGCCAGCTCCCTAGTATTTAGGGCAATAAGGAGCATTGGCCTGGAGTGCCAGAGCGTCACCTCCAGGGGGGACCTGGCTACTTGCCCCCGAGGCTTCGCCGTCACCAGTTGCACTTGTGGCTCCGCCTGTGGCTCGTGGGATGTGCGCGCCGAGACCACATGTCACTGCCAGTGCGCGGGCATGGACTGGACCGGAGCGCGCTGCTGTCGTCTGCAGCCCTGA

>Vervet_monkey_Retnl_ENSCSAG00000006391

ATGGGGCCTTCCTCTTGCCACCTTCTCATCCTCATCCCCCTTCTCCAGCTGATCATCCCAGGGAGTACTCAGTGTTCCTTAGACTCCGTTATGGATAAGAAGATCAAGGATGTTCTCAACCGTCTAGAGTACAGTCCCTTTCCCGTAAGCAAGAAGCTCTCGTGTACTAGTGTCAAGAGCCAAGGCAGGCTGGCCTCTTGCCCTGCTGGGATGAGTGTCACTGGCTGTGCTTGTGGCTATGGCTGTGGTTCGTGGGATGTTCAGGGGGGAACCACCTGCCACTGCCAGTGCAGTGTGATGGACTGGACCACTGCCCGCTGCTGCTACCTGGCGTGA

>Marmoset_Retn_XM_002761674.2

ATGAAAGCTCTCTGTCTCCTCCTCCTCCCTGTCCTGGGGCTTCTGGTGTCTAGCAAGAAGCTGTGCTCCTTGGATGAAGCCATGGATGAGATGATCCAGGAGGGCACCAGCTCCCTAATATTTAAGGCAATAACGAACATTGGCCTGGAGTGTCAGAGCGTCACCTCCAGGGGGGACCTGGCTACTTGCCCCCAAAGCTTCGCCGTTACCAGCTGCACATGTGGTTCTGCCTGTGGCTCGTGGGATGTGCGCGCCGAGACCACATGCCACTGCCAGTGCGCGGGCATGGACTGGACCGGAGCGCGCTGCTGTCGTCTGCGGTTCTGA

>Squirrel_monkey_Retn_XM_003938888.1

ATGAAAGCTCTCTGTCTCCTCCTCCTCCCTGTCCTGGGGCTTCTGGTGTCTAGCAAGAAGCTGTGCTCCTTGGATGAAGCCATTGATGAGATGATCCAGGAGGGCACCAGCTCCCTAATATTTAAGGCAGTAACGAGCATTCGCCTGGAGTGCCAGAGCGTCACCTCCAGGGGGGACCTGGCTACTTGCCCCCAAAGCTTCGCCGTTACCAGCTGCACATGTGGTTCCGCCTGTGGCTCGTGGGACGTGCGCGCCGAGACCACATGCCACTGCCAGTGCGCGGGCATGGACTGGACCGGAGCGCGCTGCTGTCGTCTGCGGTTCTGA

>Squirrel_monkey_Retnl_XM_003939399.1

ATGGGGCCTTCCTTTTGCCTGCTGCTCATCCTCATCCCCGTTCTCCAGCTAATGACCCTAGGGAGTACTCAGTGTTCTTTAGACTCCGTTATGGATAAGAAGATCAAGGATGTTCTCAGCAGTCTAGAGTACAGTCCCTCTCCCACAACCAAGAAGCTCTCGTGTATTAGTGTCAACAGCCGAGGCAGGCTGTCCTCCTGCCCTGCTGGGATGGCTGTCACTGGCTGTGCTTGTGGCTATGGCTGTGGTTCGTGGGATGTTCAGCTGGAAACCACCTGCCATTGCCAGTGCAGTGTGGTGGACTGGACCACTGCCCGCTGCTGCCACCTGGCCTGA

>Mouse_lemur_Retn_ENSMICG00000008250

ATGAAGGCTCTCTCCTTCCTCCTCCTCCCCGTCCTGGGGCTGCTGGTGTCCAGCAAGACACTGTGTCCGGTGGATGAAGCCATCGATGCTAAGATCCAGGAGAGCACCAGCTCGCTAATTTTGGGGGCATTAGGCAACATTGTCCTGAACTGCCAGACTGTCACCTCCAGGGGAGACCTGGCCACTTGTCCCGCAGGCTTCGCCATCACCGGCTGCACGTGTGGCTCCGCCTGCGGCTCGTGGGACGTGCGCGCCGAGACCACATGCCACTGCCAGTGCGCGGGCATGGACTGGAACGGAGCGCGCTGCTGTCGCCTGCAGGTCACCGGCGCCTGA

>Mouse_lemur_Retnl_ENSMICG00000000243

ATGAAGCCTGGCACCTGCCCCCTTTTCATCCTCATCCCCCTTCTCCAGCTGGTTATCCCAGGGAGTCCTGAGTGTTCCTTAGACACCATTGTGGAAAATAAGATCAAGGACATTCTCAACGGTCTAGAGTATAATCCCTCTGCCACAACAAAGCAGCTCGTGTGCACCAGCATCACAAACTCAGGCAAACTGTCCTCCTGCCCTGCAGGGATGTCTGTCACCGGCTGTGCTTGTGGCTACGCCTGTGGTTCCTGGGATGTCCGGCAGGAAAAGACGTGCCACTGCCAGTGCAGTGTGGTGGACTGGACCACTGCCCGCTGCTGCCGCCTGGCCTGA

>Bushbaby_Retn_XM_003803420.1

ATGAAGGCGCTCTCTCTCGTCCTTCTCCCTGTCCTGGGGCTGCTGGTACCCAGCCAGACACTGTGTCCCATGGATGAAGCCGTCAATAAGAAGCTCCAGGAGCACACCAGCTCCCTAATTTCTGGGTCATTAAGCAAGATGAGCCTGGACTGCCAAACTGTAACCTCCAGGGGGAACCTGGCCACTTGCCCCGCAGGCTTCACCATCAGCAGCTGCACGTGCGGCTCCGCCTGTGGTTCATGGGACGTGCGCGCCGAGACCACTTGCCATTGCCAGTGCGCAGGCATGGACTGGACCGGAGCGCGTTGCTGCCGCCTGCGTGCCACCGCAGCCTGA

>Bushbaby_Retnl_ENSOGAG00000013671

ATGAAGCCGACCATTTGCCTTCTGCTCATCCTCATCCCCCTTCTCCAGCTGATTATCCCAGGAAGTACTGAGTATTCCTTAGAAGCCACTGTGGAAAAAAAGATCAAGGAATTTCTCAACAGTCCAGAGTATAAGCCCCCTGCCGTGGCAATGAAGATCTCATGCACCAGTGTCAAACAATCGGGCAAATTTGCCTCCTGCCCTCATGGGATGTCTGTCACTGGCTGTGCTTGTGGCTATGGCTGTGGTTCATGGGATATCCGGCAGGAAACCACGTGCCACTGCCAGTGCCCTGGGATGGACTGGACCGCGGCCCGCTGCTGCCAGGTGGCCTGA

>Tree_shrew_Retn_XM_006170219.1

ATGAAGGTTCTCCTCCTGCTCCTCCTCCCCGTCCTTGGGACGCTGGTGTCCAGCAGCAAGCCGCAGTGTACCATCCAGGACGTCATTAACCAGAAGATCGAGGAGGACTTCAGCTCCCTGATTTTACAGGCAATAGGTAATATAGGCATGAACTGCCAAACCGTCACCTCCAGGGGGGCACTGGCCACCTGCCCCTCAGGATTCTCCGTCACCGCCTGCACGTGCGGCTCCGCCTGCGGCTCGGGGGACGTGCGCGCCGAGACCACGTGCCACTGCCAGTGCGCGGGCATGGACTGGACCGGAGCGCGCTGCTGTCGCCTGCAGGGCCGCGCCTGA

>Tree_shrew_Retnl_XM_006164208.1

ATGAAGCCTACCTTTGTCCTCATTCTCATCCTCATCTTCCTTGTCCAGCTGATGACCTCAGAGAGTTCTCAGTGTTCCTTAGACTCCATGGTAGATAAAAAGATCAAGGAAGCTCTCAACAGTCTAGAGTTCAATCCCTCTGTTCTGACCAAGTTCTCATGTACCAGTATCACCAGCTCAGGCAGACTGGCCTCCTGCCCTGCTGGAATGTCTGTCACCGGTTGTGCTTGTGGCTATGCCTGTGGTTCATGGGATGTCCAGAGTGAAACCACATGTCACTGCCAGTGCAGCGTGGTGGACTGGGCCACTGCCCGCTGCTGCCACCTGGTCTGA

>Mouse_Retn_ENSMUST00000169234

ATGAAGAACCTTTCATTTCCCCTCCTTTTCCTTTTCTTCCTTGTCCCTGAACTGCTGGGCTCCAGCATGCCACTGTGTCCCATCGATGAAGCCATCGACAAGAAGATCAAACAAGACTTCAACTCCCTGTTTCCAAATGCAATAAAGAACATTGGCTTAAATTGCTGGACAGTCTCCTCCAGAGGGAAGTTGGCCTCCTGCCCAGAAGGCACAGCAGTCTTGAGCTGCTCCTGTGGCTCTGCCTGTGGCTCGTGGGACATTCGTGAAGAAAAAGTGTGTCACTGCCAGTGTGCAAGGATAGACTGGACAGCAGCCCGCTGCTGTAAGCTGCAGGTCGCTTCCTGA

>Mouse_Retnla_ENSMUSG00000061100

ATGAAGACTACAACTTGTTCCCTTCTCATCTGCATCTCCCTGCTCCAGCTGATGGTCCCAGTGAATACTGATGAGACCATAGAGATTATCGTGGAGAATAAGGTCAAGGAACTTCTTGCCAATCCAGCTAACTATCCCTCCACTGTAACGAAGACTCTCTCTTGCACTAGTGTCAAGACTATGAACAGATGGGCCTCCTGCCCTGCTGGGATGACTGCTACTGGGTGTGCTTGTGGCTTTGCCTGTGGATCTTGGGAGATCCAGAGTGGAGATACTTGCAACTGCCTGTGCTTACTCGTTGACTGGACCACTGCCCGCTGCTGCCAACTGTCCTAA

>Mouse_Retnlb_ENSMUSG00000022650

ATGAAGCCTACACTGTGTTTCCTTTTCATCCTCGTCTCCCTTCTCCCACTGATAGTCCCAGGGAACGCGCAATGCTCCTTTGAGTCTTTGGTGGATCAAAGGATCAAGGAAGCTCTCAGTCGTCAAGAGCCTAAGACGATCTCCTGCACTAGTGTCACGTCTTCTGGCAGACTGGCCTCCTGTCCTGCTGGGATGGTTGTCACTGGATGTGCTTGTGGCTATGGCTGTGGATCGTGGGATATCCGGAATGGAAATACTTGCCACTGCCAGTGCTCAGTCATGGACTGGGCCTCTGCCCGCTGCTGCCGAATGGCTTAA

>Mouse_Retnlg_ENSMUSG00000022651

ATGAAGACTACAACTTGTTCCCTTCTCATCTGCATCTCCCTTCTCCAGCTGATGGTCCCAGTGAATACTGAGGGGACCTTAGAATCTATTGTGGAGAAAAAGGTCAAGGAACTTCTTGCCAATCGAGATGACTGTCCCTCCACTGTAACAAAGACTTTCTCCTGTACTAGTATCACGGCTTCAGGCAGACTGGCCTCCTGTCCTTCTGGAATGACTGTCACTGGTTGTGCTTGTGGCTATGGCTGTGGATCTTGGGATATCCGGGATGGAAATACTTGCCACTGTCAGTGCTCAACAATGGACTGGGCCACCGCCCGTTGCTGCCAACTGGCCTAA

>Rat_Retn_ENSRNOT00000001325

ATGAAGAACCTTTCATTTCTCCTCCTTTTCCTTTTCTTCCTTGTCCTGGGGCTGCTGGGCCCCAGCATGTCACTGTGTCCCATGGATGAAGCCATCAGCAAGAAGATCAATCAAGACTTCAGCTCCCTACTGCCAGCTGCAATGAAGAACACTGTCCTACATTGCTGGTCAGTCTCCTCCAGAGGGAGGCTGGCCTCCTGCCCAGAAGGCACAACCGTCACTAGCTGCTCCTGTGGCTCTGGCTGTGGCTCATGGGACGTCCGTGAGGATACAATGTGTCACTGCCAGTGCGGAAGCATAGACTGGACAGCGGCCCGCTGCTGTACCCTGCGGGTTGGTTCCTGA

>Rat_Retnla_ENSRNOG00000001955

ATGAAGACTGCAACCTGTTCCCTTCTCATCTGCGTCTTCCTTCTCCAGCTGATGGTCCCAGTGAATACTGATGGAACCTTAGACATTATTGGGAAGAAAAAGGTCAAGGAACTTCTAGCCCATCAAGATAACTATCCCTCTGCTGTAAGGAAGACCCTCTCATGCACTAATGTCAAGTCTATGAGCAAATGGGCCTCCTGCCCTGCTGGGATGACTGCTACTGGTTGTTCTTGTGGCTTTGCCTGTGGATCTTGGGAAATCCAGAATGAAAATATTTGCAACTGCCTGTGCTTAATCGTTGACTGGGCCTATGCCCGCTGCTGCCAACTGTCCTAA

>Rat_Retnlb_ENSRNOG00000032187

ATGAAGCCTACACTGTGTTTCCTTTTCATCCTCATCTTCCTTCTCTCGCTGATGGTCCCAGGGAACACGCAGTGCTCCTTTGAGTCTTTGGTGGATCAAAAGATCAAGAAAGCACTCAGTCAACAAAAGACTAAGAAGCTCTCCTGCACTAGTATCACGGCTGCTGGCAGACTGGCTTCCTGTCCTGTTGGGATGGTTGTCACTGGTTGTGCTTGTGGCTATGCCTGTGGATCTTGGGATATCCGGGATGGAACTACTTGCCACTGCCAGTGTGCAGTCATGGATTGGGTCACTGCCCGCTGCTGCCAAATGGTTTAA

>Rat_Retnlg_ENSRNOG00000001943

ATGAAGACTGCAATCTGTTCCCTTCTCATCTGCATCTTTCTTCTCCAGCTGATGGTCCCAGTGAATACTGATGGAACCTTAGAGTCTATTGTGGAGCAAAAGGTCAAGGAACTTCTTGCCCATCGAGATAACTGTCCCTCCACTGTAACGAAGACTCTCTCCTGCACTAGTGTCAAGGCTACAGGCAGATTGGCCTCCTGTCCTCCTGGAATGGCTGTCACTGGATGTGCTTGTGGCTATGCCTGTGGATCTTGGGATATCCGGGATGGAACTACTTGCCACTGCCAGTGCGCAGTCATGGATTGGGCCACTGCCCGCTGCTGCCAACTGTCCTAA

>Chinese_hamster_Retn_XM_003507749.2

ATGAAACACCTCTCACTTCTCTTCCTCCTCTTCCCTGTCCCGGGCTTTCTGGACCCCAGCAGGTCACTGTGTTCCATGGATGAAGCCACTGACAAGAAGATCAAGCAAAGCTTCAGATCCCTACTGTCAGAAGCAGTAATGAACATTGGCCTACGATGCCGGACAGTCTCTTCCAGAGGGGGGTTGGCTTCCTGTCCAGAAGGCTTAGCAGTCACCAGCTGCTCCTGTGGCTTTGCTTGCGGTTCGTGGGATGTTCGAGAGGGAACGACATGTCACTGCCAATGTGCAGGCATAGATTGGACAGCAGCCCGTTGCTGTGGCCTGGGGGTTGGTGCCTGA

>Chinese_hamster_Retnl1_XM_007640799.1

ATGAAGACTACAACTTGTTGCCTTCTCATCTCTATCTGCTTTCTCCAGCTGATGGTCCCAATGAATACTGAGAACACTTTAGACTCTTTGCTGGAGGAAAAAATCAAGAAACTTATCGGCTGTGGAGATGACTGTACCTCCACTGTGACTAAAACTCTCTCCTGCACTAGTATCCAGGCCTCAGGCAGACGAGCCTCCTGTCCACCCGGGACGGTTGTCACCGGTTGTGCTTGTGGCTATGGTTGTGGATCATGGGATATCCAGAATGGAAATACTTGCCACTGTCAGTGCTCAGTCATGGACTGGGCCACTGCCCGCTGCTGCCAATTGGCCTGA

>Chinese_hamster_Retnl2_XM_007640798.1

ATGAAGACAACAATTTGCTCTCTTCTCATCATCATCTCGCTTCTTCAACTGATGGTCCCAGTGAATACTGAGGAGACCCTAGACTCTGTAATGAAGAGCCTCAAGGAACTTGTCAGCTATCACGATGGCCGTCGCTCAACTATGACTACAACACTTTCCTGCACTAGTGTCAAGGCTATGGGCACCCTATCCTCCTGTCCTTCTGGGATGACTGTTACTGGTTGTTCTTGCGGCTTTGCTTGTGGATCTTGGAATGTCCAGAATGAAAACGTTTGCCAATGCCTGTGCCCAATCATAGACTGGACTATTGCCCGCTGCTGCAAACTGGCCTGA

>Chinese_hamster_Retnl3_XM_007640803.1

ATGAAGCCTACAATATGTTTCCTCCTCATCTTCGTCTCCCTTCTCCATTTGATGAGCTCTGTGAATACTCAGTCCTCCAAAGACTCTTGGCTGACTAGAAAGATCCAGGAAGCTCTCAATGGTAAAGGCCACCATTGGGAGCCTAAGAAGCTCTCCTGCATTAGTGTCAAAAATTCAGGCAGACGCGCTTCCTGTCCTGGGGGGATGGCTGTCACTGGTTGTGCTTGTGGCTATGGTTGTGGATCATGGGATATCCAGAATGGAAATACTTGCCACTGTCAGTGCTCAGTCATGGACTGGACCACCGCCCGCTGCTGCCGACTGGCCTGA

>Chinese_hamster_Retnl5_XM_007625129.1

ATGAAGACTGCAACTTGTTGCCTTCTTATCTTTATCTGCCTTCTCCAGCTGATGGTTCCAATGAATACTGTGAACTTAGACTCTCTGCTGGAGGAAAAAATCAAGGAATTTATCGGCTGTGGAGATGGCTGTCCCTCCACTGTGACTAAAACTCTCTCCTGCACTAGTATCAAGGCCTCAGGCAGACTAGCTTCCTGTCCTCCTGGGATGGCCGTCACTGGTTGTGCTTGTGGCTATGGTTGTGGATCATGGGATATCCAGAATGGAAATACTTGCCACTGTCAGTGCTCAGTCATGGACTGGGCCACTGCCCGCTGCTGCCAACTGGCTTGA

>Golden_hamster_Retn_XM_005085269.1

ATGAAAACCCTCTCACTTCTTCTCTTCCTCCTCGCCCCTGTCCCGGGCTTGCTGGGCTCCAGCAGATCACTGTGTTCCATGGATGAAGCCATCGACAAGAAGATCAAGCAAGGCTTCGGCTCCCTATTGTCAGAAGCAGTAGCGAACATTGGCTTGCAATGCCGGACAGTCTCTTCCAGAGGGGAGTTGGCCTCCTGTCCACAAGGCTTAGCAGTCACCAGCTGCTCCTGTGGCTTTGCCTGCGGCTCGTGGGATGTTCGAGAGGGAACGACATGTCACTGCCAGTGCGCGGGCATAGATTGGACAGCAGCCCGTTGCTGTGGCCTGGGGGTTGGTGCTTGA

>Golden_hamster_Retnl1_XM_005074772.1

ATGAAGCCTACAATATGTTTCCTCCTCATCTTCATCTCCCTTCTCCATCTGATGACCTCAGTGAACACTCAGTCCTCCAAAGACTCATCAGGGAATATAAAGATCCGGAAAGCTCTCAGTGGTAAAGGGCCTAAGAAACTCTCCTGCCTTAGTGTCCAAAAATCAGGCAGATGGGCTTCCTGTCCTGAGGGGATGGCTGTCACTGGTTGTGCTTGTGGCTATGGTTGTGGATCATGGGATGTCCAGAATGGAAATACTTGCCACTGCCAATGCTCGGTCATGGACTGGACCACCGCCCGCTGCTGCCAACTGGCCTGA

>Golden_hamster_Retnl2_XM_005074817.1

ATGAAGACTAAAACCTGCTCCCTTCTCATCATCATGTCCCTTCTTCAACTGATAGTCCCAGTGAATACGGAAGAGACCTTAGACTCTGTCATGAAGAAGCTCAAGGAAGCTCTCAATCGTCTAGAGCATCCTCCCTCCACTGTGACTAAGAGTCTCTCCTGTACTACTGTCAAAGCTTCGGGTACATTGGCTTCCTGCCCACCTGGTATGATTGCTACTGGTTGTTCTTGTGGCTTTGCTTGTGGATCTTGGAATGTCCAGAATGAAAATGTTTGCCACTGCCTGTGCCCAGTCATAGACTGGACTGTTGCCCGCTGCTGCCAACTGAGAAAATGA

>Golden_hamster_Retnl3_XM_005074818.1

ATGAAGACAACAATTTGCTCTCTTCTCATCATCGGCTCCCTTCTTCAACTGATGGTCCCAGTGAATACTCAGGACAACTTAGTCTATCTCCTGGAGAGGCTCAGGGATCTTCTCAACCAACAAGGCTACCGTCCCTCCACTGTGCCTAAGGCTCCCTCCTGCACTAGTGTCAAGGCTATGGGCAGACTAGCCTCCTGTCCTACTGGTATGATTGCTACTGGTTGCTCTTGTGGTTTTGCTTGTGGCTCTTGGAATATCCAGAATGAAAACATTTGCCACTGCCTGTGTCCAATCACAGACTGGACTGCTGCCCGCTGCTGTCTACTGTCCTGA

>Golden_hamster_Retnl4_XM_005074773.1

ATGAAGACTACAACTTGTTTCCTTCTCATCTTTATCGGCCTTCTCCAGCTGATGGTCCCAATGAATACTGTGAGCTTAGAGTCTCTACTGGAGGAAAAAATCAAGGAACTTACCAAATGTGGAGATGGCTGTCCCTCCCCTGTGACTAAGACTCTCTCCTGCACTAGTATCAAGGCCTCAGGCAGACTAGCCTCCTGTCCTTCTGGGATGGCTGTCACTGGTTGTGCCTGTGGCTATGGTTGTGGATCATGGGATATCCAGAATGGAAATACTTGCCACTGCCAGTGCTCAGTCATGGACTGGGCCACTGCACGCTGCTGCCAACTGGCCTGA

>Golden_hamster_Retnl5_XM_005074775.1

ATGAAGACAACAATTTGCTCTCTTCTCATCATCGGCTCCCTTCTTCAACTGATGGTCCCAGTGAATACTCAGGACAACTTAGCCTCCCTCCTAGGGAAGCTCAAGGAACTTCTCAAAAATCAAGATGGCTGTCCCTCCCCTGTGACTAAGACTCTCTCCTGCACTAGTATCAAGGCCTCAGGCAGACTAGCCTCCTGTCCTTCTGGGATGGCTGTCACTGGTTGTGCCTGTGGCTATGGTTGTGGATCATGGGATATCCAGAATGGAAATACTTGCCACTGCCAGTGCTCAGTCATGGACTGGGCCACTGCACGCTGCTGCCAACTGGCCTGA

>Kangaroo_rat_Retn_ENSDORG00000009639

ATGAAGGCTGTGTCTCTCCTCCTACTCCTCCTCTCTCTCCTGGGTCTGTTTGTGTCCAGCAAGTCTCTGTGTACCCTGGAAGAAGGCATCAATCAGAAGCTGAAGGATGGTGTCACATCTCTAATTTTAGAGGCGCTCAAGGACATTGGACTGGATTGTAAGACTGTGACATCCAGGGGGGACCTGGCTTCCTGCCCTGCAGGTCATGTTGTCACGGGATGCGCGTGTGGCATGGGCTGTGGCTCGTGGGATGTGCGTGCCGGGAACACGTGCCACTGTCAGTGCGGGGGCATGGACTGGACCGCAGCGCGCTGCTGCCACCTGAGGGGCACGGCTTGA

>Prairie_deer_mouse_Retn_XM_006970173.1

ATGGAAAACCTCTCACTCCTCCTCCTCTTCCTCCTCTTCCCTGTCCCGGGGTTGCTGGGCTCCAGCAGGGCACTGTGTTCGATGGATGAAGCCATGGACGAGAAGATGAAGCAAGGCTTCAGCTCCCTGTTGTCAGATGCGGTAAAGAACATTGGCATACAGTGCCGGACAGTCTCTTCCCGAGGGGAGTTGGCCTCCTGTCCAGAGGGCTTAGCAGTCACCAGCTGCTCCTGTGGTTCTGCCTGTGGCTCGTGGGACGTTCGAGAGGGAACAATATGTCACTGCCAGTGTGCAGGCATAGATTGGACCGCAGCCCGTTGCTGTACCCTGGGGGTTGGCGCCTGA

>Prairie_deer_mouse_Retnl1_XM_006996503.1

ATGAAGCCTACAATGTGTTTTCTTCTTATCTTCATCTCCCTCCTCCATCTGATGATCACAGTGAACACTCAGTCCTCTGTAGACTCTTCAGTGCATAAAAATAACCAGGACGATTTCCACGGTGTAGACCCCCATTGGGGGCCTTCGAAGCTCTTCTGCACTCGTGTCAAAAATCCAGGCAGATGGTCCTCCTGTCCTCCTGGGATGACTGTTACTGGCTGTTCTTGCGGCTCTGGCTGTGAATCTTGGGAGATCCAGAATGGAAATACTTGCCACTGCCAATGCTCAGCCATGGACTGGACCATCGGCCGCTGTTGCCGACCAGCTTGA

>Prairie_deer_mouse_Retnl2_XM_006996517.1

ATGAAGAGTGCAACCTGCTCACTTTTCATCATCATCTCCCTTCTTCAACTGATGATTCTAGTGAATACTGAAGAGACCTTAGACTCCATTATGAAGAGGCTCAAGGAAGCTCTCAGTCGCCTAGAGCATCCTCGCCCCACTCAGACTAAGAGTGTCTCCTGCACTACTGTCAAAGCTTCGGGGAAACTGGCTTCCTGTCCATCTGGGATGATTGCTACTGGATGTTCTTGTGGCTTTGCTTGTGGATCATGGAACATACAGAACGAAAACATTTGCCACTGCCTGTGTCCAATCATAGACTGGACTCTTGCCCGATGTTGCGATCTGAGGAAATGA

>Prairie_deer_mouse_Retnl3_XM_006996507.1

ATGAAGACTACAACCTGCTCCCTTCTCATCATCATCTCACTTCTCCAGCTGATGATCCCAGTGAACACTGAGGAGACATTAGACTCCATCATAAAGAAGCTCAAGGAAACTCTCAGCTATGGAGGATACCTTCCCTCGACTGTGAATAGGACTCCCTCTTGCACTAGTGTGAAGGCTGTAGGCAGACTAGCCTCCTGTCCTGCTGGGATGACTGCTACTGGATGTTCTTGTGGCTTTGCTTGTGGATCATGGAACATACAGAATGAAAACATTTGCCACTGCCTGTGTCTATTCGTAGACTGGACTGTTGCTCGCTGCTGCCAACTGAGGAAATGA

>Prairie_deer_mouse_Retnl4_XM_006996504.1

ATGAAGACTACAACCTGCTCGCTTCTCATCATCATCTCACTTCTCCAGCTGATGATCCCAGTGAACACTGAGGAGACCTTAGACTCTATCATGAAGAAGCTCAAGGAAGCTCTCAGGGATGGAGGTAACCGTCCCTCTACTGTGACCCATACATTCTCCTGCACTAGTGTGAAGGCTTTAGGCAACCTTGCCTCCTGTCCTGCTGGGATGATTGCTACTGGATGTTCTTGTGGCTTTGCTTGTGGATCATGGAATGTCCAGAATGAAAATGTTTGCCACTGCCTGTGTCCAATTATTGACTGGACTCTTGCCCGCTGCTGCCAACTGTCCTGA

>Prairie_deer_mouse_Retnl5_XM_006996508.1

ATGAAGACTACAACTTGCTCCCTTCTCATCATCATCTCCCTTCTCCAGCTGATGATCCCAGTGAATACTGAGACCTTAGATTCTCTCCTGGAGAAGAAGATCAAGGAACTGGGCTGTGGAGGTGAGTGTACCTCCACTGTGACGAGGACTCTCTCCTGCACTAGTGTGACAGCTAGAAGCAGACTAGCCTCCTGTCCTGCTGGGATGGCTGTCACTGGCTGTGCTTGCGGCTATGGCTGTGGATCATGGGATATCCAGAATGGAAATACTTGCCACTGTCAGTGTTCAGTCATAGACTGGACCACCGCCCGCTGCTGCCAACTGGCCTGA

>Prairie_deer_mouse_Retnl6_XM_006996509.1

ATGAAGACTACAATTTGCTCCCTTATCATCATCATCTCCATTCTTCACCTGATGATCCCAGTGAATACTGAGGAGACTTTAGATTCTCTCCTGGAGAAAAAGATCAAGAAACTTCTCAACTGTGGAGGTGAGTGTACCTCCACTGTGACTAGGACTCTCTCCTGCACTAGTGTGAACGCTAGAGGCAGACTAGCCTCCTGTCCTGCTGGGATGGCTGTCACTGGCTGTGCTTGTGGCTATGGCTGTGGATCATGGGATATCCGGAATGGAAATACTTGCCACTGTCAGTGTTCAGTCCTAGACTGGACTACCGCTCGCTGCTGCCAACTGGCCTGA

>Prairie_deer_mouse_Retnl7_XM_006989974.1

ATGAAGACTACAATCTGCTCCCTTATCATCATCATCTCCATTCTTCAACTGATGATCCCAGTGAATACTGAGGAGACCTTAGATTCTCTCCTGGAGAAAAAGATCAAGAAACTTCTCAACTGTGAGTGTACCTCCACTGTGACTAGGACTCTCTCCTGCACTAGTGTGAACGCTAGAGGCAGACTAGCCTCCTGTCCTGCTGGGATGGCTGTCACTGGCTGTGCTTGTGGCTATGGCTGTGGATCATGGGATATCCGGAATGGAAATACTTGCCACTGTCAGTGTTCAGTCCTAGACTGGACTACCGCTCGCTGCTGCCAACTGGCCTGA

>Prairie_deer_mouse_Retnl8_XM_006989975.1

ATGAAGACTACAACTTGCTCCCTTCTCATCATCATCTCACTTCTCCAGCTGATGATCCCAGTGAACACTGAGGAGACCTTAGACTCCATCATAAAGAAGCTCAAGGAAGCTCTCAGCGATGGAGGTAACCATCCCTCTACTGTGACCCATACACTCTCCTGCACTAGTGTGAAGGCTTCAGGCAACCTAGCCTCCTGTTCTGCTGGGATGATTGCTACTGGATGTTCTTGTGGCTTTGCCTGTGGATCATGGAACATCCAGAATGAAAATGTTTGCCACTGCCTGTGTCCAATTATTGACTGGACTCTTGCCCGCTGCTGCCAACTGTCCTGA

>Prairie_deer_mouse_Retnl9_XM_006989977.1

ATGAAGACTACAATCTGCTCCCTTATCATCATCATCTCCATTCTTCAACTGATGATCCCAGTGAATACTGAGGAGACCTTAGATTCTCTCCTGGAGAAAAAGATCAAGAAACTTCTCAACTGTGGAGGTGAGTGTACCTCTACTGTGACGAGGACTCTCTCCTGCACTAGTGTGAACGCTAGAGGCAGACTAGCCACCTGTCCTGCTGGGATGGCTGTCACTGGCTGTGCTTGCGGCTATGGCTGTGGATCCTGGGATATCCAGAATGGAAATACTTGCCACTGTCAGTGTTCAGTCCTAGACTGGACCACCGCTCGCTGCTGCAAACTGGCCTGA

>Prairie_vole_Retn_XM_005371737.1

ATGAAAAACCTCTCGCTTCTCCTCCTCTTCCTCCTCTTCCCTGTCCCGGGGTTGCTGGACTCCAGCAGGTCACTCTGTTCCATGGATAAAGCCATTGACAAGAAGATCAAGCAAAGCTTCAGCTCCCTATGGTCAGAAGCAGTAATCAGCGGTGGCATCCGATGCCGGACAGTCTCTTCCAGAGGGGACTTGGCCTCCTGCCCAGAAGGCTTAGCAGTCACCAGCTGCTCCTGTGGCTCTGCCTGTGGCTCGTGGGATGTTCGAGAGGGAACAACGTGTCACTGCCAGTGTGCAGGCATAGATTGGACAGCAGCCCGTTGCTGTGCCCTGGGGGTTGGTGCCTGA

>Prairie_vole_Retnl3_XM_005345041.1

ATGAAGACTGCAACTTGTTACCTTCTCGTCTGCATCTCCCTTCTGCAGCTGATGGTGCCAGTGAATACTGCGGAGACCTTAGACTCGCTCCTGGAGAAAAAAGTCAAGAAACTTCTCCACTGTGGAGATGGCTGTCCCTCCACTGTGACTCAGACTCTCTCCTGCACTAGTGTTAAAGCTATGGGCACCCTAGCCTCCTGTCCTGCTGGGATGGTTGCTACTGCTTGTTCTTGTGGCTTCGCTTGTGGATCTTGGAATGTACAGAATGAAAATGTTTGCCACTGCCTGTGTCCAGTCATAGACTGGACCGTAGCCCGCTGCTGCCAACTGGCCTGA

>Prairie_vole_Retnl5_XM_005372257.1

ATGAAGTCTACAATATGGTTCCTTCCCATCTTCATGTCTCTTCTCCATCTGATGACCCCAGTGCACACTCAGGCCTCCAGAGACTCTTCAGTGAATAAAAAGATCCCGGAAGCTCTCAATAGTAGAAAGCCTAAGAAGCTCTCCTGCACTAGTGTCAAAGCATCAGGCAGTTGGGCCTCCTGTCCTGCTGGGAAAGCTGTCACTGGTTGTTCATGCGGCTATGGTTGTGGATCGTGGGATGTCCAGAATGGAATTACTTGCCACTGCCAGTGCGCAGGCATGGACTGGACCGCCGCCCGCTGCTGCCAACTGGTTTGA

>Squirrel_Retn_ENSSTOT00000021041

ATGAGGGCTCTCTGCGTCCTTCTCCTCCCTGTCCTGGGGTTGCTGGTGTCCAGCAAGTCATCGTGTCCCATGGACGAAGCCGTCGATGAAAAGATCCAGGGGGGCATCAGCTCGCTAATTTTTGCAGCAATGCAGAACATCCGCCTGGACTGCAAGACCCTCACCTCCAGGGGGAACCTGGCTACCTGCCCTGCAGGCCACGCTGTCACCGCCTGCACGTGTGGCTCAGCCTGTGGCTCATGGGACGTGCGCGCCGAGACCACGTGCCACTGCCAGTGCGCGGGCATGGACTGGACCGGAGCGCGCTGCTGTCGTGTGCAGGCCGCCTGA

>Squirrel_Retnl_ENSSTOG00000024867

ATGAAGCATACTTCTTGCCTCCTCATCTTCATCTCCCTTCTTCAGCTGATGACTCCGGGGAGTTCTCAGTGTTCCTTAGACTCTGTAGTGAATAAAAGAATTCAGGAAGCTCTCAAAGGTACAGAGTATAAACCCTCCATCCCAACCAAGACATTCTCGTGTGTCAGCATCACAAGCTCAGGAAGACTGTCCTCCTGCCCCGCTGGTATGGTCGTCACTGGTTGTGCTTGTGGTAATGCCTGTGGTTCCTGGGATGTACGGGGAACAACCACATGTCACTGCCAGTGTTCTAAGATAGGCTGGACCACTGCCCGTTGCTGCCATCTGGCCTGA

>Naked_mole_rat_Retn_XM_004886644.1

ATGAAGGCTGTCTCCCTCCTCCTCCTCGCCCTGGGGCTGCTGGTGTCCCGGGGGTCACTGTGTTCCATGGACGAAGCCATCGATCAGAAGATCAAAGAGCAAACCAGCTCCCTAATTTTAGAGGCTCTCGAGAAGGCAGACCTGGACTGCAGAAGCGTCACCTCCAAGGGGGACCTGGCCACCTGTCCCGCAGGCTTCGCGGTCACCAGCTGCGCCTGTGGCATGGGCTGTGGCTCCTGGGACGTGCGCGCCCAGACCACGTGTCACTGCCAGTGCGCCCGCATGGACTGGACCGCAGTGCGCTGCTGCCGCCTGCAGATGGCCTCCTGA

>Guinea_pig_Retn_ENSCPOT00000010912

ATGAAGGCCGTGTTTCTCCTCCTCTTCCTCCCCATCCTGGGGCTGCTGGTGTCCAGTCAGTCACTGTGTCCGGTTGATGAAGCCATCAATCAGAAGATCCAGCATCAAGCCAAGTCTCTAATTTTAGGGTCTCTGAACGCTGTGCCACTAGACTGCAGAAGCGTCTCCTCCAGGGGGTCCCTGGCCACATGTCCCGCAGGCTTCGCGGTCACTGGCTGCGCGTGTGGCTCGGCTTGCGGCTCATGGGACGTGCGCGCTGAGACCACGTGCCACTGCCAGTGCGCGGGCATGGACTGGACCGCGGCGCGCTGCTGTCGTCTGCACGTGGCCTCCTGA

>Degu_Retn_XM_004644944.1

ATGAAGGCTGACTCCCTCCTCCTCCTCCTCTTCCTCCCCACCCTGGGGCTGCTGGTGTCCAGCGAGTCACTGTGTCCTGTGGATGAAGCCATCAATGAGAAGATCCAAAGCCAAGTCAGCTCCCTAAGTTTAGGGGCTGTGAAGAACATGGGCCTGGACTGCAGAAGCGTCTCCTCCAGGGGGACCCTGGCCACCTGTCCTGCAGGCTTCGCCGTCACGGGCTGCGCATGCGGCATGGGCTGTGGCTCATGGGACGTGCGCGCCGAGACCACGTGCCACTGCCAGTGCGCTGGCATGGACTGGACGGCGGCACGCTGCTGTCGCCTGCGGGTGGTCGCCTGA

>Egyptian_jerboa_Retnl_XM_004663972.1

ATGAAGCCTACGATTTGCTTCCTTCTTATTTTCATCTCCGTTCTGCAGCTGATGAGCCCAGGGAATGCTCAATGCTCCTTAGACTATGTTGTGGATACAAAGATCAAGGATGCTATCAGCAGAATAGGTGTGAAGAAGAATATAGCCTGCATCAGTGTGAAAAACTCAGGCTCCCTGGCCTCATGCCCTGCTGGGATGGTTGTCACTGGGTGTGCTTGTGGCTACGGCTGTGGCTCATGGGATGTCCAGAATGGGAACACGTGCCATTGCCAGTGCAGTGGGATGGACTGGACTACTGCCCGCTGCTGCCACCTGGTCTGA

>Chinchilla_Retn_XM_005413172.1

ATGAAGGCTGTCTCCCTCCTCCTCCTCTCCGCCTTGGGGCTGCTGGTGTCCAGTGAGTCACTGTGTCCTGTGGACGAAGCCATCAATCAGAAGATCCAAAGCCAAGCCAGCTCTCTCATTTTAGGGGATCTGAAGAACTTGGACCTGGACTGCAGGAGTGTCTCCTCCAGGGGCAATCTGGCCACCTGTCCCTCAGGCTTCGAGGTCACTGGCTGCGCGTGCGGCATGGCCTGCGGCTCGTGGGACGTGCGCGCGGGGACCACATGCCACTGCCAGTGCGCGGGCATGGACTGGACCGCGGCGCGCTGCTGTCGCCTGCGCGTGGCCGCCTGA

>Rabbit_Retn_NM_001199229.1

ATGAAGGCTCTCTCCCTCCTCCTCCTCCTCCTCCCTGCACTGGGGCTGCTGGTGTCCAGTGAGTCACTGTGTCCGGTGGATGATGCCATCAACGAGAAGATCAAGGACGGCACCAGCTCCCTGTTTCTTAGTGGAACGAGGAGCCTGAACCTGGAATGCCGCACTGTCACCTCCAGGGGGGCCCTGGCCACCTGTCCCGGAGGCTACTCCGTCACCAGCTGCACCTGTGGCTCCGCCTGCGGCTCGTGGGACGTGCGCGCGGAGACCACGTGCCACTGCCAGTGCGCGGGCATGGACTGGACCGGAGCGCGCTGCTGCCGCATCAGGACTAACGCCTGA

>Pika_Retn_ENSOPRG00000004676

ATGAAGGTTCTTGACTTCCTCCTCCTCCTCTTCCTCCCGGTCCTGGCAAGACTGGTGTGTGGCAGCAACCTGTGTCCTGTGGATGACGCCATCAACGAGAGACTGCAGAGTGTCGCCAGCTCCCTGTTCACGGCAGCGGTGTGCCTGGACCTGGACTGTCACACCGTCAAGTCTGGCGGCAACCTGGCCACCTGCCCCACAGGCTTTGGGGTCACCGGCTGCGCCTGTGGCTTCGGCTGCGGGTCCTGGGACGTGCGCTCCGAGAAGACGTGCCACTGCCAGTGCGCGGGTATGGACTGGACCGCGGCGCGCTGCTGCCGTCTGAAGAGCACCCCGAAGACCACCGCCTGA

>Cow_Retn_ENSBTAT00000006189

ATGAAGGCTCTCTCCTTCCTCTTCATCCCAGTCCTGGGGCTGCTGGTGTGTGGCCAGTCGCTGTGCCCCATAGATAAAGCCATCAGTGAGAAGATCCAGGAGGTCACCACCTCCCTAGTTCCTGGGGCAGTAAGGATCATTGGCCTGGACTGCCGGAGTGTCACCTCTAGGGGGTCCCTGGTCACCTGCCCTTCAGGCTTCGCCGTCACTGGCTGCACGTGTGGCTCCGCCTGTGGCTCGTGGGACGTACGTGCTGAGACCACGTGCCACTGCCAGTGCGCAGGCATGGACTGGACTGGAGCTCGCTGCTGCCGCCTGCATATCCAGTAG

>Yak_Retn_XM_005890748.1

ATGAAGGCTCTCTCCTTCCTCTTCATCCCAGTCCTGGGGCTCCTGGTGTGTGGCCAGTCGCTGTGCCCCATAGATAAAGCCATCAGTGAGAAGATCCAGGAGGTCACCACCTCCCTAGTTCCTGGGGCAGTAAGGATCATTGGCCTGGACTGCCGGAGTGTCACCTCTAGGGGGTCCCTGGTCACCTGCCCTTCAGGCTTCGCCGTCACTGGCTGCACGTGTGGCTCCGCCTGTGGCTCGTGGGACGTACGTGCTGAGACCACGTGCCACTGCCAGTGCGCAGGCATGGACTGGACTGGAGCTCGCTGCTGCCGCCTGCATATCCAGTAG

>River_buffalo_Retn_XM_006046759.1

ATGAAGGCTCTCTCCTTCCTCTTCATCCCAGTCCTGGGGCTGCTGGTGTGTGGCCAGTCGCTGTGCCCCATAGATAAAGCCATCAGTGAGAAGATCCAGGAGGTCACCACCTCCCTAGTTCCTGGGGCAGTAAGGATCATTGGCCTGGACTGCCGGAGTGTCACCTCCAGGGGGTCCCTGGTCACCTGCCCTTCAGGCTTCGCCGTCACTGGCTGCACGTGTGGCTCTGCCTGTGGCTCGTGGGACGTGCGTGCTGAGACCACGTGCCACTGCCAGTGCGCAGGCATGGACTGGACTGGAGCTCGCTGCTGCCGCCTGCGTATCCAGTAG

>Sheep_Retn_ENSOARG00000002291

ATGAAGGCTCTCTCCTTCCTCTTCATCCCAGTCCTGGGGCTGCTGGTGTGTGGCCAGTCACTGTGCCCCATAGATAAAGCCGTCAGTAAGAAGATCCAGGATGTCACCACCTCCCTACTTCCTGAGGCAGTAAGGAACATTGGCCTGGACTGCCAGAGTGTCACCTCCCGGGGGTCCCTGGTCACCTGCCCTTCAGGCTTCGCCGTCACTAGCTGCACGTGTGGCTCTGCCTGCGGCTCTGGGGCGGGGCGTGCTGAGACCACGTGCCACTGCCAGTGCGCAGGCATGGACTGGACTGGCGCTCGCTGCTGCCGCCTGCGGATCCAGTAG

>Goat_Retn_XM_005682406.1

ATGAAGGCTCTCTCCTTCCTCTTCATCCCAGTCCTGGGGCTGCTGGTATGTGGCCAGTCACTGTGCCCCATAGATAAAGCTGTCAGTAAGAAGATCCAGGATGTCACCACCTCCCTAGTTCCTGAGGCAGTAAGGAACATTGGCCTGGACTGCCAGAGTGTCACCTCCAGGGGGTCCCTGGTCACCTGCCCTTCAGGCTTCGCGGTCACTAGCTGCACGTGTGGCTCTGCCTGTGGCTCGTGGGACGTGCGTGCTGAGACCACGTGCCACTGCCAGTGCGCAGGCATGGACTGGACTGGCGCTCGCTGCTGCCGCCTGCGGATCCAGTAG

>Bottlenose_Dolphin_Retn_ENSTTRG00000001641

ATGAAGGCTCTCTCCCTTCTCCTCCTCCCAGTCCTGGGGCTGCTGGTGTGTGGCAAGTTGACGTGTCCCATGGATAAAGCCATCAGTGAGAAGATCCAGGAACTCACCAGCTCCCTAAATCCTGCGGCAATAAGGAACATTGGCCTGGACTGCCGCAGTGTCACCTCCAGGGGGCACCTGGTCACTTGCCCCGCAGGTTTCGCCGTCACCAGCTGCACCTGCGGCTCTGCCTGTGGCTCGTGGGACGTGCGCGCCGAGACCACGTGCCACTGTCAGTGCGCCGGCATGGACTGGACCGGAGCTCGCTGCTGTCGCCTGCGGGCCTAA

>Sperm_whale_Retn_XM_007107097.1

ATGAAGGCTCTCTCCCTCCTCCTCCTCCCAGTCCTGGGGCTGCTGGTGTGTGGCAAGTTGCTGTGTCCCATGGATAAAGCCATCAGTGAGAAGATCCAGGAACTCACCAGCTCCCTAATTCCTGAGGCAATAAGGAACATTGGCCTGGACTGCCAGAGTGTCACCTCCAGGGGGCACCTGGTCACTTGCCCCTCAGGTTTCACCGTCACCGGCTGCACCTGTGGCTCTGCCTGTGGCTCGTGGGACGTGCGCGCCGAGACCACGTGCCACTGCCAGTGCGCCGGCATGGACTGGACCGGAGCTCGCTGCTGTCGCCTGCGGGCCGAATAG

>Sperm_whale_Retnl_XM_007119293.1

ATGAAGCTGAACTTTTGCTTCCTTCTCATCCTCATCCTCCGTCACCAGATGACAATCCCAGGGAGTGCTCAGTGCTCCTTAGACTCCATTGTGGATAAAAAGATACAGGATGCTCTCACTGGTCTAGAGCTCAATCCCTTTGCCCCAGCAAAGAGGATCTCATGTTTCAGTGTCACGAACTCAGGCAAACTGTCTTTCTGTCCTGCAGGGTCGGTTGTCACTGGCTGTGCTTGTGGCTATGGCTGTGGCTCCTGGGATGTTGGGGGGGAAACCACATGCCACTACCAGTGCAACCCAGTAGATTGGACCACTGCCTGCTGCTGCCGCCTGACCTGA

>Minke_Whale_Retn_XM_007169061.1

ATGAAGGCTCTCTCCCTCCTCCTCCTCCCAGTCCTGGGGCTGCTGGTGTGTGGCAATTTGCTGTGTCCCATGGATAAAGCCATCAGTGAGAAGATCCAGGAAGTCACCAGCTCCCTAATTCCTGAGGCAATAAGGAACATTGGCCTGGACTGCCGGAGTGTCACCTCCAGGGGGGACCTGGCCTCTTGCCCCTCAGGTTTCGCCGTCACCGGCTGCACCTGTGGCTCTGCCTGTGGCTCGTGGGACGTGCGCGCCGAGACCACGTGCCACTGCCAGTGCGCCGGCATGGACTGGACCGGAGCTCGCTGCTGTCGCCTGCGGGCCGAGTAG

>Minke_whale_Retnl1_XM_007187131.1

ATGAAGCTGAACTTTTGCTTCCTTCTCCTCCTCCTCCTCCTCCTCCAGATGACAATCCCAGGGAGTGCTCAGTGCTCCTTAGACGCCATTGTGGATAAAAAGATAAAGGATGCTCTCACTGGTCTAGAGCTCAATCCCTCTGCCCCAGCAAAGAGGATCTCATGTTTCAGTGTCGTGAACTCAGGCAAACTGTCTTCCTGCCCTGCAGGGTCGGTTGTCACTGGCTGTGCTTGTGGCTATGGCTGTGGCTCCTGGGATGTCGAGGGGGGGGAAACCACATGCCACTACCAGTGCAACCCAGTAGATTGGACCACTGCCTGCTGCTGCCGCCTGACCTGA

>Yangtze_river_dolphin_Retn_XM_007460605.1

ATGAAGGCTCTCTCCCTCCTCCTCCTCCCAGTCCTGGGGCTGCTGGTGTGTGGCAAGTTGACGTGTCCCATGGATAAAGCCATCAGTGAGAAGATCCAGGAACTCACCAGCTCCCTAACTCCTGAGGCAATAAGGAACATTGGCCTGGAGTGCCGGAGTGTCACCTCCAGGGGGCACCTGGTCACTTGCCCCTCAGGTTTCGCCGTCACCAGCTGCACCTGTGGCTCTGCCTGTGGCTCGTGGGACGTGCGCGCCGAGACCACGTGCCACTGTCAGTGCGCCGGCATGGACTGGACCGGAGCTCGCTGCTGTCGCCTGCGGGCCGAATAG

>Killer_whale_Retn_XM_004277299.1

ATGAAGGCTCTCTCCCTCCTCCTCCTCCCAGTCCTGGGGCTGCTGGTGTGTGGCAAGTTGACGTGTCCCATGGATAAAGCCATCAGTGAGAAGATCCAGGAACTCACCAGCTCCCTAAATCCTGCGGCAATAAGGAACATTGGCCTGGACTGCCGGAGTGTCACCTCCAGGGGGCACCTGGTCACTTGCCCCGCAGGTTTCGCCGTCACCAGCTGCACCTGTGGCTCTGCCTGTGGCTCGTGGGACGTGCGCGCCGAGACCACGTGCCACTGTCAGTGCGCCGGCATGGACTGGACCGGAGCTCGCTGCTGTCGCCTGCGGGCCGAATAG

>Pig_Retn_ENSSSCT00000014827

ATGAAGGCTCTCTCCCTCCTCTTCCTCCCAACCCTGGGGCTGCTGGTGTGGGGCAAGTCGCTGTGTCCCGTGGATGAAGCCATCAATGAGAAGATCCGGGATGTCGCCAGTTTCCTAATTCCTCAGGTAATTAGGAACATTGGCCTGGAATGCCGGAGTGTCACCTCCAGGGGGGACCTGGTCACCTGCCCCTCAGGCTTTGCTGTCACTGGGTGCACGTGTGGCTCCGCCTGTGGCTCTTGGGACGTGCGCGCTGAGACCACCTGCCACTGCCAGTGCGCTGGCATAGACTGGACCGGCGCTCGCTGCTGTCGCCTGAGGACGCCATAG

>Pig_Retnl1_ENSSSCG00000011939

ATGAAGCCAACTTCTTGCTTCCTTCTTATCCTCATCCTCCTTCTTCAGTTGATAATCGCAAGGGGTTCTCAGTGTTCCTTAGACTCCCTTGTGGACAAAAAGATAAAGGATGCTCTCAATCGTCTAGAGCTCAATCCCTCTGCTCCAAGAAAGAGGATCTCATGTTTCAGTGTCAAAAACTCAGGCAAACTGTCTTCCTGCCCTGCAGGGACCGTTGTCACTGGCTGTGCTTGTGGCTATGCCTGTGGTTCCTGGGATGTCCAGCATGAAACCACATGCCACTGCCAGTGCAACACAGCAGACTGGACCGCTGCCCGCTGCTGCCGCCTGACATGA

>Alpaca_Retn1_XM_006206517.1

ATGAAGGCTCTCCCCCTCCTCCTCCTCCCAGTCCTGTGGCTGCTGGTGTGTGGCAACTCACTGTGTCCCATGGATGAAGCCCTCAATGAGAAGATCCAAGATGCCACCAGTTTCCTAATACTTGACGTAATAAGGAAAGTTCGCCTGGACTGCCGGAGTGTCACCTCCAGGGGGGACCTGGTCACCTGCCCCTCAGGCTTCGCTGTCACCGGCTGCACGTGTGGCTCTGCCTGTGGCTCGTGGGATGTTCGCGCCGAGACCACATGCCACTGCCAGTGCGCAGGCATGGACTGGACAGGAGCCCGCTGCTGTCACCTGCAGGCCTAG

>Alpaca_Retnl_XM_006215917.1

ATGAAGAGGACCTCCTGCTTCCTTCTCATCCTCATCCCCCTTCTCCAGCTGCTGATCCTAGGAGTTGCTCATGATTCCTTAGACTCCATTGTAGATAAAAAGATAAAGGATGCTCTCATTGGTCTAGAGCTCAATCCCTCTGCCCACCAAAAGACAATCTCATGTATCAGTGTCAAGAACTCAGGCAAACTGTCTTCCTGCCCTGCAGGGACTGTTGTCACTGGTTGTGCCTGTGGCTATGGCTGTGGTTCCTGGGATGTCCAGCAGGAAACCACGTGCCACTGCCAGTGTAACCCAGGAGACTGGACCACCGCTCGCTGCTGCCGCCTGATCTGA

>Camel_Retn1_XM_006177569.1

ATGAAGGCTCTCCCCCTCCTCCTCGTCCCACTCCTGTGGCTGCTGGTGTGTGGCACGTCACTGTGTCCCATGGATGAAGCCCTCAATGAGAAGATCCAAGATGCCACCAGTTTCCTAATACTTGACGTAGTAAGGAGAGTTCGCCTGGACTGCCGGAGTGTCACCTCCAGGGGGGACCTGGTTACCTGCCCCTCAGGCTTCGCTGTCACCGGCTGCACGTGTGGCTCTGCCTGTGGCTCGTGGGATGTTCGCGCCGAGACCACATGCCACTGCCAGTGCGCAGGCATGGACTGGACAGGAGCCCGCTGCTGTCGCCTGCAGGCCTAG

>Camel_Retnl_XM_006192297.1

ATGAAGAGGACCTCCTGCTTCCTTCTCATCCTCATCCCCCTTCTCCAGCTGATAATCCTAGGAGTTGCTCATGATTCCTTAGACTCCATTGTAGATAAAAAGATAAAGGATGCTCTTATTGGTCTAGAGCTCAATCCCTCTGCCCACCAAAAGACGATCTCATGTATCAGTGTCAAGAACTCAGGCAAATTGTCTTCCTGCCCTGCAGGGACTGTTGTCACTGGCTGTGCCTGTGGCTATGGCTGTGGTTCCTGGGATGTCCAGAAGGAAACCACGTGCCACTGCCAGTGTAACCCAGGAGACTGGACCACCGCTCGCTGCTGCCGCCTGACCTGA

>Ferret_Retn_ENSMPUG00000008210

ATGAAGGCTCTTCCTCTCCTCCTGCTCCCTGTCCTGGGGCTGCTGGTGTGTGGCAATTCTCTGTGTCCCGTGGACAAAGCCATCAGTGAGAAGATCCAGGATGACGCCCCATCCTTAATTCTTGAGACAATGAGGAACTTTGGCCTGACCTGCCAGAGTGTCACCTCCAGGGGGGACCTGGCCACCTGTCCCGCAGGCTTCGCAGTCACCGCCTGCACTTGCGGCTCCGCCTGCGGCTCGTGGGACGTGCGCGCCGAGACCACGTGCCACTGCCAGTGCGCGGGCATGGACTGGACCGGAGCTCGCTGCTGCCGCTTGCAGACCGCCGCCTGA

>Ferret_Retnl_ENSMPUG00000010050

ATGAAGTCTACCTCTTGCATTTTTCTCCTTCTCATCCTCGTCCAGCTGATGATCCCAGAGACTGCTCCGTGTTCCTTAGACTCTATCATGGATACAAAGATAAAAGAAGTTCTCAACGGCTTGGAGATAAGTACTTCTCCAGCAAAAAAGATGACATGTGTCAGTATCCAAAGCTCAGGCAGACTGTCCTCGTGCCCTGCAGGGATGGTTGTCACTGGCTGTGCCTGTGGCTATGGCTGTGGTTCCTGGGATATCCGGGGAGAAAACACATGCCACTGCCAGTGCAGCACAATTGACTGGACCACTGCTCGTTGCTGCCACCTGACCTGA

>Dog_Retn_XM_849220.3

ATGAAGGCTCTTCCCCTCCTCCTCCCTGTCCTGGGGCTGCTGGCGTGTGGCAAGTCTCTGTGTCCAGTGGATGAGGCCATCCACGAGAAGATCCAGGATGACACCAGCTCCTTAATTCTGGAGACAATAAGGAACTTTGGCCTGAGCTGCCAGAGCGTCACCTCCAGGGGGGACCTGGCCACCTGCCCGGCAGGCTTCGCCGTCACCGCCTGCGCTTGTGGCTCCGCCTGCGGCTCCTGGGACGTGCGTGTCGAGACCACATGCCACTGCCAGTGCGCGGGCATGGACTGGACCGCGGCGCGCTGCTGCCGCTTGCAGGCCACCGCCTGA

>Dog_Retnl_XM_003434071.2

ATGAAGACCACTTGTTGCTTCCTTCTCCTCATCTTCCTCCAGCTGATGATCCCAGGGACTGCTCCGTGTTCCTTGGACTTCATTCTGGATACAAAGATAAAGGAAACCCTCAGTGGCTTAGAGTTACATCCTTCCCCAACACGAAGGTTGTCATGTGTCAGTGTCAAGAATTCAGGCAGACTGTCCTCCTGCCCTGCAGGGATGATTGTCACTGGCTGTGCCTGTGGCTATGGCTGTGGTTCCTGGGATATCCAGGGCGAAACTACATGCCACTGCCAGTGCAGCACTATCGACTGGACCACCGCTCGCTGCTGCCACCTGACCTGA

>Amur_tiger_Retnl_XM_007098588.1

ATGAAGCTTACGTCTTACTTCCTTCTCATTCTCATCCTCCTTCTCCAGCTGATGAGCCCAAGGTATGCTCAGTGTTCCTTGGACTCCATCATGGATACAAAAATAAAGGAAGCTCTCACTGGCTTAGAAATAAATCCTGCCCCAGCAAAGAAGATTTCATGTACCAGTATCTTTAACTCAGGCAGACTGTCTTCCTGCCCTGCAGGAATGGTTGTCACCAGCTGTGCTTGTGGCTATGGCTGTGGTTCCTGGGATATCCAGGGAGAAACCACATGCCAATGCCAGTGCAGCACTATAGACTGGACCACTTCCCGCTGCTGCCACCTGACCTGA

>Weddell_seal_Retn_XM_006733818.1

ATGAAGGCTCTTGCCCTCCTCCTCCTCCCTGTCCTGGGGCAGCTGGTGTGTGGCAAGTCTCTGTGTCCAGTGGATGAAGCCATCAATGAGAAGATCCAGGATGGCACCAGATCCTTAATTCTTGAGACAAGGAGGAACTTTGGCCTGAGCTGCCGGAGCGTCACCTCCAGGGGGGACCTGGCCACCTGCCCCGCAGGCTTCGCCGTCACCGCCTGCACTTGTGGCTCCGCCTGTGGCTCGTGGGACGTGCGCGCCGAGGCCACATGCCACTGTCAGTGCGCGGCCATGGACTGGACCGGAGCTCGCTGCTGCCGCTTGCAGAGCACCGCCTGA

>Pacific_walrus_Retn_XM_004412162.1

ATGAAGGCTCTTGCCCTCCTCCTCCTCCCTGTCCTGGGGCTGCTGGGGTGTGGCAAGTCTCTGTGTCCAGTGGATGAAGCCATCAATGAGAAGATCCAGGATGGCACCCGATCCCTAATTCTTGAGACAATGAGGAACTTTGGCCTGAGCTGTCGGAGCGTCACCTCCAGGGGGGACCTGGCCACCTGCCCCGCAGGCTTCGCCGTCACCGCCTGCACCTGTGGCTCCGCCTGTGGCTCGTGGGACGTGCGCGCCGAGACCACATGCCACTGCCAGTGCGCGGGCATGGACTGGACCGGAGCTCGCTGTTGCCGCGTGCAGACCACCGCCTGA

>Pacific_walrus_Retnl_XM_004391620.1

ATGAAGTCTACCTCTTGCTTCCTTCTTATTCTCATCGTCCTCCAGCTGATGATCCCAGGGACTGCTCCGTGTTCCTTAGACTCCCTTGTGGATACAAAGATAAAGGAAGCTCTCAAAGGCTTAGAGTTAAATCCTTCCCCAACAAAAAGGATGTCATGTGTCAGTATCACGAACTCAGGCAGACTGTCCTCCTGCCCTGCAGGGATGGTTGTCACTGGCTGTGCCTGTGGCTATGGCTGTGGTTCCTGGGATATCCGGGGAGAAACCACATGCCACTGCCAGTGCAGCACGATGGACTGGACCGCTGCTCGCTGCTGCCACCTGACCTGA

>Horse_Retn_XM_001497441.2

ATGAAGGCCCTCTCCGTCCTCCTCCTCCCCGCCCTGGGACTGCTGGTATGCGGTGAGACGCTGTGTCCCGTGGATGAAGCCATCAATGCGAAGATCGGGACTGGTATCACCTCGCTAATTCTTGGGGTAATAAGGCAAGTGAACCTGGACTGCAGGAGCATCAGTAACCGCGGGGACCTGGCCACCTGCCCCAGAGGCTTCGCAGTCACTGGCTGCACGTGTGGCTCCGCCTGCGGTTCGTGGGACGTGCGCGCCGAGACCACCTGCCACTGCCAGTGCGCGGGCATAGACTGGACCGGAGCGCGCTGCTGCCGCATGCAGATCGCCGCCTGA

>Horse_Retbnl_XM_001503230.1

ATGAAGGTTACCTCTTGCTTCCTTCTCATCCTCATCCTCCTTCTCCAGCTGATGATCACAGGAAGTACTGAGTGTTGCTCAAACTCCGTTATGGATGGAAAGATAAAGAAAGCTCTCATCCGTCTAGGGTTCAATCCTTCTGCCCTGAAAAAGAAGATCTCATGTACCAGTGTCAAGAATTCAGGCAAACTGTCCTCCTGCCCTGCAGGGACTGTTGTCACCAGCTGTGCTTGTGGCTATGGCTGTGGTTCTTGGGATGTTCGGGGGGAAACCACGTGCCACTGCCAGTGCAACCCGGTAGACTGGACCACTGCCCGCTGCTGCCACCTGACCTGA

>Rhinoceros_Retn_XM_004443519.1

ATGAAGGCTCTCTCCCTCCTCCTCCTTCCCGCCCTGGGACTGCTGGTGTGTGGCAGGATGCTGTGTCCCGTGGATGAAGCCATGAATGAGAAGATCCAGGATGTTACCAGAGCCCTAATTCTTCAGGTAATAAGGAACATTGGTCTGGACTGCCGAACCATCAGCAACAGCGGGGACCTGGCCACTTGCCCCAGAGGCTTCGCTGTCACTGGCTGCGCGTGTGGCTCCGCCTGTGGCTCGTGGGACGTGCGCGCCGAGACTACATGCCACTGCCAGTGCAAGGGCATGGACTGGACGGCCGCGCGCTGCTGTCGCATGCAGCTCGCCGCCTGA

>Rhinoceros_Retnl_XM_004436597.1

ATGAAGCCTACCTCTAGCTTCCTTCTCATCCTCATCTCCCTTCTCCAGCTGACGATCCCAGGAAGTGCTCAGTGTTCCTTAGACTCCATTGTGAATGAAAAGATGAAGCAAGCTCTCATCCATCTAGAGTTCAATCCTTCTGCTCCTCAAAAGAAGATCTCATGTACCAGTATCAAGACCTCAGGCAAACTGTCCTCCTGCCCTGCTGGGACTGTTGTCACCGGCTGTGCTTGTGGCTATGCCTGTGGTTCCTGGGATGTCCGGGGGGAAACCACATGCCACTGCCAGTGCAGCTTGGTAGACTGGACCACTGCCCGCTGCTGCCGCCTGACCTGA

>Vampire_bat_Retn_ENSPVAG00000006942

ATGAAGGCTCTCTCCCTCCTCCTCCTCCCTGTCCTGGGGCTGCTGGTGTGTGGCAGGCTGCTGTGTCCTATGGATGAAGCTATCGATAAGAAAATCCAGGATGTCACCAGCTCCCTAACTCTTGAGGCGATGAAAAACATTGATCTGTACTGTCGGAGTGTCACCTCCAGGGGCGACCTGGTCACCTGCCCCACAGGCTTCGTTGTCACTGGCTGCACGTGTGGCTCTGCCTGTGGCTCGTGGGATGTGCGCGCAGAGAGAACCTGCCACTGCCAGTGCGCGGGCATAGACTGGACTGGAGCACGTTGCTGTCGTATGCTGGTCAGCGGCTGA

>Little_brown_bat_Retnl_ENSMLUG00000001459

ATGAAGCCTACCCCTTGCAGCCTTCTCATCCCCATCCTCCTTCTCCTGCTGATGAACGCAGGGAGGACTCAGCAGTCCTTAGATGCCATCGTGGATGAAAAGATAAAGGACGCTATCCAAAGTTTGGAGTGCAATCCACCTCCCCCAACAAGGATCTCATGTACCAGTGTCACCAACTCTGGCAAACTGTCCTCCTGCCCTCCAGGGACTTTTGTCACCGGCTGTGCTTGTGGTTATGGCTGTGGTTCCTGGGATATCCGAAGGGAAACCACCTGTCACTGCCAGTGCAGCGTGGTAGACTGGACCACTGCCCGCTGCTGCCAACTGATCTGA

>Black_flying_fox_Retn_XM_006917236.1

ATGAAGGCTCTCTCCCTCCTCCTTCTCCCTGTCCTGGGGATGCTGGTGTGTGGCAGGCTGCTGTGTCCTGTGGATGAAGCTATTGATAAGAAAATCCAGGATGTCACCAGCTCCCTAATTCTTGAGACGATGAAAAACATTGATCTGTACTGTCGGAGTGTCACCTCCAGGGGCGACCTGGTCACCTGCCCCACAGGCTTCGTTGTCACTGGCTGCACGTGTGGCTCTGCCTGTGGCTCGTGGGATGTGCGCGCAGAGAGAACCTGCCACTGCCAGTGCGCGGGCATAGACTGGACGGGAGCACGTTGCTGTCGTATGCTGGCCAGCGGCTGA

>Brandts_bat_Retnl_XM_005856994.1

ATGAAGCCTACCCCTTGCAGCCTTCTCATCCCCATCCTCCTTCTCCTGCTGATGAGCGCAGGGAGGACTCAGCAGTCCTTAGACGCCATTGTGGATGAAAAGATAAAGGACGCTCTCCAAAGTTTGGAGTGCAATCCACCTCCCCCAACAAGGATCTCATGTACCAGTGTCACCAACTCTGGCAAACTGTCCTCCTGCCCTTCAGGGACTTTTGTCACCGGCTGTGCTTGTGGTTATGGCTGTGGTTCCTGGGATATCCGAAGGGAAACCACCTGTCACTGCCAGTGCAGCGTGGTAGACTGGACCACTGCCCGCTGCTGCCAACTGATCTGA

>Davids_mytolis_Retnl_XM_006759569.1

ATGAAGCCTACCCCTTGCAGCCTTCTCATGCCCATCCTCCTTCTCCTGCTGATGAGCGCAGGGAGGACTCAGCAGTCCTTAGACGCCATTGTGGATGAAAAGATAAAGGATGCTCTCCAAGGTTTGGAGTGCAATCCACCTCCCCCAAGGATCTCATGTACCAGTGTCACCAACTCTGGCAAACTGTCCTCCTGCCCTTCAGGGACTTTTGTCACCGGCTGTGCTTGTGGTTATGGCTGTGGTTCCTGGGATATCCGAAGGGAAACCACCTGTCACTGCCAGTGCAGCGTGGTAGACTGGACCACTGCCCGCTGCTGCCAACTGGTCTGA

>Shrew_Retnl_ENSSARG00000012085

ATGAAGTCATTCTTTGGCTTCCTTTTCATCCTCATCTTCCTCCAGTCGATGATCCCAAAGAGTGCTCAATTTTCCTTTGACTCCATTGTGGATGAAAAGATAAAGGACGCTCTTGAAGATTTCAAATATCCCAGTCTGTCATGTACCAGTGTCAGCAGCCGAGGCAGACTGTCCTCTTGTCCTGAAGGAACCATTGTGACTAGTTGTGCTTGTGGCTTTGGCTGTGGTTCTTGGAATATTGAGGGAGAAGACACATGCCACTGCCAATGTCCCAGGATGGATTGGTCCACTGCCCGCTGCTGTCGTCTGAACTGA

>Hedgehog_Retn_XM_007532841.1

ATGAAGGCTTTCCTCCTCCTCCACCTCCTCCTCGTCCTCCTTTTGGTCCTGGTTGATGGCGAGTCGCTGTGTCCCATGGATGAAGCCATCAGTCAGAAGGTCCAAGAGGCCACCACGTCCCTGACTCAGCAGGCCATGAGCAAACTGGTCCTGAACTGCCGAACTGTGGTCTCCAGGGGTCACCTGGCCACCTGCCCGGCAGGCTTCGAGGTCACCGGCTGCACCTGCGGCTCGGCCTGCGGCTCCTGGGACGTGCGCGCGGACACCACCTGCCACTGCCAGTGCGCGGGCATGGACTGGACGGGGGCGCGCTGCTGCCGCGTGCACGCGGGCTAG

>Hedgehog_Retnl_XM_007522174.1

ATGAAGTCAACTGCTTGCTTCATGCTCACCTTCATTCTCCTTCTCCAGCTGATAATCTCAGGGAGTAATCAATGTTCCTTAGAATCAGTTGTGGATAAAAAGGTCATGGAAATTCTCAATAATCTAGAGATCAACTCATCACCAAAAAGAGAGATGTCATGTACCAGTGTCACCAACTCAGGAAAACTGTCCACTTGCCCAGCAGGAACTGTTGTCACTGGCTGTGCTTGTGGTTATGGCTGTGGTTCCTGGGATGTCCAGTCAGGAAACACATGCCACTGCCAGTGTAGTGGAATGGACTGGACCACTGCCCGATGCTGCCGCCTGACCTAA

>Cape_golden_mole_Retn_ XM_006868990.1

ATGAATGTTGTCTGCTTTTACCTCCTCCCCGTTCTGGGGCTACTGGTGTCTGGTGTGTCCTTGTGTCCCGTGGATGAAGCCATCAATCAGAAGATCCAGAAAGACTCCAGCTCCCTAATTCGTGAGACTATGAGAGGCCTTCACCTGGATTGCAGAAGTGTCACCTCCAGGGGAGACCTGGCCAGCTGCCCTGCAGGGTTCGCCGTCACTAGCTGCACGTGTGGCTCTGCCTGTGGTTCCTGGGACATTCGTGCTGAGACCACCTGCCACTGCCAATGCTCGGGTATGGACTGGACCGGAGCACGCTGCTGCCGCCGCTGCCTTGAGAGCTTACCTGCGCAATGC

>Star_nosed_mole_Retn_XM_004695817.1

ATGAAGGCGCTCTTCTTCCTCCTCCTGCTGCTGGGCCTCGGGCTCCCTGCCCACGCCGACCAGTGCGTGGTGGACGACTCCATCGCCCAGAAGGTCCAGGAAGGCATTAGCTCCCTAAAAACTAACCTAAGGTCCGCGAGCCTGGAGTGCCGGAGTGTTACCTCCAGGGGGGGTGTGGCAGCCTGCCCCGGAGGCTTCGTTGTCACGGGCTGCGCGTGTGGCTCTGGCTGCGGCTCGTGGGACGTGCCCGCCGAGACCACGTGCCACTGCCAGTGCCGGGGCATGGACTGGACCACGGCACGCTGCTGTCGCCTGCAGGTGTCCCTCTGA

>Cape_elephant_shrew_Retn_XM_006902673.1

ATGAAGGCTTTTCCCCTGTTCCTCCTCCCTGCCTTGGGACTGCTAGTGTTCGGAGAGTCCCAGTGTTCCATGGATGAAGCCATTGACAAGAGGATCCAGCAGGGAACCAGCTCTCTGATTCTTGAGGCTGTTGGGAGAATTCTCATGGACTGCCGAAGCGTTACCTCCAGGGGACACTTGGCCACCTGCCCCGCAGGTTTCGCCGTATCTGCCTGCTCGTGTGGCTCTGCCTGTGGCTCGTGGGACGTGCGTGCGGAGACCACCTGTCACTGCCAATGCTCGGGCATGGACTGGACAGCAGCGCGCTGCTGCCGCATCAAAGTCGCAGCCTAA

>Cape_elephant_shrew_Retnl1_XM_006895349.1

ATGAAGCTTGCCTTTTGTCTCTTCCTGCTCATCCTCCTTCTCCAGCTGATTATCCCAGGGGATATGCAGTGTTCCTTAGATGCAGTCGTGGATGAAAAGATAAATGCAGCTCTCAGCAAACAGACAAAGAAGCTCACATGTACCAGCATCATTCTCAGTGGCAAACTGGCCTCCTGCCCTACAGGGTCTGCTGTCACCAGTTGTACCTGTGGCTATGGCTGTGGCTCCTGGGTTGTGCAGAATGGAAACACGTGCCACTGTCAGTGCAATGTGGTGGGCTGGACCGCAGCCCGCTGCTGCCACCTGTCCTAA

>Elephant_Retn_XM_003421304.1

ATGAAGGCTGTCTTCCTCCTCCTCCTCCTCCTCCCTGTCCTGGGGCTGCTGGTGTCTGGCGAGTCCCGGTGTCCTGTGGATGCAGCCATCGATCAGGAGATCCAGAGAGGCACCATCTCCCTAATTCTTGAGGCTTTTAACGGCCTTCGCCTGGACTGCCAAAGCGTGACCTCCAGGGGGGATCTGGCCACCTGCCCAGCAGGTGAGTCCGTCACTGGCTGCGCGTGTGGTTACGGCTGTGGCTCATGGGACGTGCGTGCAGAGACCACGTGCCACTGCCAGTGCCAGGGCATGGACTGGACTACAGCACGCTGCTGCCGCATGCTGCTTTCAAAATGA

>Manatee_Retn_XM_004378593.1

ATGAAGGCTGTCTCCCTCTTCCTCCTCCTCCCTGTCCTGGAGCTGCTGGTGTCCGGCGAGTCCTTGTGTCCCGTGGATGCAGCCATCAATCAGAAGATCCAGAAAGGCACCATCTCCCTAATTCTTGAGGCTCTGAGGAGAATCCACCTGGACTGCCGAAGCGTTACCTCCAGTGGGGACTTGGTCACCTGCCCATCAGGTTTCGCTGTCACTAGCTGCACATGTGGCTCCGCCTGCGGCTCTTGGGACGTGCGTGCAGAGACCACGTGTCACTGCCAATGCGCTAACATAGACTGGACCGGAGCGCGCTGCTGCCGCATACAGGCCTCAATCTGA

>Hyrax_Retn_ENSPCAG00000007052

ATGAAGACTGTCTCCCTACTCCTTCTCCTCCTCCTCACCCTGGGGCTGCTGGTGTCTGGCCACTCTCTGTGTCCTGTGGAATCAGCTATCAATCAGAAGATCCACGAAGGCACCAGCTCCATAATTCTTGAGGTGCTCAGGAGTATTCACTTTAACTGCCAAACTGTCACCTCCAGGGGAGACCTGGCCACGTGCCCAGCAGGTTTTGCTGTCACTGGCTGTGCATGTGGTTCTGCCTGTGGCTCATGGGATGTGTGGGCAGAGACCGTGTGCCACTGCCAGTGTGCGGGCATGGACTGGACCGGAGCCCGCTGCTGCCACTTGCAAGGCTGA

>Armadillo_Retnl_ENSDNOG00000009661

ATGAAGCCTACCACTTGTCTCCTTCTCATCATCATCCCCCTTCTACAGCTGGCAATCCCAGGGAGTGCTCAGTGTACCTTAGACTCCAGTGTGGATACAAAGATAAAGGCCGTCCTCAGCGAACTGGAGTATAAACACTCTGCTCCGATGAAGAAGCTCTCATGCTTCAGTGTCAAAAACACAGGCAGACTGTCCTCCTGCCCTACAGGGACCACAGTCACTGGTTGTTCTTGTGGCTATGCCTGTGGTTCCTGGGATGTCCGTGGGGAAAACACCTGCCACTGCCAGTGTGCTGTGATAGACTGGACCGCTGCCCGCTGCTGTCACCTGTCCTGA

>Tenrec_Retn_XM_004717352.1

ATGAAGGTGGTCTCATTGCTCCTCTTCCCCACTCTGGGGCTGCTGGTGTCGGGGGAGTCCCTGTGTCCTGTGGATGAAGCCATCAACCAGAAACTCCAGCAAGGCACCAGCACCCTACTTCTCGAGGCTGTCAGGAACATGAAGCTGGAGTGCCGAAGTGTCACCTCCAGGGGGGACCTGGCCACCTGCCCTGCAGGTTTCGCTGTTACTGGCTGCACGTGTGGCTCCGCCTGCGGCTCCTGGGACGTGCGCTCGGAGACCACGTGCCACTGCCAATGCAGAGGCATGGATTGGACCGGAGCGCGCTGCTGCCGCATGCAGGCCTGA

>Tenrec_Retnl_XM_004706464.1

ATGAAGCCCGCGTCTCACTTCCTTCTCATGCTCATCCCCCTCCTCCAGCTGATGATCCAAGGGGATGCTCAGTGTTCCTTAGACTCGGCTGTGGAACAAAAGATACAGTCAGCCATCAAGGAACTAGAAAATAAACCCCGGCCTCCGCCCCCAACACGGAAGCTTGTGTGTGTCAGTGTCAAGAGTCGAGGCAAACTGGCTGTCTGCCCTCCAGGGACCGTTGTCACCGGCTGTGCCTGTGGGTACGGCTGTGGCTCCTGGGATGTGCAAAGGGAAACCACGTGCCATTGCCAGTGCAGCGGGATGGATTGGACCGCAGCCCGCTGTTGTCACATGGCCTGA

>Aardvark_Retn_XM_007953702.1

ATGAAGGCTGTCTCCCTCCTCCTCCTCCCTGTCCTGGGGCTGCTGGTGTCCGGCAAGTCCCTGTGTCCTGTGGATGAAGCCATCAATCAGAAGATCCAGGAGGGCACCAGCTTCCAATTCCTGCGGGCTGTCAGGAACATCCACCTGGACTGCAACACTGTCACCTCCAGGGGGGACCTGGCCTCCTGCCCTGCAGGTTTTGCTGTCACCGGCTGCACATGTGGCTCTGCCTGTGGTTCATGGGACGTGCGTGCCGACACTACCTGCCACTGCCAATGCGCTGGCATTGACTGGACCGGAGCTCGCTGCTGCCGCGTGCAGATCTTATTTTGA

>Aardvark_Retnl_XM_007946564.1

ATGAAGCCTGCCTCTTGCTTCCTTCTCATGTTCATCCCCTTCGTGCGGCTAATGACCCCAGGGGACGCTCAATGTTCCTTACATAGGATTGTGGATGAAAAGATAAAGGCAGCACTCAACAAACTGGAATATAAGCCCTTTGCCCCAACACCAAAGCTCTCGTGTGTCAGTGTCACCACCCCAGGCAAACTGGCCTCCTGCCCTGAAGGGACTGCTGTCACTGGTTGTGCCTGTGGCTTTGCCTGTGGTTCCTGGGATGTGCAAGGGGAAAGTACATGCCACTGCCAGTGCAGTGTGGTAGACTGGACCACAGCCCGCTGCTGCCACGTGTCTTAA

>Opossum_Retn_XM_007489545.1

ATGAAGAGTGCCGCCATCTGCTTCTTGCTCACCATTCTTGGGCTCCTGGTCCCAGGGCAGGCTCAGTGCCCCCTGGACGAGCTTCTAAACAAGAAGATTGAGGAGCAGGCTGGCTCCTATGTTCTGACTGCGCTTGAGAAGCTTCAACTAGCCTGCTCCAGCGTTACCTCTCGGGGTGCTCTAGCCACCTGCCCCTCAGGGTTCACAGTCTCAAGCTGTTCCTGCGGCTCAGCATGCGGCTCCTGGGATGTTCGTGAGGCCACCACTTGTCACTGCCAATGTGCTGGAATGGACTGGACTAGTGCCCGCTGCTGCAAGTTGGGGAGCAAGGGCTGA

>Opossum_Retnl1_ENSMODG00000018074

ATGAAACCTGCCTTCTTTCTCCTGCTCATCCTCATCCCCCTTCTAGCACTGATGTCCCCTGGCCATGCTGACTGCGCCTTAGACTCCATTGTGGACAAGAAAATAAAGGAATCTCTTACCTCATTAGAGCTTGGATTTCAGGCTCGCCTCACTTGTACAAGTGTGAAGTCCAGAGGCACACTGGCCACTTGCCCAGCAGGGTTTATAGTCACTGGCTGTGCCTGTGGTTATGGCTGTGGCTCCTGGGATGTTCGAGGGGACAACGTATGCCATTGTCAGTGCTCAGGCATGGACTGGACCAGTGCTCGTTGCTGCCAATTTTCCAAATGA

>Wallaby_Retn_ENSMEUT00000015851

ATGCAGACTGCCGCCACCTGCCTCTTCCTCATCTTCCTCATCCTTCCTGGCCACCTGGTCCTAGGGCAGGATCACTGTTCTCTAGATGAGCTCTTAAACAAGAAGATGGAGGAGCAGGCCAGCTCCTATGTTCTGGCTGCCCTTCAAAAACTTCGAATGACCTGTTCCAGTGTTACCTCTCGGGGTGCTCTAGCCTCCTGCCCCTCAGGGTCCATTGTCACAGGCTGTTCATGTGGCTCAGCATGTGGCTCCTGGGATGTCCGTGAGCCCACCACTTGTCACTGTCAATGTGCTGGAATGGACTGGACTACTGCCCGCTGCTGCCAGTTTAGGGGCCAATGA

>Wallaby_Retnl1_ENSMEUG00000010781

ATGAAACCTGCCTTCGTTTTCCTGCTCATCCTCGTCCCCCTTCTAGCACTGATGTCCCCTGGCCAAGCTGACCCCTCCTCAATTGCGGAGAACACAGCACAGAAAGCTCTTTCCAAATTAGAGACTAGACATCAAGGACACCTCTATTGTATAAGCGTGAAAAGCAACGGCAGACTCTCCTCTTGCCCCAGAGGATTTGCAGTTACCAGCTGTGCCTGTGGCTATGGCTGTGGTTCCTGGGATGTTCAAGGGGACCAAACATGTCATTGTCAGTGCCAGGTCATGGACTGGACCACTGCACGATGCTGCAGGAATTGA

>Tasmanian_devil_Retn_XM_003763278.1

ATGACTGCCTCCTTCTGCCTCTTCCTCATCTTCCTCATCCTTCCTGGGCAGCTGGTCTTAGGACAGTCTCAATGTTCCCTAGATGATCTCTTAAACAAGAAGATCGAGGAGCAGGCCAGCTCCTATGTGCTGGCCACCCTTCAAAAGCTTCAACTGATCTGCTCCAGTGTGACTTCTCGGGGTGCTCTAGCCACCTGCCCCTCAGAATCCACTGTCACAGGCTGTTCATGTGGCTCGGCGTGTGGCTCCTGGGATGTTCGTGGGAGCACCACTTGTCACTGTCAATGTGCTGGAATGGACTGGACTAGTGCCCGCTGCTGCAGGTTTGGGAACTGA

>Tasmanian_devil_Retnl1_ENSSHAG00000015202

ATTAAATCTTTCTTCTTTTTCCTGCTCATTCTCATCCATCTTCTAGCACTACTGTCCCCTGGCCATGCTGGTTGCGTCTCAAAGGAAGACTTAGATGCAATTGTGCAGAAGAAAGTAAATGAAGCTTTTTCCAAATTAGAGACTGGAAATAAGAGAACTCTCTCTTGTATAAGTGTGAAAAGTGGAGGCACCCTAGCCACTTGCCCTGCAGGGTTTGTAGTTACTGGCTGTGCCTGTGGCTATGGCTGTGGCTCCTGGGATGTTCGAAGAGACAACACATGTCATTGTCAGTGTAGGGGCATGGACTGGACCAGTGCTCGATGCTGCAAGAATTCCTAG

>Tasmanian_devil_Retnl2_ENSSHAG00000015983

ATGAAACCTGCTCTCCTTTTCCTGCTCATTCTCATGCACCTTCTAGCACAACTATCTCCTGGCCATGCTGAATGCTGCTTAAAAGAAATAGAATTCATTGTGGAGAAGAAAGTACAAGATGCTCTTTCCACATTAGAGATCGGAACTAAGAGACCCCTTTCCTGTACAAGTGTGACAAATCGAGGCACCCTAGCCACTTGTCCTGCAGGGTTTATAGTTACTGGCTGTGCCTGTGGCTATGGCTGTGGCTCCTGGGATGTTCGAGGAGACAACACATGTCACTGTCAGTGTCAGGGCATGGATTGGACCAGTGCTCGATGCTGCACGAATTCCTAA

>Platypus_Retn_ENSOANT00000012518

ATGAAGGCTGCTCTCCTCCTTTCCTTCCTTCTCCCCTTCTTTGGGCTCTTGGCCTTGGGGAGCACTCAGTGGATCCTGGACACGGACGTGATCGAGAAGATCCAAGAGAGAGTCAACTCCCTCGTGTCGTCTGCTCTTAAGGACCTCAGCCTTGTCTGCACCAGTGTCTTTACCCAAGGAGCCCTGGTGGCCTGCCCTTCAGGTTACAAAGCTACGGCCTGTTCCTGTGGCTCGGCTTGTGGCTCGTGGGACATCCGCTCAGACACCGTGTGCCACTGTCAGTGCAGGGGGATCGACTGGACCTCTGCCCGCTGCTGCCAGATAAGGACCGCCTGA

>Anole_lizard_Retn1_ENSACAG00000029691

ATGAAAGCCATTTTGGTCCTGCTCTTTGTGGTGCTGATTCCTGCAAAGTATGCAAGTGCTCAGAGATGCCCTGTTGAGGATGCTATCGATGCAAAGATCAGTGCTGCCATTGATTCCAAAGTGTCTATACTTGTGCCAGAGCTGAGTTGCACCAGTGTGTCAGCCCGAGGTGCTGATGTCTCCTGCCCTTCTGATTACAAGGCCACTGGATGTGCTTGTGGGATGGCCTGTGGTTCTTGGGACATCCGTGGTGAAACCCAGTGTCACTGCCAATGTGCCAACATTGACTGGACTTCTGCTCGGTGCTGTAAAGTTGTATTGTCTGGTGGCTTCTCTGGTTAG

>Anole_lizard_Retn2_ENSACAG00000028837

ATGATAAAAGTCATTTTGTTCCTGCTCCTTGTTGTGTTGATTCCTGCAAATTATGCAAATGCTCAGGCATGTTGCAGTATTGATCGCGCTATTGACGCGAAGATCAAGGCCGCTGTTGATTCCAAAGTGTCTGCAACTTTAGCAAAGAGCCAGCTGACATGTACCACCATTAAAACCAGTGGAGCTCTTGCTGCCTGCCTTCATGGTTACACAGTCACTGGATGTTCCTGTGGAAAGGCCTGTGGTTCTTGGGACGTCCGTGATAACTCCACGTGTCACTGCCAATGTGCCAACGTTGACTGGACTGCTGCTCGGTGCTGTAAAATAGTTCGCTGA

>American_alligator_Retn1_XM_006265086.1

ATGGCACTGCTGCTGATGCTGCTGCCCCTGCTGCTGCCCATGGTGGTGAGCCAGTCCGAGTGCCCCCTTGACAGTGTGGTCAACCTCAAGATCCAGGCGGCACTAGCTGGATACTGCCCCACCCCACAGGAGCCAGCTGTCAACTCCGCAGAGCTGGTGTGTACTGATGTGAATGCCCGTGGGAGCCTGGTCTCCTGCCCCAGTGGGTTCAAGGCGACAGCCTGCGCCTGTGGCATGGGCTGCGGCTCCTGGGACATCCGCGGTGACACCTCCTGCCACTGCCAGTGCCAAGGAATAGACTGGACCAGTGCCCGCTGCTGCCGTATCCAGCTCCACCGTGCCTGA

>American_alligator_Retn4_XM_006265093.1

ATGCCCCCCGGATTGGGTGGCCAGGCCGACTGCCCCATTGACAGCGTGGTCAACCTCAAGATGCAGGCGGCGCTTGCTGGGAAGTGCTGCGGCTCGGGGGAGCCAGCCGTTTACTCTGCTGAGCTGGTGTGTACAAACGTGGTTGACAATGGAAGCCTGGCCTCCTGCCCCAACGGGTACATAGCGACGGGCTGCTCCTGTGGCATGGCCTGTGGCTCCTGGGACATCCATGCCAACTCTACCTGCCAGTGCCAGTGCCAGGGCATAGACTGGACCAGCGCCCGCTGCTGCCGCATCCAGCTGCAACGCACCTGA

>Chinese_alligator_Retn1_XM_006036482.1

ATGGCGCTGCTGCTGATGCTGCTGCCCCTGCTGCTGCCCACGGTGGTGGCCCAGTCCGAGTGCCCCCTTGACAGTGTGGTCAACCTCAAGATCCAGGCGGCACTAGCTGGATGCTGCTCCACCCCACAGGAGCCAGCTGTCAACTCCGCGGAGCTGGTGTGTACTGATGTGAATGCCCGTGGGAGCCTGGTCTCCTGCCCCAGTGCTGTCAACTCCGCGGAGCTGGTGTGTACTGATGTGAATGCCCGTGGGAGCCTGGTCTCCTGCCCCAGTGGGTTCAAGGCGACAGCCTGCGCCTGTGGCATGGGCTGCGGCTCCTGGGACATCCGCAGTGACACCTCTTGCCACTGCCAGTGCCAAGGAATAGACTGGACCAGTGCCCGGTGCTGCCGTATCCAGCTCCACCGTGCCTGA

>Green_sea_turtle_Retn1_XM_007072480.1

ATGAAGGCTGCCGTGTTCCTGCTGCTCGCCCTCCTGGTGCCCGCGGGCCACACAGCGGCTCAGTGCCTCATTGACAATGTGGTTGATCTGAAGATGCAGGCAGCGATTAGTTCCATAGTGTCCGCCACCCTGGCCAAAGCCAAGCTACTCTGCCAGGACGTCTTGGCACGTGGGGCACTTGTCTCCTGCCCAGCAGGGTACAAGCCCACGGGCTGCGCCTGCGGAATGGGCTGCGGCTCCTGGGACATTCGCACCGACTCCACCTGCCACTGCCAGTGCGCCGGCATCGACTGGACGGCCGCACGCTGCTGCAAGATAGGCCTGGAGTGA

>Painted_turtle_Retn1

ATGAAGGCTGCCGTGTTCCTGCTGCTCACCCTCCTGGTGCCCGCGTACCACACGGATGCTCAGTGCATCATTGACAATGTGGTCGATCTGAAGGTGCAGGCAGTGGTTAATTCCATAGTGTCCTCCACCCTCGCCAAAGCCAAGCTACTCTGCCAGGATGTGTCAGCCCGTGGGGCACTTGTCTCCTGCCCAGCAGGGTACAAACCCACGGGCTGTGCCTGTGGAATGGCCTGCGGCTCCTGGGACATTCGCACCGACTCCACCTGCCACTGCCAGTGCGGCGGCATCGACTGGACGGCCGCGCGCTGCTGCAAGATAGGACTGGAGTGA

>Coelacanth_Retn_XM_006002924.1

ATGAAGACTGCACTTCTGATGTTAATACTCATTCTTGGCTCCAGTTGCCTGGCTGATCCCCAGTGCAACATTCAGGACTGGCTCTCTCTAGGGAGTGAGGCTATTTTAAAGGCCTTAGCTGAAAATGTGATGCAGAAAGCAAAGTTGGTTTGTACAGATGTATCCTCTCGTGGAGATGTTGCTACATGCCCAACAGGTTCCAAACCCACTTCCTGCTCCTGCGGAATGGCCTGTGGCTCCTGGGACATTCGAAATGATCAAACCTGTCATTGTCAGTGCAACAATATTGACTGGACCAGCGCCCGCTGCTGCAAGATAGCCTTCTAG

**S1 Fig. Coding sequences of intact *Retn* and *Retnl* genes**.

Full-length coding sequences for *Retn* and *Retnl* identified from diverse vertebrate and used in this analysis are presented in fasta format. NCBI accessions or Ensembl gene IDs are provided, if available, after the common name for each species. Sequences are from Table S1.
