# Supplementary material for: Evolution of the Vertebrate Resistin Gene Family
Source: PLoS One. 2015 Jun 15;10(6):e0130188. doi: 10.1371/journal.pone.0130188 (PMC4467842; doi:10.1371/journal.pone.0130188)
Supplement: S2 Fig — (PDF) [file pone.0130188.s002.pdf]

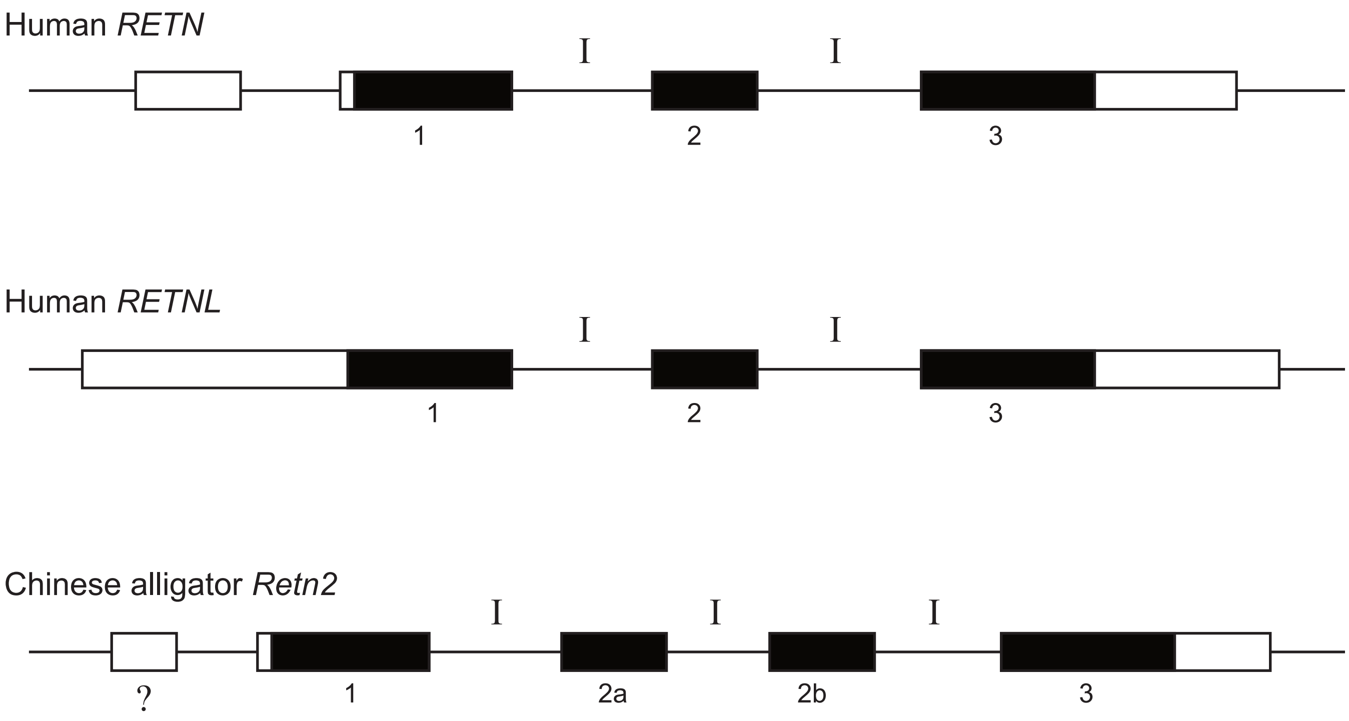

S2 Fig. Duplication of exon 2 in *Retn2* from the Chinese alligator.
Exon-intron structure of the human *RETN* and *RETNL* genes and the Chinese alligator *Retn2* gene. Exons are shown as boxes, with solid boxes being coding sequence and open boxes being untranslated regions. The thin line is intron and flanking sequence. Exons are to scale, while introns are not. Coding exons are numbered, with their number shown below the exons. The alligator gene has a duplicated exon 2, labeled as 2a and 2b. Additional untranslated exons may exist in the alligator gene (indicated by the question mark). Phase of the introns is shown by roman numerals, with I indicating that the introns inturrupt the coding region after the first base of a codon.
