# Supplementary material for: Evolution of the Vertebrate Resistin Gene Family
Source: PLoS One. 2015 Jun 15;10(6):e0130188. doi: 10.1371/journal.pone.0130188 (PMC4467842; doi:10.1371/journal.pone.0130188)
Supplement: S3 Fig — (PDF) [file pone.0130188.s003.pdf]

```
                 M   K   A   L   P   L   L   L   V   P   L   L   W   L   L   V   C   G   T   S
Camel Retn      ATG AAG GCT CTC CCC CTC CTC CTC GTC CCA CTC CTG TGG CTG CTG GTG TGT GGC ACG TCA
Alpaca Retn     ATG AAG GCT CTC CCC CTC CTC CTC GTC CCA GTC CTG TGG CTG CTG GTG TGT GGC AAC TCA
Camel ψRetn     --- --- --- --- --- --- --- --- --- --- --- --- --- --- --- --- --- --- --- ---
Alpaca ψRetn    --- --- --- --- --- --- --- --- --- --- --- --- --- --- --- --- --- --- --- ---

                 L   C   P   M   D   E   A   L   N   E   K   I   Q   D   A   T   S   F   L   I
Camel Retn      CTG TGT CCC ATG GAT GAA GCC CTC AAT GAG AAG ATC CAA GAT GCC ACC AGT TTC CTA ATA
Alpaca Retn     CTG TGT CCC ATG GAT GAA GCC CTC AAT GAG AAG ATC CAA GAT GCC ACC AGT TTC CTA ATA
Camel ψRetn     --- --- --- --- --- --- --- --- --- --- --- --- --- --- --- --- --- --C CTA ATT
Alpaca ψRetn    --- --- --- --- --- --- --- --- --- --- --- --- --- --- --- --- --- --C CTA ATT
                                           Exon 1                                       >>>><<

                 L   D   V   V   R   R   V   R   L   D   C   R   S   V   T   S   R   G   D   L
Camel Retn      CTT GAC GTA GTA AGG AGA GTT CGC CTG GAC TGC CGG AGT GTC ACC TCC AGG GGG GAC CTG
Alpaca Retn     CTT GAC GTA ATA AGG AAA GTT CGC CTG GAC TGC CGG AGT GTC ACC TCC AGG GGG GAC CTG
Camel ψRetn     CTT GAG GTA ATA AGG AAT CTT GGC TTG AAC TGC CGG AGT GTC CCC TCC AGG GGG AAC TTG
Alpaca ψRetn    CTT GAG GTA ATA AGG AAT CTT GGC TTG GAT TGC CGG AGT GTC CCC TCC AGG GGG AAC TTG
                <<                                    Exon 2

                 V   T   C   P   S   G   F   A   V   T   G   C   T   C   G   S   A   C   G   S
Camel Retn      GTT ACC TGC CCC TCA GGC TTC GCT GTC ACC GGC TGC ACG TGT GGC TCT GCC TGT GGC TCG
Alpaca Retn     GTC ACC TGC CCC TCA GGC TTC GCT GTC ACC GGC TGC ACG TGT GGC TCT GCC TGT GGC TCG
Camel ψRetn     GCC ACC TGC TCC TCA GGC TTC AGC GTC ACT GGC TAC ACG TGT GGC TCC ACC TGT GGC TCG
Alpaca ψRetn    GCC ACC TGC TCC TCA GGT TTC AGT GTC ACT GGC TAC ACG TGT GGC TCC ACC TGT GGC TCG
                                 >>>><<<<                       Exon 3

                 W   D   V   R   A   D   T   T   C   H   C   Q   C   A   G   M   D   W   T   G
Camel Retn      TGG GAT GTT CGC GCC GAG ACC ACA TGC CAC TGC CAG TGC GCA GGC ATG GAC TGG ACA GGA
Alpaca Retn     TGG GAT GTT CGC GCC GAG ACC ACA TGC CAC TGC CAG TGC GCA GGC ATG GAC TGG ACA GGA
Camel ψRetn     TGG GTC ATA CTT CCC AAG ACC ACA TGG AAC TGC CAG C-- GCG GGT GTG GAC TGG ACA GGA
Alpaca ψRetn    TGG GTC ATA CTT CCC AAG ACC ACG TGG AAC TGC CAG C-- GCG GGT GTG GAC TGG ACA GGA

                 A   R   C   C   R   L   Q   A   *
Camel Retn      GCC CGC TGC TGT CGC CTG CAG GCC TAG ---
Alpaca Retn     GCC CGC TGC TGT CAC CTG CAG GCC TAG ---
Camel ψRetn     GCC CAC TGC CGT CAC AAG CAG GCC ATG TAA
Alpaca ψRetn    GCC CAC TGC CGT CAC AAA CAG GCC ATG TAA
```

S3 Fig. Processed *Retn* pseudogenes in the camel and alpaca.
Alignment of partially processed *Retn* pseudogenes and the coding sequences of intact *Retn* genes from the camel and alpaca. Dashes are gaps introduced to maximize the alignment. The protein sequence (in single letter code) predicted from camel *Retn* is shown above the DNA sequences, which is separated into codons. Base changes and gaps marked in red disrupt the coding sequence in the pseudogenes. Boundaries of the exons within the coding sequence of *Retn* genes are indicated by >>>> or <<<< below the sequences. Intronic sequence exists in the intact camel and alpaca *Retn* genes, but not in the processed pseudogenes.
