# Supplementary material for: Evolution of the Vertebrate Resistin Gene Family
Source: PLoS One. 2015 Jun 15;10(6):e0130188. doi: 10.1371/journal.pone.0130188 (PMC4467842; doi:10.1371/journal.pone.0130188)
Supplement: S5 Fig — (PDF) [file pone.0130188.s005.pdf]

```
                 M   G   P   S   S   C   L   L   L   I   L   I   P   L   L   Q   L   I   N   P
Human RETNL     ATG GGG CCG TCC TCT TGC CTC CTT CTC ATC CTA ATC CCC CTT CTC CAG CTG ATC AAC CCG
Human ψRETNL    TTC AAG TTC ATC TCT TCT GTC CT- CTG ACT TCT CAC CCC CTT CTT AAA TAG CAC TAC TAA
Chimp ψRetnl    CTC AAG TTC ATC TCT TCT GTC CT- CTG ACT TCT CAC CCC CTT CTT AAA TGG CAC TAC TAA
Orang ψRetnl    TTC AGG TTC ATC TCT TCT GTC CT- CTG ACT TCT CAC CCC CTT CTT AAA TGG CAC TAC TAA
Macaque ψRetnl  TTC AAG TTC ATC TCT TCT GTC CT- CTG ACA TCT CAC CCC CTT CTT AAA TGG CAC TAC TAA
O_baboon ψRetnl TTC AAG TTC ATC TCT TCT GTC CT- CTG ACA TCT CAC CCC CTT CTT AAA TGG CAC TAC TAA

                 G   S   T   Q   C   S   L   D   S   V   M   D   K   K   I   K   D   V   L   N
Human RETNL     GGG AGT ACT CAG TGT TCC TTA GAC TCC GTT ATG GAT AAG AAG ATC AAG GAT GTT CTC AAC
Human ψRETNL    --- --- --- --- --- --- --- --- --- --- --- --- --- --- --- --- --- --- --- ---
Chimp ψRetnl    GGT GGA GAA AGG GTA GTG ACA GGG AGA GGA GTT GTG AGC AGG GAT GAG GTG GGG CAG GCA
Orang ψRetnl    GGT GGA GAA AGC GTA GTG ATG GGG AGA GGA GTT GTG AGC AGG GAT CAG GTG GGG CAG GTA
Macaque ψRetnl  GGT GGA GAA AGG GTA GTG ATA GGG AGA GGA GCT GTG AGC AGG GAT GAG GTG GGG CAG GCA
O_baboon ψRetnl GGG AGA GAA AGG GTA GTG ATA GGG AAA GGA GCT GTG AGC AGA GAT GAG GTG GGG CAG GCA
                                                                     EXON 1

                 S   L   E   Y   S   P   S   P   I   S   K   K   L   S   C   A   Y   V   K   S
Human RETNL     AGT CTA GAG TAC AGT CCC TCT CCT ATA AGC AAG AAG CTC TCG TGT GCT AGT GTC AAA AGC
Human ψRETNL    --- --- --- --- --- --- --- --- --- --- --- --- --- --- --- --- --- TTC CAA ATC
Chimp ψRetnl    GAA GTA AGG ACT CCT GT- --- --- --- --- --- --- --- --- --- --- --- CTC CAA ATC
Orang ψRetnl    GAA GTA AGG ACT CCT GT- --- --- --- --- --- --- --- --- --- --- --- CTC CAA ATC
Macaque ψRetnl  GAA GTA AGG ACT CCT CT- --- --- --- --- --- --- --- --- --- --- --- CTC CAA ATC
O_baboon ψRetnl GAA GTA AGG ACT CCT CT- --- --- --- --- --- --- --- --- --- --- --- CTC CAA ATC
                     >>>><<<<      EXON 2

                 Q     G       R     P   S   S     C     P     A   G   M   A   V   T   G   C   A
Human RETNL     CAA GG----C A--GA CCG TCC TCC- TGC CC--T GCT GGG ATG GCT GTC ACT GGC TGT GCT
Human ψRETNL    CAA GGGCTCC AACGT CTC TGC TCCA TGC CCCAT GCA GGG TAC CCT GTC ACT GGT CAT GCT
Chimp ψRetnl    CAA GGGCTCC AACGT CTC TGC TCCA TGC CCCAT GCA GGG TAC CCT GTC ACT GGT CAT GCT
Orang ψRetnl    CAA GGGCTCC AACGT CTC TGC TCCA TGC CCCAT GCA GGG TAC CCT GTC ACT GGT CGT GCT
Macaque ψRetnl  CAA GGGCTCC AGTGT CTC TGC TCCA TGC CCCAT GCA GGG AAC CCT GTC ACT GGT CGT GCT
O_baboon ψRetnl CAA GGGCTCC AACGT CTC TGC TCCA TGC CCCAT GCA GGG AAC CCT GTC ACT GGT CGT GCT
                                           EXON 2      >>>><<<<         EXON 3

                 C   G   Y   G   C   G   S   W   D   V   Q   L   D   T   T   C   H   C   Q   C
Human RETNL     TGT GGC TAT GGC TGT GGT TCG TGG GAT GTT CAG CTG GAA ACC ACC TGC CAC TGC CAG TGC
Human ψRETNL    TGC GGC TAT GGC TGT GGC TCC TGG GAT GTC CTA TGG GAA TCC ATC TGT CAC TGC CAG TGT
Chimp ψRetnl    TGT GGC TAT G-C TGT GGC TCC TGG GAT GTC CTA CGG GAA TCC ATC TGT CAC TGC CAG TGT
Orang ψRetnl    TGT GGC TAT GAC TGT GGC TCC TGG GAT GTC CTA TGG GAA TCC ATC TGT CAC TGC CAG TGT
Macaque ψRetnl  TGT GGC TAC GGT TGT GGC TCC TGG GAT GTC CTA TGG GAA TCC ATC TGT CAC TGC CAG TGT
O_baboon ψRetnl TGT GGC TAC GGT TGT GGC TCC TGG GAT GTC CTA TGG GAA TCC ATC TGT CAC TGC CAG TGT

                 S   V   V   D   W   T   T   A   R   C   C   H   L              T    *
Human RETNL     AGT GTG GTG GAC TGG ACC ACT GCC CGC TGC TGC CAC CTG --- --- --- ACC- TGA
Human ψRETNL    GTT GTT TTG GAT GGG GTC ACT GCC -TG CTC TGA GAT ATG AGA GCT AAG ACCA TGT
Chimp ψRetnl    GTT GTT TTG GAT GGG GTC ACT GCC -TG CTC TGA GAA ATG AGA GCT AAG ACCA TGA
Orang ψRetnl    GTT GTT TTG GAT GGG GTC ACT GCC -TC CTC TGA GAA ATG AGA GCT AAG ACCA TGA
Macaque ψRetnl  GTT GTT TTG GAC GGG GAC ACT GCT -TG CTC TGA GAA ATG GGA GCT AAG ACCA TGA
O_baboon ψRetnl GTT GTT TTA GAG GGG GAC ACT GCT -TG CTC TGA GAA ATG GGA GCT AAG ACCA TGA
```

S5 Fig. Alignment of processed *Retnl* pseudogenes from primates.

Alignment of human *RETNL* with *Retnl* pseudogenes from human (Human ψ*RETNL*), chimpanzee (Chimp ψ*Retnl*), orangutan (Orang ψ*Retnl*), macaque (Macaque ψ*Retnl*), and olive baboon (O_baboon ψ*Retnl*). Dashes are gaps introduced to maximize the alignment. The protein sequence of human RETNL is shown above the human *RETNL* coding sequence. DNA sequences are separated into codons. Base changes and gaps marked in red disrupt the coding sequence of the pseudogenes. Boundaries of the exons within the coding sequence of human *RETNL* gene are indicated by >>>> and <<<< below the sequences. No intron sequences are present in the pseudogenes, while the intron sequences in the human gene are not shown.
