# Supplementary material for: Evolution of the Vertebrate Resistin Gene Family
Source: PLoS One. 2015 Jun 15;10(6):e0130188. doi: 10.1371/journal.pone.0130188 (PMC4467842; doi:10.1371/journal.pone.0130188)
Supplement: S6 Fig — (PDF) [file pone.0130188.s006.pdf]

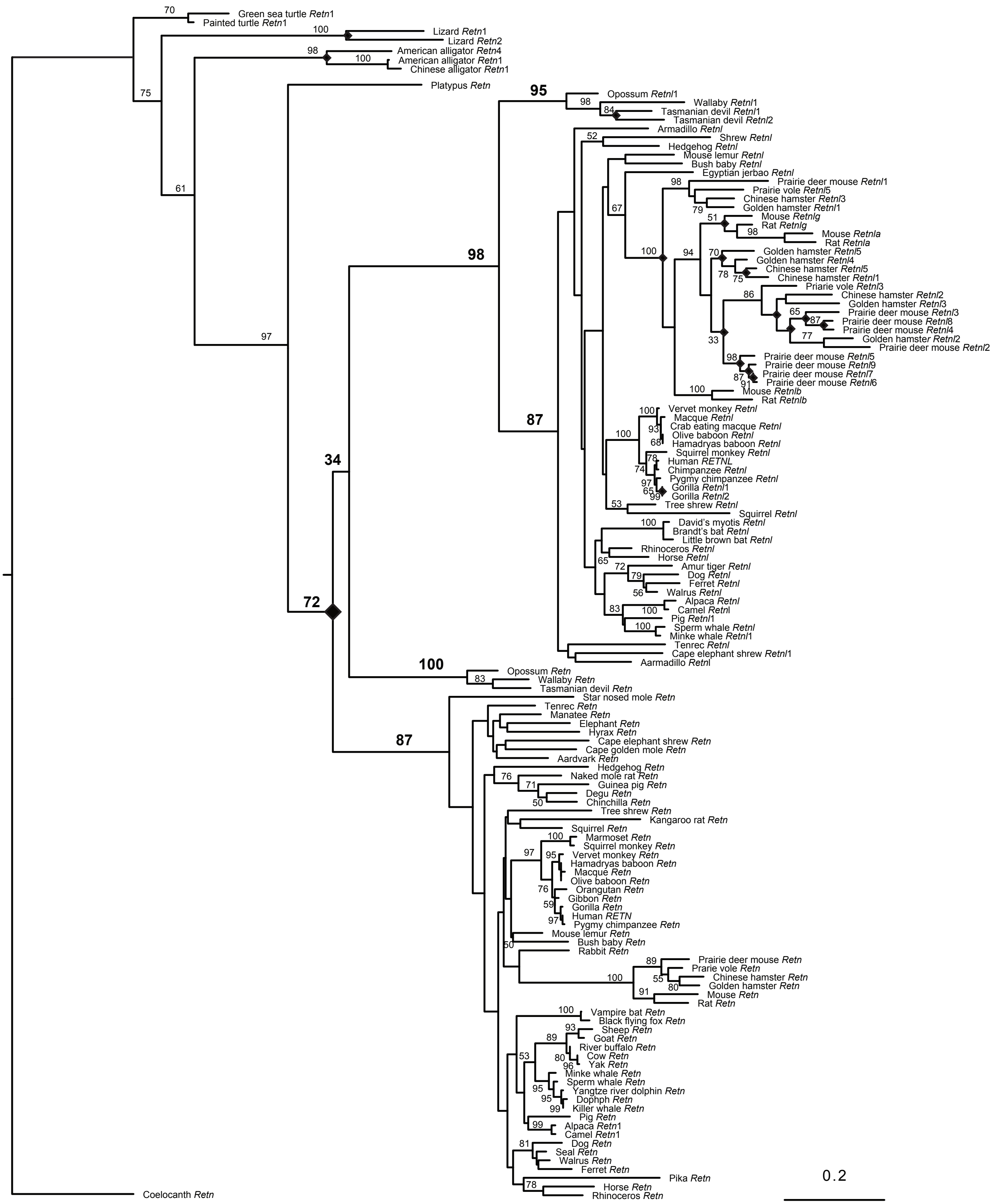

S6 Fig. Phylogeny of *Retn* and *Retnl* coding sequences generated by PhyML.
Phylogeny of *Retn* and *Retnl* coding sequences from diverse vertebrate species generated by maximum likelihood using PhyML 3.0. A similar phylogeny was generated by Bayesian methods (see Fig. 1). Phylogeny was rooted with the *Retn* sequence from the coelacanth. Numbers at the nodes indicate bootstrap support, with those for nodes in early mammalian evolution shown in bold. Branch lengths are proportional to the inferred amount of change, with the scale bar at the bottom. Diamonds indicate gene duplication events.
