# Supplementary material for: Evolution of the Vertebrate Resistin Gene Family
Source: PLoS One. 2015 Jun 15;10(6):e0130188. doi: 10.1371/journal.pone.0130188 (PMC4467842; doi:10.1371/journal.pone.0130188)
Supplement: S7 Fig — (PDF) [file pone.0130188.s007.pdf]

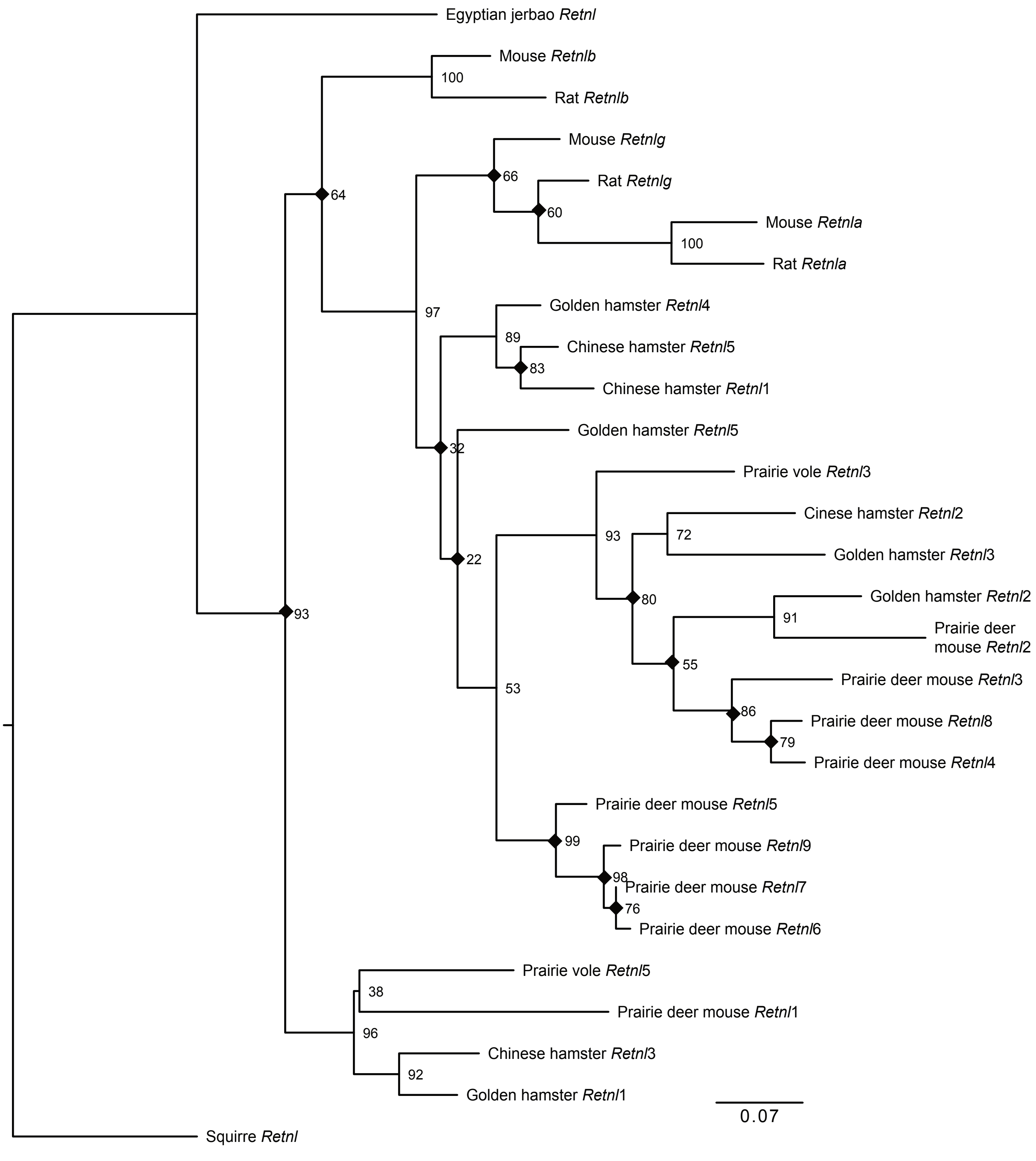

S7 Fig. Phylogenetic relationship of rodent *Retnl* genes.
Phylogeny of *Retnl* coding sequences from rodents generated by maximum likelihood using PhyML 3.0. Phylogeny was rooted with the *Retnl* sequence from the squirrel. Similar phylogenies were generated by Bayesian methods or if alternative non-rodent outgroups were used for rooting. Numbers at the nodes indicate bootstrap support. Branch lengths are proportional to the inferred amount of change, with the scale bar at the bottom. Diamonds indicate inferred gene duplication events.
