# Supplementary material for: Evolution of the Vertebrate Resistin Gene Family
Source: PLoS One. 2015 Jun 15;10(6):e0130188. doi: 10.1371/journal.pone.0130188 (PMC4467842; doi:10.1371/journal.pone.0130188)
Supplement: S8 Fig — (PDF) [file pone.0130188.s008.pdf]

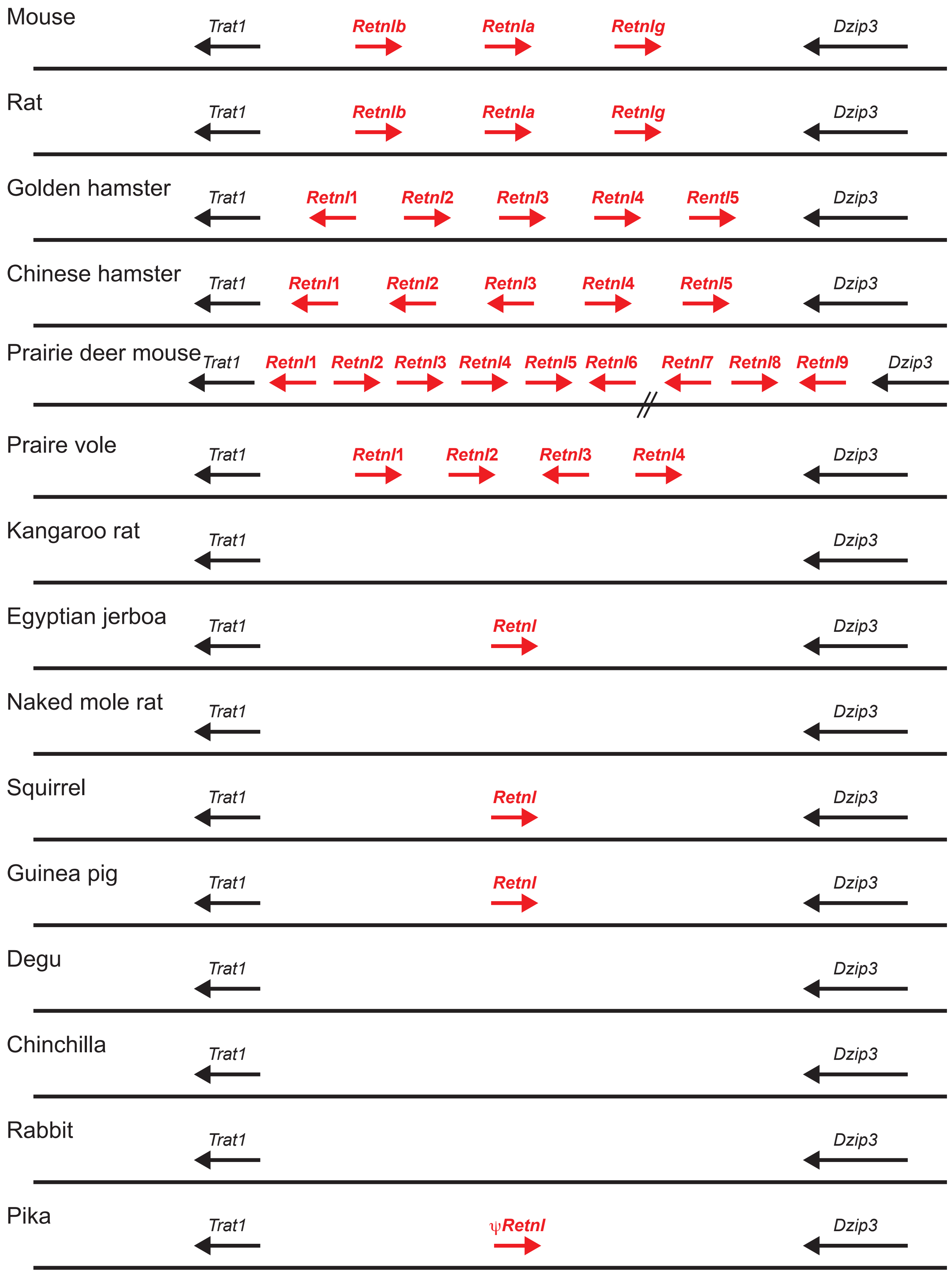

S8 Fig. Genomic organization of genes near rodent *Retnl* genes.
Retnl genes are labeled in red. See S1 Table for details on genomic locations. Gene sizes and distances between genes are not to scale. Arrowheads indicate direction of transcription. Gene symbols are: *Retnl*, resistin-like; *Trat1*, T cell receptor associated transmembrane adaptor 1; and *Dzip3*, DAZ interacting zinc finger protein 3.
