# Supplementary material for: Evolution of the Vertebrate Resistin Gene Family
Source: PLoS One. 2015 Jun 15;10(6):e0130188. doi: 10.1371/journal.pone.0130188 (PMC4467842; doi:10.1371/journal.pone.0130188)
Supplement: S1 Table — (PDF) [file pone.0130188.s009.pdf]

S1 Table. Genomic locations of *Retn* and *Retnl* genes in vertebrate genomes.

Species[1]	Gene ID[2]	Chromosome / Scaffold / Contig[3]	Position[4]	Intact[5]	missing exon(s)/ pseudogene mutation[6]
**MAMMALIA**					
^Human (*Homo sapiens*)					
RETN	ENSG00000104918	Chr 19	7,669,044-7,670,454	Y	
RETNL	ENSG00000163515	Chr 3	108,743,424-108,757,384	Y	
ψ*RETNL*	ENSG00000241777	Chr 3	108,725,440-108,725,536	ψ	processed
#Pygmy chimpanzee (*Pan paniscus*)					
Retn	XM_003810015.2	NW_003870285.1	1,066,294-1,067,867	Y	
Retnl	XM_003825063.2	NW_003870557.1	4,816,278-4,817,668	Y	
ψ*Retnl*	NA	NW_003870557.1	4,785,620-4,785,783	ψ	processed
^Chimpanzee (*Pan troglodytes*)					
Retn	ENSPTRG00000010398	Chr 19	7,771,892-7,773,035	I	exon 2
Retnl	ENSPTRG00000015195	Chr 3	112,047,820-112,049,456	Y	
ψ*Retnl*	NA	Chr 3	112,017,590-112,017,829	ψ	processed
^Gorilla (*Gorilla gorilla*)					
Retn1	ENSGGOG00000009545	Chr 19	7,882,168-7,883,562	Y	
*Retnl*1	ENSGGOG00000009854	Chr 3	107,960,184-107,961,586	Y	
*Retnl*2	ENSGGOG00000022847	Chr 3	107,938,605-107,940,146	Y	
ψ*Retnl*	NA	Chr3	107,913,093-107,912,854	ψ	processed
^Orangutan (*Pongo abelii*)					
Retn	ENSPPYG00000009481	Chr 19	7,833,331-7,835,057	Y	
Retnl	ENSPPYG00000013571	Chr 3	24,129,074-24,130,269	I	exon 2
ψ*Retnl*	NA	Chr 3	24,157,361-24,157,540	ψ	processed
^Gibbon (*Nomascus leucogenys*)					
Retn	ENSNLEG00000001150	SuperContig GL397524.1	328,749-330,091	Y	
Retnl		not found			
ψ*Retnl*	NA	SuperContig GL397284.1	15,245,386-15,245,505	ψ	processed
^Macaque (*Macaca mulatta*)					
Retn	NM_001243087.1	Chr 19	7,624,891-7,625,503	Y	
Retnl	ENSMMUG00000016534	Chr 2	28,858,306-28,859,910	Y	
ψ*Retnl*	NA	Chr 2	28,835,517-28,835,696	ψ	processed
^Olive baboon (*Papio anubis*)					
Retn	ENSPANG00000003097	Chr 19	7,331,696-7,333,562	Y	
Retnl	ENSPANG00000020527	Chr 2	28,588,747-28,590,541	Y	
ψ*Retnl*	NA	Chr 2	28,565,313-28,565,492	ψ	processed
#Crab-eating macaque (*Macaca fascicularis*)					
Retn	XM_005588649.1	Chr 19	8,005,677-8,006,249	I	exon 3
Retnl	XM_005548219	Chr 2	163,453,429-163,454,790	Y	
ψ*Retnl*	NA	Chr 2	163,477,743-16,347,7901	ψ	processed
*Hamadryas baboon (*Papio hamadryas*)					
Retn	NA	Contig486683_Contig22980	18,286-19,274	Y	

	Retnl	NA	Contig531662_Contig276623	107,392-108,759	Y	
	ψ*Retnl*	NA	Contig759846	2685-3004	ψ	processed
^Vervet monkey AGM (*Chlorocebus sabaeus*)						
	Retn	ENSCSAG00000008325	Chr 6	7,155,337-7,156,505	Y	
	Retnl	ENSCSAG00000006391	Chr 22	72,040,456-72,041,825	Y	
	ψ*Retnl*	NA	Chr 22	72,066,655-72,066,834	ψ	processed
#Marmoset (*Callithrix jacchus*)						
	Retn	XM_002761674.2	Chr 22	7,421,728-7,423,068	Y	
	ψ*Retnl*1	XM_003734925.2	Chr 15	88,060,266-88,061,677	ψ	aa 61 stop
	ψ*Retnl*2	NA	Chr 8	23,847,896-23,849,336	ψ	aa 61 stop
#Squirrel monkey (*Saimiri boliviensis*)						
	Retn	XM_003938888.1	NW_003943712.1	4,903,733-4,904,747	Y	
	Retnl	XM_003939399.1	NW_003943716.1	6,805,277-6,806,651	Y	
	ψ*Retnl*	NA	NW_003943716.1	6,800,482-6,800,586	ψ	processed
^Tarsier (*Tarsius syrichta*)						
	Retn		not found			
	Retnl	ENSTSYG00000007512	scaffold_84289	5,070-6,818	I	part exon 3
^Mouse lemur (*Microcebus murinus*)						
	Retn	ENSMICG00000008250	GeneScaffold_432	36,488-37,446	Y	
	Retnl	ENSMICG00000000243	GeneScaffold_3066	229,298-230,645	Y	
^Bushbaby (*Otolemur*						
	Retn	XM_003803420.1	Scaffold GL873821.1	521,189-522,207	Y	
	Retnl	ENSOGAG00000013671	Scaffold GL873650.1	4,381,560-4,382,963	Y	
#Tree shrew (*Tupaia belangeri*)						
	Retn	XM_006170219.1	NW_006208060.1	387,808-389,126	Y	
	Retnlb	XM_006164208.1	NW_006203439.1	573,609-574,883	Y	
^Mouse (*Mus musculus*)						
	Retn	ENSMUSG00000012705	Chr 8	3,655,770-3,660,110	Y	
	Retnla	ENSMUSG00000061100	Chr 16	48,842,552-48,844,461	Y	
	Retnlb	ENSMUSG00000022650	Chr 16	48,816,856-48,818,891	Y	
	Retnlg	ENSMUSG00000022651	Chr 16	48,872,608-48,874,498	Y	
^Rat (*Rattus norvegicus*)						
	Retn	ENSRNOG00000001001	Chr 12	4,364,007-4,365,746	Y	
	Retnla	ENSRNOG00000001955	Chr 11	57,705,933-57,707,432	Y	
	Retnlb	ENSRNOG00000032187	Chr 11	57,727,747-57,729,632	Y	
	Retnlg	ENSRNOG00000001943	Chr 11	57,674,751-57,676,090	Y	
#Chinese hamster (Cricetulus griseus)						
	Retn	XM_003507749.2	NW_003614420.1|	539,466-540,878	Y	
	*Retnl*1	XM_007640799.1	NW_003613771.1	899,647-900,961	Y	
	*Retnl*2	XM_007640798.1	NW_003613771.1	882,911-884,220	Y	
	*Retnl*3	XM_007640803.1	NW_003613771.1	830,186-831,693	Y	
	*Retnl*4	XM_007640796.1	NW_003613771.1	775,670-776,988	I	part exon 2
	*Retnl*5	XM_007625129.1	NW_003613771.1	763,134-764,492	Y	
#Golden hamster (*Mesocricetus auratus*)						

Retn	XM_005085269.1	NW_004801791.1	368,613-370,054	Y	
*Retnl*1	XM_005074772.1	NW_004801653.1	6,675,995-6,674,480	Y	
*Retnl*2	XM_005074817.1	NW_004801653.1	6,710,992-6,712,317	Y	
*Retnl*3	XM_005074818.1	NW_004801653.1	6,731,633-6,732,944	Y	
*Retnl*4	XM_005074773.1	NW_004801653.1	6,740,139-6,740,817	Y	
*Retlb*5	XM_005074775.1	NW_004801653.1	6,747,398-6,757,638	Y	
^Kangaroo rat (*Dipodomys ordii*)					
Retn	ENSDORG00000009639	GeneScaffold_643	39,638-45,246	Y	
Retnl		not found			
#Prairie deer mouse (*Peromyscus maniculatus bairdii*)					
Retn	XM_006970173.1	NW_006501038.1	3,702,444-3,703,754	Y	
*Retnl*1	XM_006996509.1	NW_006502104.1	743,913-745,258	Y	
*Retnl*2	XM_006996517.1	NW_006502104.1	684,884-686,203	Y	
*Retnl*3	XM_006996507.1	NW_006502104.1	698,870-700,180	Y	
*Retnl*4	XM_006996504.1	NW_006502104.1	712,346-713,658	Y	
*Retnl*5	XM_006996508.1	NW_006502104.1	716,967-718,311	Y	
*Retnl*6	XM_006996503.1	NW_006502104.1	644,406-645,970	Y	
*Retnl*7	XM_006989974.1	NW_006501475.1	3,868-5213	Y	
*Retnl*8	XM_006989975.1	NW_006501475.1	8,834-10,147	Y	
*Retnl*9	XM_006989977.1	NW_006501475.1	15,351-16,699	Y	
#Prairie vole (*Microtus ochrogaster*)					
Retn	XM_005371737.1	NW_004949223.1|	23,980-25,393	Y	
*Retnl*1	NA	Chr2	59,335,644-59,336,368	I	exon 1
*Retnl*2	XM_005345040.1	Chr 2	59,352,807-59,353,556	I	exon 1
*Retnl*3	XM_005345041.1	Chr 2	59,374,907-59,376,203	Y	
Retnl4	NA	Chr 2	59,388,202-59,388,322	I	exons 2 and 3
Retnl5	XM_005372257.1	NW_004949683.1	7,401-9,237	Y	
^Squirrel (*Icidomys tridecemlineatus*)					
Retn	ENSSTOG00000026972	JH393398.1	4,705,586-4,706,574	Y	
Retnl	ENSSTOG00000024867	Scaffold JH393455.1	2,401,109-2,402,637	Y	
#Naked mole-rat (*Heterocephalus glaber*)					
Retn	XM_004886644.1	NW_004624828.1	735,897-737,066	Y	
Retnl		not found			
^Guinea pig (*Cavia porcellus*)					
Retn	ENSCPOG00000010814	scaffold_42	14,907,205-14,908,327	Y	
Retnl	NA	scaffold_35	10,475,818-10,475,937	I	exons 1 and 2
#Degu (*Octodon degus*)					
Retn	XM_004644944	NW_004524790.1	1,722,494-1,723,845	Y	
Retnl		not found			
#Egyptian jerboa (*Jaculus jaculus*)					
Retn	NA	NW_004504357.1	18,027,788-18,027,895	I	exon 1
Retnl	XM_004663972	NW_004504380.1	10,689,623-10,691,344	Y	
#Chinchilla (*Chinchilla lanigera*)					
Retn	XM_005413172	NW_004955563.1	1,608,988-1,607,911	Y	
Retnl		not found			

^Rabbit (*Oryctolagus cuniculus*)					
Retn	ENSOCUG00000025105	Scaffold GL018767	807,596-810,442	Y	
Retnl		not found			
^Pika (*Ochonta princeps*)					
Retn	ENSOPRG00000004676	GeneScaffold_630	5,252-6,456	Y	
ψ*Retnl*	ENSOPRG00000000116	GeneScaffold_5075	233,304-236,748	ψ	aa 26 and 73 stop
^Cow (*Bos taurus*)					
Retn	ENSBTAG00000004716	Chr 7	17,737,516-17,738,903	Y	
ψ*Retnl*	NA	Chr 1	53,928,539-53,929,845	ψ	exon 1 insertion
#Yak (*Bos mutus*)					
Retn	XM_005890748	NW_005393218.1	199,380-200,449	Y	
ψ*Retnl*	NA	NW_005395138.1	576,534-577,838	ψ	exon 1 insertion
#River buffalo (*Bubalus bubalis*)					
Retn	XM_006046759	NW_005783911.1	706,285-707,263	Y	
ψ*Retnl*	XM_006080977	NW_005785628.1	200,380-201,684	ψ	exon 1 insertion
#Chiru (*Pantholops hodgsonii*)					
Retn	XM_005959014	NW_005807695.1	745,968-747,032	I	part exon 3
ψ*Retnl*	XM_005970427	NW_005812447.1	1,926,546-1,927,853	ψ	exon 1 insertion
^Sheep (*Ovis aeries*)					
Retn	ENSOARG00000002291	Chr 5	14,185,287-14,186,266	Y	
ψ*Retnl*	ENSOARG00000019039	Chr 1	171,975,815-17,197,6790	ψ	exon 1 insertion
#Goat (*Capra hircus*)					
Retn	XM_005682406	NC_022299.1|	13,027,984-13,028,962	Y	
ψ*Retnl*	XM_005674969	NC_022293.1|	52,225,799-52,227,098	ψ	exon 1 insertion
^Bottlenose dolphin (*Tursiops truncatus*)					
Retn	ENSTTRG00000001641	scaffold_84971	6,013-7,002	Y	
*Retnl*1	ENSTTRG00000009745	GeneScaffold_2303	224,136-224,946	I	exon 1
*Retnl*2	NA	GeneScaffold_2303	218,951-219,079	I	exons 1and 2
#Sperm whale (*Physeter catodon*)					
Retn	XM_007107097	NW_006713474.1	454,501-455,485	Y	
Retnl	XM_007119293	NW_006716203.1	178,710-179,830	Y	
#Minke whale (*Balaenoptera acutorostrata scammoni*)					
Retn	XM_007169061	NW_006725554.1	2,622,784-2,623,768	Y	
*Retnl*1	XM_007187131	NW_006728571.1	8,849,655-8,850,780	Y	
*Retnl*2	NA	NW_006728571.1	8,855,983-8,856,115	I	exons 1and 2
*Retnl*3	NA	NW_006728571.1	8,874,413-8,874,539	I	exons 1 and 2
#Yangtze River dolphin (*Lipotes vexillifer*)					
Retn	XM_007460605	NW_006787698.1	3,208,113-3,209,096	Y	
*Retnl*1	NA	NW_006776646.1	599,766-600,572	I	exon 1
*Retnl*2	NA	NW_006776646.1	605,855-605,983	I	exons 1 and 2
#Killer whale (*Orcinus orca*)					
Retn	XM_004277299	NW_004438506.1	3,124,499-3,125,474	Y	
Retnl1	NA	NW_004438463.1	5,753,014-5,753,800	I	exon 1

	Retnl2	NA	NW_004438463.1	5,758,870-5,758,998	I	exons 1 and 2
^Pig (*Sus scrofa*)						
	Retn	ENSSSCG00000013575	Chr 2	71,871,225-71,872,652	Y	
	Retnl1	ENSSSCG00000011939	Chr 13	159,846,499-159,847,802	Y	
	Retnl2	NA	Chr 13	159,863,371-159,863,550	I	exons 1 and 2
#Alpaca (*Vicugna pacos*)						
	Retn	XM_006215917.1	NW_005882762.1	4,123,346-4,124,338	Y	
	ψ*Retn*	NA	NW_005882714.1	15,149,005-15,149,218	ψ	processed
	Retnl	XM_006215917.	NW_005882931.1	1,651,987-1,653,326	Y	
#Camel (*Camelus ferus*)						
	Retn	XM_006177569	NW_006210496.1	137,340-138,332	Y	
	ψ*Retn*	NA	NW_006217862.1		ψ	processed
	Retnl	XM_006192297	NW_006211894.1	572,640-574,003	Y	
^Panda (*Ailuropoda melanoleuca*)						
	Retn	ENSAMEG00000008546	Scaffold GL192871.1	905,622-906,509	I	exon 3
	Retnl	ENSAMEG00000020230	Scaffold GL193448.1	17,662-19,023	I	part exon 3
^Ferret (*Mustela putorius*)						
	Retn	ENSMPUG00000008210	Scaffold GL897062.1	3,709,148-3,710,397	Y	
	Retnl	ENSMPUG00000010050	Scaffold GL897041.1	4,915,616-4,918,635	Y	
#Dog (*Canis familiaris*)						
	Retn	XM_849220.3	Chr 20	52,434,462-52,435,483	Y	
	Retnl	XM_003434071.2	Chr 33	13,980,835-13,982,262	Y	
	ψ*Retnl*	NA	Chr 8	63,362,240-63,362,576	ψ	processed
#Cat (*Felis catus*)						
	Retn	XM_003981823	Chr A2	5,999,203-6,000,747	I	exon 3
	ψ*Retnl*	XM_004001331.1	Chr C2	55,390,048-55,391,702	ψ	aa 51 stop
#Amur tiger (*Panthera tigris altaica*)						
	Retn	NA	NW_006712030.1	950,652-951,152	I	exon 3
	Retnl	XM_007098588	NW_006712676.1	3,881,621-3,883,275	Y	
#Weddel seal (*Leptonychotes weddellii*)						
	Retn	XM_006733815	NW_006383531.1	43,442-44,410	Y	
	ψ*Retnl*	XM_006729723	NW_006383215.1	1,782,495-1,783,527	ψ	aa 76 stop
#Pacific walrus (*Odobenus rosmarus divergens*)						
	Retn	XM_004412162	NW_004450879.1	775,791-776,777	Y	
	Retnl	XM_004391620	NW_004450265.1	3,436,969-3,438,379	Y	
^Horse (*Equus caballus*)						
	Retn	ENSECAG00000016421	Chr 7	4,594,688-4,595,214	Y	
	Retnl	NA	Chr 19	48,176,676-48,178,875	Y	
#Rhinoceros (*Ceratotherium simum simum*)						
	Retn	XM_004443519	NW_004454320.1	480,784-481,899	Y	
	Retnl	NA	NW_004454216.1	11,637,728-11,636,242	Y	
^Vampire bat (*Pteropus vampyrus*) (Megabat)						

Retn	ENSPVAG00000006942	GeneScaffold_373	56,401-57,655	Y	
Retnl		not found			
^Little brown bat *(Myotis lucifugus*) (Microbat)					
Retn		not found			
Retnl	ENSMLUG00000001459	Scaffold GL429834	5,397,005-5,398,425	Y	
#Black flying fox (*Pteropus alecto*)					
Retn	XM_006917236	NW_006440605.1	793,647-794,881	Y	
ψ*Retnl*	XM_006905490	NW_006430735.1	2,832,695-2,834,077	ψ	aa 61 stop M1I
#Brandt's bat (*Myotis brandtii*)					
Retn		not found			
Retnl	XM_005856994	NW_005353584.1	942,826-944,233	Y	
#David's myotis (*Myotis davidii*)					
Retn		not fond			
Retnl	XM_006759569	NW_006287821.1	738,079-739,479	Y	
*Shrew (*Sorex araneus*)					
Retn		not found			
Retnl	NA	JH798168.1	17,765,525-17,768,234	Y	
*Hedgehog (*Erinaceus europaeus*)					
Retn	XM_007532841.1	JH835913.1	836318-836766	Y	
Retnl	XM_007522174.1	JH835457.1	3,962,253-3,966,354	Y	
#Cape golden mole (*Chrysochloris asiatica*)					
Retn	XM_006868990	NW_006408643.1	198,220-199964	Y	
Retnl		not found			
#star-nosed mole (*Condylura cristata*)					
Retn	XM_004695817	NW_004567873.1	1,453-2,339	Y	
Retnl	NA	NW_004567104.1	42,325,702-42,326,434	I	exons 1and 2
#Cape elephant shrew (*Elephantulus edwardii*)					
Retn	XM_006902673	NW_006400188.1	207,446-208,974	Y	
*Retnl*1	XM_006895349	NW_006399915.1	664,712-666,226	Y	
*Retnl*2	NA	NW_006399915.1	620,288-620,439	I	exons 1 and 2
^Elephant (*Loxodonta africana*)					
Retn	ENSLAFG00000028376	SuperContig scaffold_114	1,702,179-1,703,990	Y	
*Retnl*1	NA	SuperContig scaffold_112	68,149-69,923	I	exon 2
*Retnl*2	NA	SuperContig scaffold_112	60,480-60,610	I	exons 1 and 2
#Florida manatee (*Trichechus manatus latirostris*)					
Retn	XM_004378593	NW_004443993.1	5,647,578-5,649,299	Y	
ψ*Retnl*1	XM_004378172	NW_004443991.1	12,215,574-12,217,164	ψ	aa 96 stop
*Retnl*2	NA	NW_004443991.1	12,208,609-12,208,473	I	exons 1 and 2
^Hyrax (*Procavia capensis*)					
Retn	ENSPCAG00000007052	scaffold_88523	4,508-6,627	Y	

Retnl		not found			
^Armadillo (*Dasypus vovemcinctus*)					
Retn		not found			
Retnl	ENSDNOG00000009661	Scaffold JH578677.1	1,164,847-1,171,534	Y	
^Sloth (*Choloepus hoffmanni*)					
Retn		not found			
Retnl		not found			
#Tenrec (*Echinops telfairi*)					
Retn	XM_004717352.1	NW_004558847.1	570,363-571,918	Y	
Retnl	ENSETEG00000012063	GeneScaffold_5888	5,999-7,885	Y	
*Aardvark (*Orycteropus afer afer*)					
Retn	NA	JH864123.1	512,442-513,936	Y	
Retnl	NA	JH863884.1	1,313,138-1,314,754	Y	
^Opossum (*Monodelphis domestica*)					
Retn	NA	Chr 3	463,160,361-463,160,540	Y	
*Retnl*1	ENSMODG00000018074	Chr 4	68,636,528-68,643,121	Y	
ψ*Retnl*2	ENSMODG00000028453	Chr 4	68,656,866-68,657,909	ψ	frame shift exon 3
*Retnl*3	NA	Chr 4	68,697,507-68,697,634	I	exons 1 and 2
^Wallaby (*Macropus eugenii*)					
Retn	ENSMEUG00000015810	Scaffold132767	5,992-6,892	Y	
Retnl1	ENSMEUG00000010781	Scaffold32582	20,918-21,946	Y	
Retnl2	NA	Scaffold45912	6,337-6,479	I	exons 1 and 2
^Tasmanian devil (*Sarcophilus harrisii*)					
Retn	NA	Scaffold GL849142.1	17,295-18,172	Y	
*Retnl*1	ENSSHAG00000015202	Scaffold GL849569.1	1,721,480-1,722,688	Y	
*Retnl*2	ENSSHAG00000015983	Scaffold GL849569.1	1,977,587-1,978,863	Y	
^Platypus (*Ornithorhynchus anatinus*)					
Retn	ENSOANG00000007858	SuperContig Contig15629	1,270-2,472	Y	
**AVES**					
^Chicken (*Gallus gallus*)					
Retn		not found			
^Turkey (*Meleagris gallopavo*)					
Retn		not found			
^Duck (*Anas platyrhynchos*)					
Retn		not found			
#Rock pigeon (*Columba livia*)					
Retn		not found			
#Saker falcon (*Falco cherrug*)					

Retn		not found			
#Peregrine falcon (*Falco peregrinus*)					
Retn		not found			
#Medium ground-finch (*Geospiza fortis*)					
Retn		not found			
*Budgerigar (*Melopsittacus undulatus*)					
Retn		not found			
#Tibetan ground-tit (*Pseudopodoces humilis*)					
Retn		not found			
#White-throated sparrow (*Zonotrichia albicollis*)					
Retn		not found			
^Zebra Finch (*Taeniopygia guttata*)					
Retn		not found			
^Flycatcher (*Ficedula albicollis*)					
Retn		not found			
**SAURIA (except Aves)**					
^Anole lizard (*Anolis carolinensis*)					
*Retn*1	ENSACAG00000029691	Chr 2	102,944,753-102,953,351	Y	
*Retn*2	ENSACAG00000028837	Chr2	102,966,277-102,968,976	Y	
#Burmese python (*Python bivittatus*)					
*Retn*1	NA	NW_006532674.1	373,611-374,095	I	exon 1
*Retn*2	XM_007443736.1	NW_006540298.1	14,071-12,622	I	exon 1
#American alligator (*Alligator mississippiensis*)					
*Retn*1	XM_006265086	NW_006227722.1	67,952-69,161	Y	
ψ*Retn*2	NA	NW_006227722.1	64,202-64,421	ψ	aa 49 64 91 stop
*Retn*3	NA	NW_006227722.1	60,246-60,401	I	exons 1 and 2
*Retn*4	XM_006265093.1	NW_006227722.1	50,767-51,928	Y	
#Chinese alligator (*Alligator sinensis*)					
*Retn*1	XM_006036482	NW_005842966.1	20,101-23,008	Y	dupicated exon 2
*Retn*2	NA	NW_005842966.1	16,158-17,061	I	exon 2 and part 1
*Retn*3	NA	NW_005842966.1	12,155-12,292	I	exons 1 and 2
*Retn*4	NA	NW_005842966.1	2,934-3,939	I	exon 2 and part 3
#Green sea turtle (*Chelonia mydas*)					
*Retn*1	XM_007072480	NW_006709926.1	13,411-16,441	Y	
*Retn*2	NA	NW_006709926.1	28,613-28,486	I	exons 1 and 2
*Retn*3	NA	NW_006709926.1	44,282-44338	I	exons 1 2 and part 3
*Retn*4	NA	NW_006709926.1	126,617-126,750	I	exons 1 and 2

#Western painted turtle (*Chrysemys picta bellii*)					
*Retn*1	XR_257304/ NA	NW_007281833.1	55,461-57,956	Y	
*Retn*2	XM_008177007.1	NW_007281875.1	41,335-41,964	I	exon 1
*Retn*3	NA	NW_007281875.1	7,647-8,271	I	exon 1
#Chinese softshell turtle (*Pelodiscus sinensis*)					
Retn	XM_006132041.1	NW_005857693.1	1,277,041-1,278,605	Y	part exon 3
**AMPHIBIA**					
#Xenopus (*Xenopus tropicalis*)					
Retn	XM_004920544.1	NW_004669984.1	78-995	I	exon 1
**ACTINISTIA**					
#Coelocanth (*Latimeria chalumnae*)					
Retn	XM_006002924.1	NW_005819879.1	882,043-898,967	Y	
**ACTINOPTERYGII**					
^Zebrafish (*Danio rerio*)					
Retn		not found			
^Cod (*Gadus morhus*)					
Retn		not found			
^Fugu (*Takifugu rubripes*)					
Retn		not found			
^Tetraodon (*Tetraodon nigroviridis*)					
Retn		not found			
^Tilapia *(Oreochromis niloticus*)					
Retn		not found			
^Stickleback (*Gasterosteus aculeatus*)					
Retn		not found			
^Medaka (*Oryzias laptipes*)					
Retn		not found			
^Platyfish (*Xiphorus maculatus*)					
Retn		not found			
#Mexican tetra (*Astyanax mexicanus*)					
Retn		not found			
#Tongue sole (*Cynoglossus semilaevis*)					
Retn		not found			
#Burton's mouthbrooder (*Haplochromis burtoni*)					
Retn		not found			

#Zebra mbuna (*Maylandia zebra*)	
Retn	not found
#Lyretail cichlid (*Neolamprologus brichardi*)	
Retn	not found
#Amazon molly (*Poecilia formosa*)	
Retn	not found
#Guppy (*Poecilia reticulata*)	
Retn	not found
#*Pundamilia nyererei*	
Retn	not found
#Spotted gar (*Lepisosteus oculatus*)	
Retn	not found
**CHONDICHTHYES**	
#Elephant shark (*Callorhinchus milii*)	
Retn	not found
**AGNATHA**	
^Lamprey (*Petromyzon marinus*)	
Retn	not found

[1] Genomic data from: ^ Ensembl, * preEnsembl, # NCBI Genomes
[2] ENSEMBL gene ID or NCBI Acession
[3] Genomic source, chromosome, Gene scaffold or Genomic contig
[4] Location within the genomic sourse from start to stop codon
[5] Is codong sequence intact, Y = yes, I = incomplete (missing genomic sequence), ψ = pseudogene due to inactivating mutation
[6] Sequences (exons) missing from incomplete genes or mutations that yield pseudogenes, processed indicates a retrotransposed pseudogene
