# Supplementary material for: Evolution of the Vertebrate Resistin Gene Family
Source: PLoS One. 2015 Jun 15;10(6):e0130188. doi: 10.1371/journal.pone.0130188 (PMC4467842; doi:10.1371/journal.pone.0130188)
Supplement: S4 Fig — (PDF) [file pone.0130188.s004.pdf]

```
           M   K   P   T   S   C   F   L   L   I   L   I   L   L
Pig       ATG AAG CCA ACT TCT TGC TTC CTT CTT ATC CTC ATC CTC CTT -------------------------------
Cow       ATG AAG CCA ACC TCT TGC TTC CTT CTC ATC ACC ATC CCT CTT GAAGTGAAGTGAAGTGAAAGTCATTCTGTCA
Yak       ATG AAG CCA ACC TCT TGC TTC CTT CTC ATC ACC ATC CCT CTT GAAGTGAAGTGAAGTGAAAGTCGTTCTGTCA
Buffalo   ATG AAG CCA ACC TCT TGC TTT CTT CTC ATC ACT ATC CCT CTT GAAGTGAAGTGAAGTGAAAGTCGCTCTGTCA
Chiru     ATG AGG CCA ACC TCT TGC TTC CTT CTC ATC ACC ATC CCT CTT GAAGTGAAGTGAAGTGAAAGTCGCTCTGTCA
Sheep     ATG AAG CCA ACC TCT TGC TTC CTT CTC ATC ACC ATC CCT CTT GAAGTGAAGTGAAGTGAAAGTCGCTCTGTCA
Goat      ATG AAG CCA ACC TCT TGC TTC CTT CTC ATC ACC ATC CCT CTT GAAGTGAAGTGAAGTGAAAGTCGCTCTGTCA
                                                                                      ^^^^

Pig       ---------------------------------------------------------------------------------------
Cow       TGTCCGACTCTTTGTGACCCCATGGACTCTCCATGGAATTCTACAGGCCAGAATACTGGAGTGGGTAGCCTTTCCCTTCTCCAGGGG
Yak       TGTCCGACTCTTTGTGACCCCATGGACTCTCCATGGAATTCTACAGGCCAGAATACTGGAGTGGGTAGCCTTTCCCTTCTCCAGGGG
Buffalo   TGTCTGACTCTTTGCGACCCCATGGACTCTCCACGGAATTCTACAGGCCAGAATACTGGAGTGGGTAGCCTTTCCCTTCTCCAGGGG
Chiru     TGTCCGACT---TGCAACCCCATGGACTCTCCACGGAATTCTACAGGCCAGAATACTGGAGTGGGTAGCCATTCCCATCTCCAGGTG
Sheep     TGTCCGACT---TGCAACCCCATGGACTCTCCATGGAATTCTACAGGCCAGAATACTGGAGTGGGTAGCC--------GTCCAGGTG
Goat      TGTCTGACT---TGCAACCCCATGGACTCTCCACGGAATTCTACAGGCCAGAATACTGGAGTGGGTAGCC--------GTCCAGGTG
                                                Repetitive DNA

Pig       ---------------------------------------------------------------------------------------
Cow       ATCTTCCCAACCCCTGGATAGAACCCAGGTCTCCTGCATTGCAGGCAGATTCTTTACCAACTGAGCCACAAGGGAAGCCCAAGAACA
Yak       ATCTTCCCAACCCAGGGATAGAACCCAGGTCTCCTGCATTGCAGGCAGATTCTTTACCAACTGAGCCACAAGGGAAGCCCAAGAACA
Buffalo   ATATTCCCAACCCAGGGATAGAACCCAGGTCTCCTGCATTGCAGGCGGATTCTTTACCAACTGAGCCACAAGGGAAGCCCAAGAACA
Chiru     ATCTTCTCAACCCAGGGATCAAACTCAGGTCTCCCGCATTACAGGCAGATTCTTTACCAGCTGAGCCACAAGGGAAGCCCAATAACA
Sheep     ATCTTCTCAACCCAGGGATCGAACCCAGGTCTCCTGCATTGCAGGCAGATTCTTTACCAGCTGAGCCACACGGGAAGCCCAAGAACA
Goat      ATCTTCTCAACCCAGGGATCGAACCCAGGTCTCCCACATTGCAGGCAGATTCTTTACCAGCTGAGCCACATGGGAAGCCCAAGAACA
                                                Repetitive DNA

                                     L   Q   L   I   I   A   R   G   S   Q   C   S   L   D   S   L
Pig       ----------------------- CTT CAG TTG ATA ATC GCA AGG GGT TCT CAG TGT TCC TTA GAC TCC CTT
Cow       CTGGAGTGGGTAACCTATCCCTT CTC CA- -TG ATA ATC CCA TGG GGT GCT CCA TAC CTC TTA GAC TCC ATT
Yak       CTGGAGTGGGTAGCCTATCCCTT CTC CA- -TG ATA ATC CCA GGG GGT GCT CCA TAC CCC TTA GAC TCC ATT
Buffalo   CTGGAGTGGGTAG------CCTT CTC CA- -TG ATA ATC CCA TGG GGT GTT CCA TAT TCC TTA GAC TCC ATT
Chiru     CTGAAGTGGATAGCCTATCCCTT CTC CA- -TG ATA ATC CCA TGG GGT GCT CCA CAC TCC TTA GAC TCC ATT
Sheep     CTGGAGTGGGTAGCCTATCCCTT CTC CA- -TG ATA ATC CCA TGG GGT GCT CCA TAC TCC TTA AAC TGC ATT
Goat      CTGGAGTGGGTAGCCTATCCCTT CTC CA- -TG ATA ATC CCA TGG GGT GCT CCA TAC TCC TTA GAC TCC ATT
                                     ^^^^

           V   D   K   K   I   R   D   A   L   N   R   L   E   L   N   P   S   A   P   R   K   R
Pig       GTG GAC AAA AAG ATA AAG GAT GCT CTC AAT CGT CTA GAG CTC AAT CCC TCT GCT CCA AGA AAG AGG
Cow       ATC GAT AAA AAG ATA AAA AAT GCT CTC ATT --- --- GAG CTC AA- GCC TCT GCC ACA GAG AAG ATG
Yak       ATT GAT AAA AAT ATA AAA AAT GCT CTC ATT --- --- GAG CTC AA- GCC TCT GCC ACA GAG AAG ATA
Buffalo   ATC AAT AAA AAG ATA AAA AAT ACT CTC ATT --- --- GAG CTC AA- GCC TCT GCC ACA GAG AAG ATG
Chiru     ATC GAC AAA AAG ATA AAA AAT CCT CTC ACT GGT CTA GAG CTC AA- GCC TCT GCC ACA GAG AAG ATG
Sheep     ATC GAT AAA AAG ATA AAA AAT --- --- --- --- --- -AG CTC AA- GCC TCT GCC ACA GAG AAG ATG
Goat      ATC GAT AAA AAG ATA AAA AAT CCT CTT ATT GGT TTA GAG CTC AA- GCC TCT GCC ACA GAG AAG ATG
                                     EXON1                >>>><<<<        EXON 2

           I   S   C   F   S   V   K   N   S   G   K   L   S   S   C   P   A   G   T   V   V   T
Pig       ATC TCA TGT TTC AGT GTC AAA AAC TCA GGC AAA CTG TCT TCC TGC CCT GCA GGG ACC GTT GTC ACT
Cow       AGC TCA TGC TTC AGT GTC AAG AAC TCA GGC AAA CTG TCC TCC TGC CCT --- -GG TCT GTT GTC ACT
Yak       AGC TCA TGC TTC AGT GTC AAG AAC TCA GGC AAA CTG TCC TCC TGC CTT CC- -GG TCT GTT GTC ACT
Buffalo   AGC TCA TGT TTT AGT GTC AAG AAC TCG GGC AAA CTG TAC TCC TAC CTT CC- -GG TCT GTT GTC ACT
Chiru     AGC TCA TGT TTC AGT GTC AAG AAC TCA GGC AAA CTG TCC TCC TGC CCT GCA GGG TCT GTT GTC ACT
Sheep     AGC TCA TGT TTC AGT GTC AAG AAC TCA GGC AAA CTG TCT TCC TGC CCT GCA GGG TCT GTT GTC ACT
Goat      AGC TCA TGT TTC AGT GTC AAG AAC TCA GGC AAA CTG TCC TCC TGC CCT GCA GGG TCA GTT GTC ACT
                                                              EXON 2      >>>><<<<    EXON 3

           G   C    A   C   G   Y   A   C   G   S   W   D   V   Q   H   E   T   T   C   H   C
Pig       GGC TGT- GCT TGT GGC TAT GCC TGT GGT TCC TGG GAT GTC CAG CAT GAA ACC ACA TGC CAC TGC
Cow       GGC TGTA GCT TGT GGC TAT ACC TGT GGC TCC TGG GAC ATC CGG GGG AAA ACC ACG TGT CAC AGC
Yak       GGC TGTA GCT TGT GGC TAT ACC TGT GGC TCC TGG GAC ACC TGG GGG AAA TCC ACG TGT CAC AGC
Buffalo   GGC TGTA GCT TGT GGC TAT CCC TGT GGC TCT TGG GAC ATC CAG GGG GAA ACC ACG TGT CAC GGC
Chiru     GGC TGT- GCT TGT GGC TAT ACT TGT GGC TCC TGG GAC ACT TGG GGG AAA ACC ACG TGT CAC GGC
Sheep     GGC AGT- GCT TGT GGC TAT ACC TGT GGC TCC TGG GAC ATC TGG GGG AAA ACC ATG TGT CAC GGC
Goat      GGC TGT- GCT TGT GGC TAT ACC TGT GGT TCC TGG GAC ATC TGG GGG AAA ACC ACG TGT CAC GGC

           Q   C   N   T   A   D   W   T    A   A   R   C   C   R   L   T   *
Pig       CAG TGC AAC ACA GCA GAC TGG ACC- GCT GCC CGC TGC TGC CGC CTG ACA TGA
Cow       CAG TGC AAC CCA GTA GAC TGG ACCC ACT GCC --- TGC TGC TGT C-- ATC TGA
Yak       CAG TGC AAC CCA GTA GAC TGG ACCC ACT GCC --- TGC TGC TGC C-- ATC TGA
Buffalo   CAG TGC AAC CCA GTA GAC TGG ACCC ACT GCC --- TGC TGC TGC C-- ATC TGA
Chiru     CAG TGC AGC CCA GTA GAC TGG ACC- ACT GCC --- TGC TGC TGC C-- ATC TGA
Sheep     CAG TGC AGC CCA GTA GAC TGG ACC- ACT GCC --- TGC TGC TGC C-- ATC TGA
Goat      CAG TGC AGC CCA GTA GAC TGG ACC- ACT GCC --- TGC TGC TGC C-- ATC TGA
```

S4 Fig. Inactivation of ruminant artiodactyl *Retnl* by insertion of a repetitive DNA element.
Alignment of *Retnl* pseudogenes from ruminant artiodactyls (cow, yak, buffalo (river buffalo), chiru, sheep, and goat) with an intact pig *Retnl* coding sequence. Dashes are gaps introduced to maximize the alignment. The protein sequence of pig Retnl is shown above the DNA sequences, which are separated into codons. Boundaries of the exons within the coding sequence of *Retn* genes are indicated by >>>> or <<<< below the sequences. A Bov-tA2 repetitive element identified by RepeatMasker [27] is located between the bases marked with ^^^^ and is labeled as repetitive DNA. Base changes and gaps marked in red disrupt the coding sequence of the pseudogenes. Intronic sequences in the genes are not shown.
